# Analysis of Mammalian rDNA Internal Transcribed Spacers

**DOI:** 10.1371/journal.pone.0079122

**Published:** 2013-11-19

**Authors:** Annette W. Coleman

**Affiliations:** Division of Biology and Medicine, Brown University, Providence, Rhode Island, United States of America; Australian Museum, Australia

## Abstract

Nuclear rDNA Internal Transcribed Spacers, ITS1 and ITS2, are widely used for eukaryote phylogenetic studies from the ordinal level to the species level, and there is even a database for ITS2 sequences. However, ITS regions have been ignored in mammalian phylogenetic studies, and only a few rodent and ape sequences are represented in GenBank. The reasons for this dearth, and the remedies, are described here. We have recovered these sequences, mostly >1 kb in length, for 36 mammalian species. Sequence alignment and transcript folding comparisons reveal the rRNA transcript secondary structure. Mammalian ITS regions, though quite long, still fold into the recognizable secondary structure of other eukaryotes. The ITS2 in particular bears the four standard helix loops, and loops II and III have the hallmark characters universal to eukaryotes. Both sequence and insertions/deletions of transcript secondary structure helices observed here support the four superorder taxonomy of Placentalia. On the family level, major unique indels, neatly excising entire helices, will be useful when additional species are represented, resulting in significant further understanding of the details of mammalian evolutionary history. Furthermore, the identification of a highly conserved element of ITS1 common to warm-blooded vertebrates may aid in deciphering the complex mechanism of RNA transcript processing. This is the last major group of terrestrial vertebrates for which rRNA ITS secondary structure has been resolved.

## Introduction

In 1956, Romer [1] recognized 15 orders of Eutheria, placental mammals, and this has now grown to 18, 17 of which include living species (Placentalia) (e.g. [2,3]). Over the past dozen years, the gradual recognition of the historical role of biogeography in post-Cretaceous mammalian evolution and the rise of molecular methods of analysis have resulted in today’s usage of four superorders to include all Eutheria. The four are the South American (Xenarthra), the African (Afrotheria), and the northern continental groups (Laurasiatheria), plus the conglomerate of primates and rodents (Euarchontoglires). The root of the eutherian tree is still uncertain; no single superorder is consistently basal in tree reconstructions (see [3-5]). Although the relative placement of some orders is still controversial, their assignment to the four superorders seems unimpeachable.

Molecular phylogenetic analyses have primarily utilized total mitochondrial sequences [6] and sequences from >3,000 nuclear genes [5,7]. In addition to these sequence comparison methods, there are several striking examples of multicodon indels supporting the monophyly of the Afrotheria and/or Afrotheria + Xenarthra [8-10]. Yet the most widely used nucleotide sequences for eukaryote phylogenetic reconstructions [11,12], the two Internal Transcribed Spacer regions of the nuclear ribosomal repeats, remain unexamined from this perspective in mammals.

The internal transcribed spacers are the ITS1, between the nuclear small subunit rRNA gene and the 5.8S gene, and the ITS2, between the 5.8S gene and the large subunit rRNA gene. These two spacers are transcribed in their entirety in the very long initial RNA transcript, and then the primary transcript undergoes a complex processing in the nucleolus that rapidly eliminates the nucleotides derived from both the ITS and external transcribed spacer regions, leaving the rRNA SSU, 5.8S and LSU products intact. The spacers have been of great phylogenetic value both because of their sequence and of their secondary structure, which derive from the relatively conserved helix basal pairings.

Despite the wide usage of the ITS for phylogenetic comparisons, particularly the ITS2, there is no publication on Mammalia utilizing these sequences. The major reason is that, for the most part, these sequences are not available. Only for a few rodents and for three apes [13-16] are the sequences for ITS1-5.8S–ITS2 directly available from GenBank. There is an ITS2 collection, the ITS Database III [17] that accumulates ITS2 sequences plus their secondary structures, but it contains no further correctly identified mammalian examples. Furthermore, although we now have an abundance of genome sequencing projects, there is one critical region of almost every eukaryote genome that is frequently omitted from the final genome assembly, the nuclear ribosomal RNA repeats. And, whereas most eukaryotes of all types have individual ITS1 and ITS2 regions of less than 500 nucleotides in length [12] these regions were known to be as much as 1 kb in rodents and apes. 

The present paper seeks to remedy this oversight by extracting the ITS sequences of mammals from the library of sequence fragments accumulated in GenBank, determining their RNA secondary structure as an aid to their alignment, and evaluating the phylogenetic information revealed by this alignment both at higher and lower taxonomic levels, as well as assessing its potential contribution to understanding RNA transcript processing.

## Results

### Sequences and Paralogs

We obtained the ITS1 and ITS2 sequences and derived the secondary structures for 33 species of placentals as well as the complete ITS1 and ITS2 sequences of one marsupial, *Monodelphis* (Table 1). In addition, the ITS1 sequence of *Macropus* is incomplete, and the ITS 2 of *Macropus* contains one short gap. For *Ornithorhynchus*, ITS1 is incomplete and in ITS2, helices I and II are complete, but not all of the remainder.

**Table 1:** Species and characteristics of mammals studied.

Genus and species of Mammalia		**ITS 1**	ITS1 length	helix #3	helix #4	5’arm on #4	#4 tip motif	helix#6arms			**ITS2**	ITS2 length	have insert	first5’ arm,III	2^nd^ 5’ arm,III
								5’	mid	3’					
**Euarchontoglires**								+	+	+					
*Rattus norvegicus*	rat		1062	+	+	**no**		+	+	+		757	+	**no**	**no**
*Mus musculus*	mouse		996	+	+	**no**		+	+	+		1088	+	+	**no**
*Cricetus cricetus*	hamster		1105	+	+	**no**		+	+	+		1123	+	+	+
*Cavia porcellus*	guinea pig		1708	+	+	**no**		+	+	+		1335	+	+	+
*Dipodomys ordi*	kangaroo rat		1674	+	+	**no**		+	+	+		636	stub	+	?
*Spermophila tridecemlineatus*	ground squirrel		1198	+	+	**no**		+	+	+		1121	+	+	+
*Ochotona princeps*	pika		1433	+	+	**no**		+	+	+		956	+	+	+
*Oryctolagus cuniculus*	rabbit		1592	+	+	**no**		+	+	+		1009	+	+	+
*Macaca mullata*	rhesus		1023	+	short	**no**		+	+	+		1166	+	+	+
*Nomascus leucogenys*	gibbon		1066	+	short	**no**		+	+	+		1130	+	+	+
*Pongo abelli*	orangutan		1079	+	short	**no**		+	+	+		1040	+	+	+
*Gorilla gorilla*	gorilla		992	+	short	**no**		+	+	+		1165	+	+	+
*Homo sapiens*	human		1074	+	short	**no**		+	+	+		1163	+	+	+
*Pan troglodytes*	chimpanzee		1078	+	short	**no**		+	+	+		1162	+	+	+
*Otolemur garnettii*	galago		1524	+	+	yes		+	+	+		1353	+	+	+
*Microcebus murinus*	mouse lemur		1091	**no**	+	yes		+	+	+		1314	+	+	+
*Tupaia belangeri*	tree shrew		1427	+	+	yes		**no**	**no**	+		1568	+	+	+
**Xenarthra**															
*Dasypus novemcinctus*	armadillo		1108	+	+	**no**		+	+	+		1090	+	+	+
*Choloepus hoffmanni*	sloth		1340	short	+	yes		+	+	+		1149	+	+	+
**Afrotheria**															
*Loxodonta africana*	elephant		1658	+	+	yes	+2	+	+	+		1351	+	+	+
*Procavia capensis*	hyrax		1678	+	+	yes	+2	+	+	+		1240+	+	+	+
*Echinops telfairi*	Mad.hedgehog		1766	+	+	yes	+2	+	+	+		1406	+	+	+
**Laurasiatheria**															
*Equus caballus*	horse		1427	+	+	yes	+1	+	+	+		1150	+	+	+
*Canis lupus*	dog		1862	+	+	yes	+1	+	+	+		1117	+	+	+
*Felis catus*	cat		1698	+	+	yes	+1	+	+	+		1727	+	+	+
*Sus scrofa*	pig		1467	+	+	yes	+1	+	+	+		1409	+	+	+
*Tursiops truncatus*	dolphin		1820	+	+	yes	+1	+	+	+		1328	+	+	+
*Bos taurus*	cow		1775+	+	+	yes	+1	+	+	+		1095	short	+	+
*Bubalus bubalis*	water buffalo		881+	+	+	NA	NA	+	+	+		981+	short	+	+
*Pteropus vampyrus*	bat		1398	**no**	+	yes	+1	+	+	+		1001+	+	+	+
*Myotis lucifugus*	bat		1240	**no**	+	yes	+1	+	+	**no**		790	bulge	+	+
*Erinaceus europaeus*	Eur. hedgehog		1524	+	+	**no**		+	+	+		954	stub	**no**	+
*Sorex araneus*	shrew		1299	+	short	**no**		+	+	+		925	**no**	+	+
**Marsupialia & Monotremata**															
*Macropus eugenii*	wallaby		2000+	NA	NA	NA	NA	NA				554+	**no**	**NA**	**no**		
*Monodelphis domestica*	opossum		3080	-	-	-	-	-				725	**no**	**no**	**no**
*Ornithorhychus anatinus*	platypus		NA	NA	NA	NA	NA	NA				NA	NA	NA	NA

Dimensions and characteristics of mammalian ITS1 and ITS2 regions. Where a sequence is as yet incomplete, the cited length is followed by a "+". Headings list subportions of the ITS1 or ITS2 secondary structure found in all species (+), unless stated otherwise (in Bold). For the motif near the tip of ITS1 helix #4, sequence similarity is indicated as type 1 or 2. NA = not available.

For all of these species except *Monodelphis* and the great apes, we obtained only one sequence, i.e., sequence homogenization is essentially complete. In *Monodelphis*, all sequence fragments match one or the other of two variants; this fits the report of Merry et al. [18] that there are ribosomal repeat regions on two different chromosomes in this species which might have diverged. 

The situation in the apes is quite different, for they are known to have multiple chromosome sites of rDNA repeats from in situ hybridization studies [19], all of which contributed to the pool of sequence fragments used to reconstruct the single ITS used here. *Homo* chromosome 21 and 22 ITS sequences differ at a number of positions, usually single nucleotides, from the chromosome 2 and 16 sequences; likewise there are scattered differences among overlapping fragments for *Pan* and for *Gorilla*. The sequence obtained for *Pongo* had two clear variants supported at numerous places. Thus the sequence version we arbitrarily selected to use for each of the great apes in the alignment combines nucleotides from two or more variants.

There is one quite different type of exception. In only this one case, *Pan*, was an additional clearly different and probably pseudogene sequence obtained for the entire ITS1- 5.8 - ITS2. This sequence, called *Pan* B in the alignment provided in the supporting data, has significant insertions/deletions, even in the 5.8S. 

**Figure 1:**
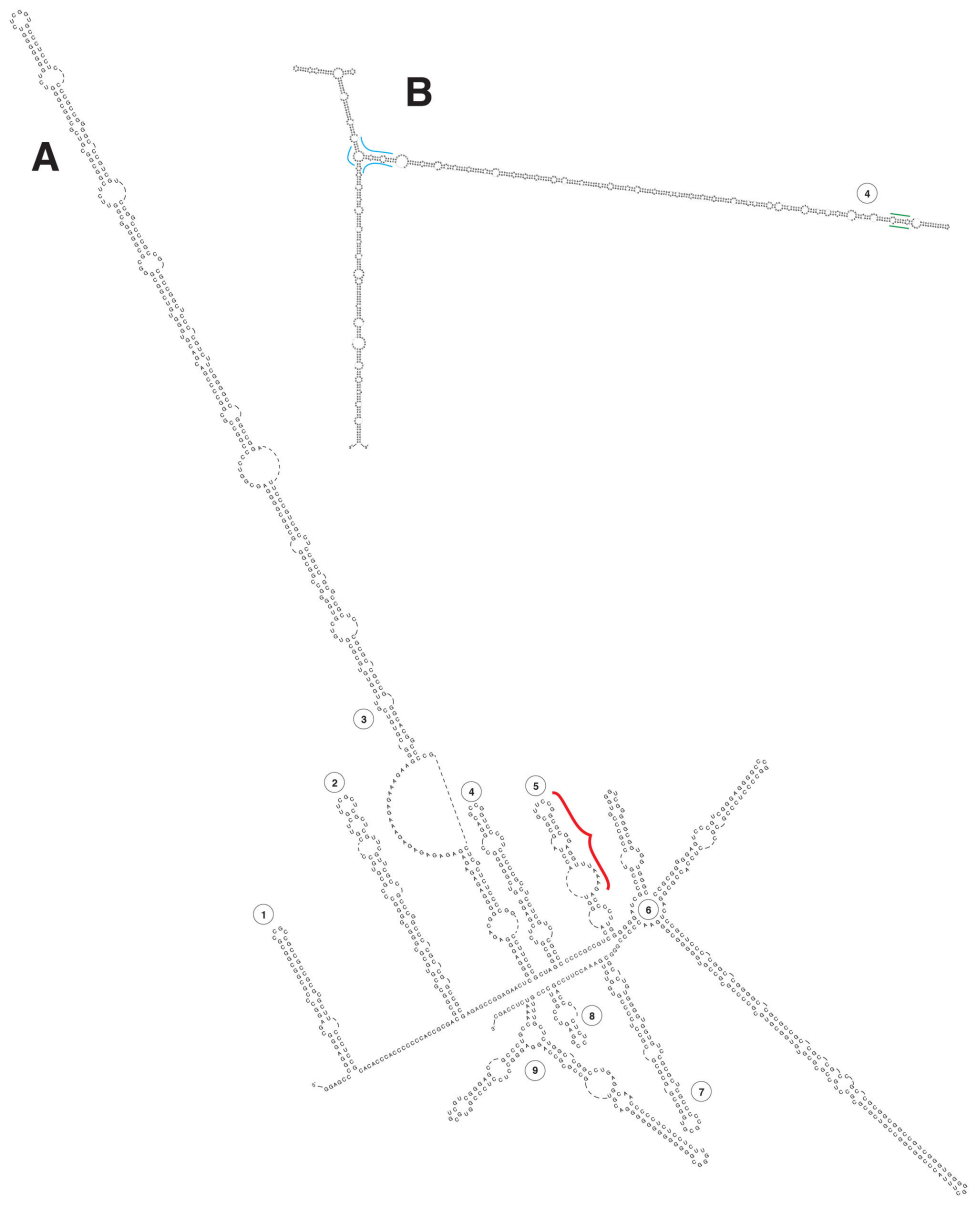
ITS1. A. Secondary structure of the ITS1 transcript of Homo. Hairpin loops are numbered in arabic numerals. Red bracket indicates position of the highly conserved motif. B. Insert, showing the much longer helix #4 of *Felis*, 849 nucleotides in length. Where the 5’ side arm joins this helix, blue marks show the position of the conserved nucleotides that delineate this branch, which is present only in those animals so marked in Table 1. Near the apex of this helix, green lines indicate the position of conserved sequence distinguishing Afrotheria from Laurasiatheria.

The mammalian ITS1 and IT2 sequences are unique in several aspects, but particularly in their length (Table 1). The length of ITS1 in marsupials, >3kb, is the longest yet known for eukaryotes. In the placentals, ITS1 ranges between 992 and 1862 nucleotides; the ITS2 range for all mammals is from 636 to1568, the latter possibly the record for eukaryote ITS2. The GC content and pairing stability is remarkably high in some places. For example, in ITS1, the helix that forms the third arm on helix #6 (Figure 1A) has a GC content of 80%; it has 122 nucleotides, making 55 pairings, 45 of which are CG/GC. Only 9 nucleotides in the stem are unpaired. Whether this high pairing stability has any function is unknown, but in times past, it might have presented a challenge to PCR.

### 5.8S Sequence

Except for the *Pan* pseudogene, the 5.8S sequence is identical among all the mammals for the central 155 of the ca. 160 nucleotides: 

5'-ACTCTTAGCGGTGGATCACTCGGCTCGTGCGTCGATGAAG AACGCAGCTAGCTGCGAGAATTAATGTGAATTGCAGGACACATT GATCATCGACACTTCGAACGCACTTGCGGCCCCGGGTTCCTCCCG GGGCTACGCCTGTCTGAGCGTCGCTT-3'

### Derivation of ITS Transcript Secondary Structure

**Figure 2:**
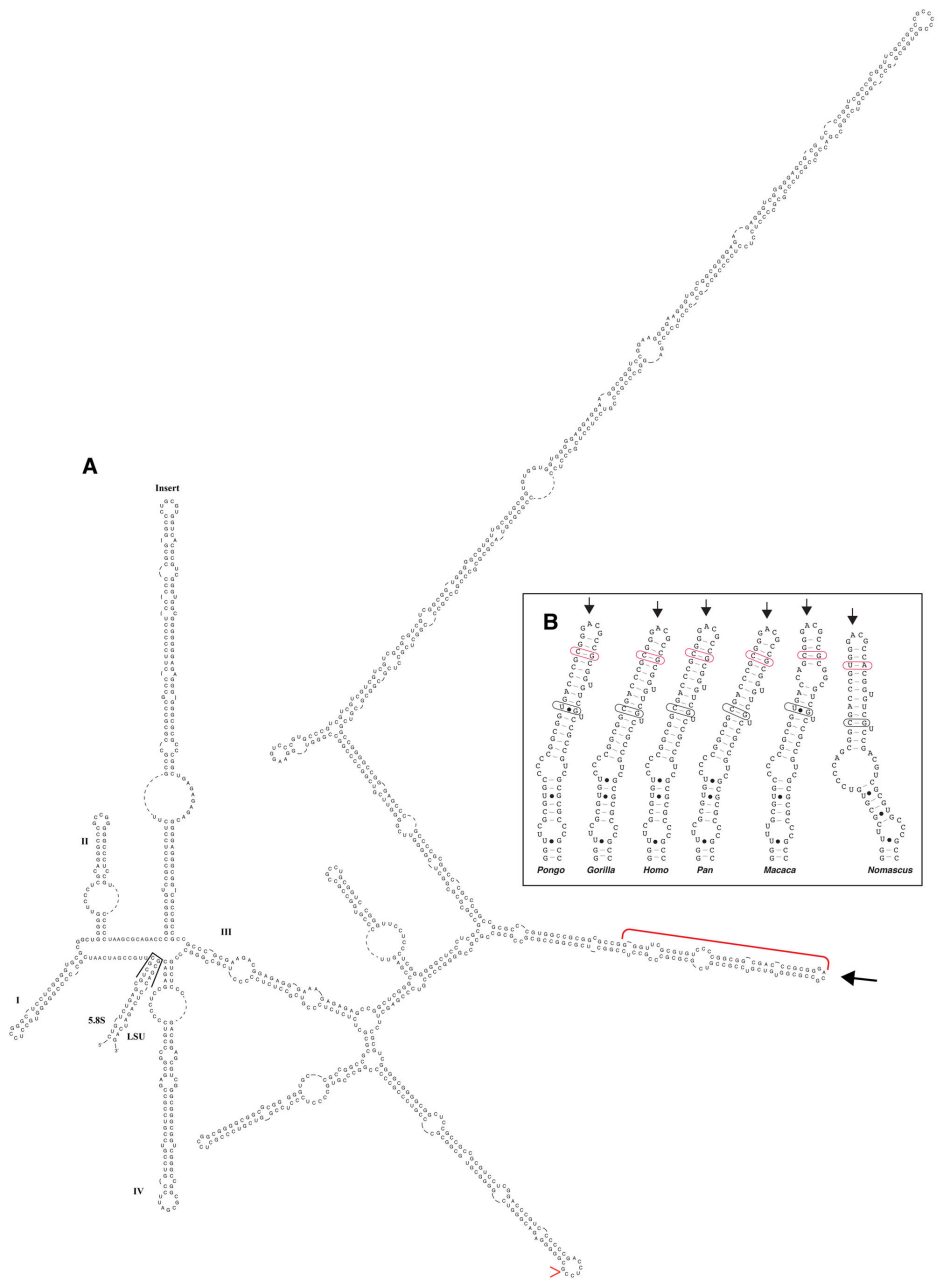
ITS2. A. Secondary structure of the ITS2 transcript of *Homo*. Hairpin loops are numbered in roman numerals, except for the helix between II and III, unique to mammals (see Table 1), labeled “Insert”. In addition, the 3’ near-terminal sequence of the 5.8S gene and the 5’ end of the LSU gene are shown as they pair, bringing together the ends of the ITS2. On helix III, a red bracket delineates the most highly conserved region of ITS2, characteristic of all eukaryotes; the large arrow locates the conserved terminal GACG/GAAG of this helix, common to all Eutheria examined except the rat. Red caret marks the site of *Alu* insertion (see text). B. Terminal 30 nucleotides on the 5’ side of ITS2 helix III of five apes, along with their pairings and the terminal loop. Red box indicates an example of a pairing position where a CBC (compensatory base change) is found; black box indicates one where a hemiCBC (one-sided base change) is found.

The *Homo sapiens* ITS1 and ITS2 foldings are shown in Figure 1 and Figure 2, and the alignments are presented (in TextEdit) as a NEXUS document in Text S1 and S2. In Table 1, the exceptions (insertions/deletions) to the major shared characteristics of the eutherian secondary structure of ITS1 and ITS2 are tabulated in bold for the species.

ITS transcript secondary structure has been extremely valuable in guiding alignment of sequences [11,20]. The first RNA transcript foldings of ITS1 and ITS2 for mouse and rat were proposed by Michot et al. in 1983 [13], and for *Homo* by Gonzalez et al. in 1990 [14]. After sequencing four new mouse ITS2 sequences, in 1999, Michot et al. [16] proposed secondary structures for rat, mouse and human ITS2, and in a further paper [15] for fish, amphibians and the chicken. The dearth of sequence from related organisms left the secondary structure uncertain.

Here, the criteria of “correct” folding structure are the presence of compensatory base changes (CBCs), where both nucleotides at a pairing position change between organisms, and hemiCBCs, where only the nucleotide on one side of a pairing changes [21]. These were found repeatedly in the more conserved regions, the bases of the helices and the terminal helix III of ITS2 (Figure 2B). Furthermore, the major indels of ITS1 and ITS2 are strikingly correlated with the secondary structure; each neatly excises one or two adjacent helices entirely. 

### ITS1 Transcript Secondary Structure

ITS1 has no recognized transcript secondary structure common to all eukaryotes. Placentalia ITS1 secondary structure is complex, yet conserved; we have merely numbered the hairpin loops almost universally present (Figure 1A). The *Monodelphis* ITS1 secondary structure, despite its great length, appears to be simpler than that of the Placentalia, but sequences from other marsupials are needed to prove the secondary structure.

For descriptive purposes, Placentalia ITS1 can be divided into two halves, of which the first half, helices #1 through #4, is by far the more variable in sequence and in helix length. Both bat species totally lack helix #3, as does *Microcebus*. The most phylogenetically interesting helix here is #4, relatively short in the apes and *Sorex*, but quite long (see Figure 1B and Table 1) in other mammals. Most of the longer versions of helix #4 have an arm on the 5’ side (position marked in blue in Figure 1B), but neither the rodents nor *Dasypus* nor *Erinaceus* and *Sorex* have this. A recognizably conserved pairing region near the tip of the long version of helix #4 (green marks in Figure 1B) clearly distinguishes the Afrotheria from the Laurasiatheria.

The overall sequence and structure of the second half of ITS1 is remarkably complex and rigidly conserved in the Placentalia. By contrast, the comparable region of *Monodelphis* and *Macropus* seems to have only three simple helices, bearing no sign of relationship to the eutherian secondary structure. *Ornithorhynchus*, whose sequence we have been unable to complete, appears also to be relatively simple. The only interesting variation in the complex Placentalia ITS1 3' secondary structure occurs in helix #6. With two exceptions, this helix shows the same trifurcation illustrated in Figure 1A for *Homo*. *Tupaia* lacks the first and second helices, retaining only one simple helix, corresponding to the third branch, by sequence. *Myotis*, on the other hand, lacks the third branch, retaining only the first two. The other bat represented in the data, *Pteropus*, has the trifurcated structure.

The most striking characteristic of the overall ITS1 is a centrally located 15 nucleotide sequence (Figure 1A, red bracket), identical in all Placentalia. This same motif (shown below) can be recognized in Marsupialia (*Monodelphis*, and *Macropus*), in *Ornithorhyncus*, and in birds (*Gallus* and *Taeniopygia*), both by its sequence similarity and by its identical position in the secondary structure.

Placentalia GCGGAGGTTTAAAGA YCCTCGGG
Marsupialia GCGGAGGTTTAAAGA T -- TCGG
*Ornithorhynchus* GCGGAGGTTTAAAGA TG -- CGGG
Aves GCGGMGGTTTAAAGA C ---TCGGG

We have found nothing even close to this 15 nucleotide sequence in the representation of Reptilia and Amphibia in GenBank, nor among the fish. For transcript processing, rat and human are known to have two/three internal cleavage sites in ITS1 [22]. The second cleavage site identified for mammals is at exactly the motif presented above. It is apparently common to all extant warm-blooded vertebrates.

### ITS2 Transcript Secondary Structure

As described by Michot et al. [23] and Peculis and Greer [24], the ends of the ITS2 are brought into close proximity by a very highly conserved 15 basepair sequence near the 3' end of 5.8S that pairs with an equally highly conserved sequence at the 5' end of LSU (Figure 2A, black bracket); these sequences are identical in all mammals.

The mammalian ITS2 retains the ITS2 structural hallmarks of other eukaryotes [11] and its helices are labeled accordingly (Figure 2A). Helix II displays the diagnostic pyrimidine-pyrimidine mismatch bulge near its base in all 33 Placentalia, the two marsupial and the monotreme species examined here. Helix IV is quite variable, sometimes absent in other eukaryotes; here it appears to be present but quite variable.

As expected from other eukaryotes, helix III carries the longest conserved sequence of the ITS2, on the 5' side of its terminus (Figure 2A, red bracket). Remarkably, even the loop nucleotides at the tip of helix III (large arrow, Figure 2A) are conserved in placentals, being GACG/GAAG in all except the rat, which is slightly truncated. Of the two 5' sidebranches of helix III, the second and longer is present in all Eutheria except the rat and mouse. It is also absent from marsupials. The first 5' arm is absent in *Erinaceus* and the rat (the most streamlined of the placental ITS2 here), as well as the marsupials.

What differs most strikingly in the eutherian ITS2 secondary structure, compared to other eukaryotes, is the appearance of an additional helix between helices II and III, which we label here “Insert”. It varies greatly in length among the different subgroups of Placentalia. The “insert” helix is totally absent only in *Sorex*, although it is truncated in several other placentals; this helix is not present in the marsupials, and we do not yet know about *Ornithorhynchus*. It is absent in birds and reptiles [25].

In other eukaryotes where hybridization is more easily studied, a correlation between ITS2 sequence evolution and gamete compatibility has been found [26]. The correlation held for all eukaryote sequences then available for which crossbreeding information was also known. The correlation related to the 30 nucleotide region on the 5' side of helix III, the most conserved region of ITS2. If this region differed in pairing by even a single CBC between two species, they showed no sign of interbreeding, not even gamete fusion. They were totally unable to produce a zygote. It is interesting to examine this region here. *Bos* and *Bubalus* are the two closest species represented; their 30 nucleotide regions near the tip of helix III are identical, though there are great differences in other areas of their ITS2 sequences. The two species can intercross to produce just the earliest stages of embryonic development. The only other closely related organisms here are the apes. With respect to *Homo*, *Nomascus* has a CBC (marked in red in Figure 2b). Both *Pongo* and *Macaca* have hemiCBCs (marked in black). The critical 30 nucleotides might suggest, as it proves to represent in other eukaryotes, a possible successful gamete interaction between *Homo, Pan* and *Gorilla* at least (see [27]); however the many differences in the remainder of ITS2 suggest there is little likelihood of any further developmental possibility. 

## Discussion

### Phylogenetic Applications

**Figure 3:**
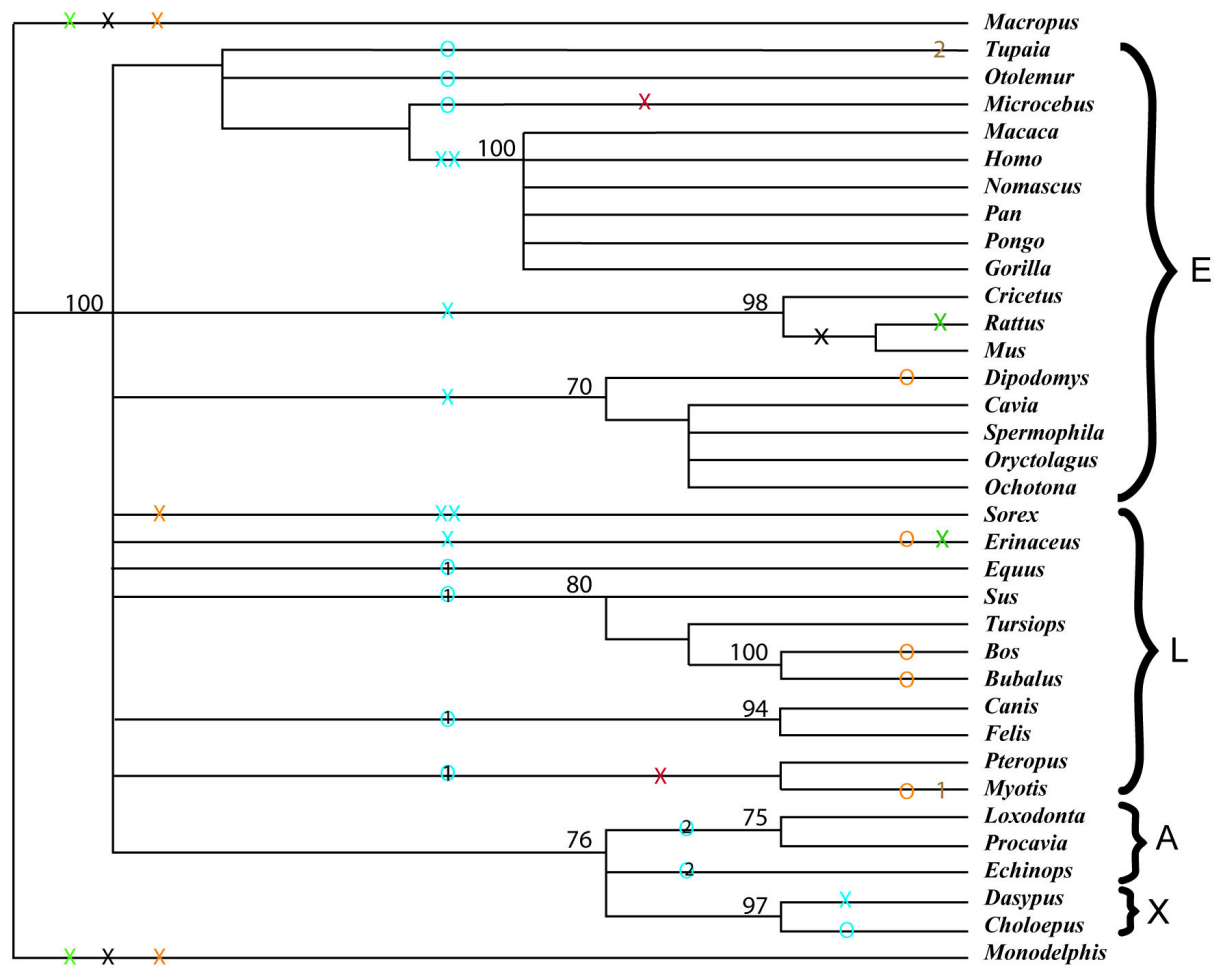
Evolutionary cladogram for mammals and marsupials. Analysis based on the most conserved 1,005 positions of 3,155 in the total ITS2 alignment (helices I and II plus conserved bases of branches: positions 810-940, 1121-1209, 2411-2469 plus conserved helix III positions 1641-1929), using the MacVector software; the tree represents the result of 1000 replications of the neighbor joining distance program using the model of Tamura-Nei; numerical values are bootstrap values. E, X, A and L indicate the four superorders of Mammalia. Maroon X = absence of helix #3 in ITS1. Blue X and XX respectively, = short or very short helix #4 on ITS1. Blue O without or with numeral 1 or 2 = presence of 5' arm on helix #4 of ITS1, showing no conserved affinity, affinity 1 or affinity 2. Brown 1 or 2 = number of subhelices in helix #6 of ITS1, where 3 are present in the remaining Mammalia. Gold X or O = absence, or great reduction, of "Insert" helix in ITS2. Green X = absence of first 5' branch helix on Helix III in ITS2. Black X = absence of second 5' branch helix on helix III of ITS2.

The ITS regions, even the more slowly evolving ITS2, are typically most useful for phylogenetic comparisons at the family level and below. However, a simple distance cladogram based on the conserved regions of ITS2 (Figure 3) shows the four superorders and provides a scaffold on which to map several aspects of secondary structure. The sequence and structure of ITS 1 helix #4 (blue symbols) support the Afrotheria and Laurasiatheria clades, although *Erinaceaus* and *Sorex* differ from the other examples of Laurasiatheria; these two species consistently appear basal in the Laurasiatheria in gene studies [2-5,7,28]. Here, both sequence comparisons and the structural indels found in ITS1 and ITS2 emphasize the basal position of these two insectivores of the Laurasiatheria. Furthermore, *Sorex* is the only placental to lack the “insert” sequence in ITS2 (gold X in Figure 3).

If lack of the "Insert" helix represents the ancestral state, as observed in marsupials, then the possibly more primitive state in Placentalia is found in *Sorex* (Table 1). Alternatively, and perhaps more likely, since there are five scattered instances where the “insert” helix is severely truncated, the “insert” helix may be truncated or lost with some frequency. Either way, this would be interesting to pursue among the Eulipotyphla. 

At lower taxonomic levels, there are several interesting synapomorphies of ITS secondary structure. The two bat species share the absence of helix 3 in ITS1 (maroon X), yet differ for presence/absence of the three arms on helix #6 of ITS1 (brown 1) and for the presence of a normal length "insert" in ITS2 (gold O), all of which should be useful for study of Chiroptera.

As with other eukaryotes, remaining questions of family, genus and species relationships should profit from ITS sequence comparisons, particularly ITS1, when the sequences become available. The inclusion of the *Bos* and *Bubulus* in the alignment shows where to expect variability versus divergence on the level of such closely related organisms. For them, primary sequence similarity is so obvious that secondary structure knowledge is unnecessary.

One final observations on these ITS sequences might attract those interested in mammalian phylogenetics. The two prosimian sequences, *Microcebus* and *Otolemur*, are quite different from the other primates as well as each other (Figure 3), perhaps even more than the latter differ from *Tupaia*, a member of a different order. Prosimians seem remarkably diverse, and will prove an interesting group to study further using ITS sequences.

### RNA Processing

ITS sequence and the alignments should help in the continuing effort to unravel just how RNA processing proceeds in the nucleolus, a longstanding focus of research [22,24]. For ITS2, the regions important to transcript processing are fairly well known and are present in all eukaryotes, including reptiles, birds and mammals (see discussion in Kupriyanova [25]). For ITS1, which has been much less studied, the discovery of a highly conserved site (Figure 1A, red bracket) common to birds and mammals should be a useful aid to recognizing the nucleotide positions important for cofactor binding for this spacer.

### Ancient *Homo sapiens* Subgroups

A third possible use for ITS arises from the peculiar situation in apes, where the rDNA repeats are found on multiple chromosomes. It is well known that *Alu* repeats are widespread in the genomes of apes [29]. In fact, we found a number of DNA fragments where ITS1 or ITS2 sequence suddenly became recognizable *Alu* sequence. One such site that might prove interesting is known to be present on chromosome 2 of *Homo* because the sequencing was done on sorted chromosomes. The transition from 5'-ITS2 to 3'-*Alu* is in the 3' arm of helix III, which is bifurcated. The break site lies in the terminal loop of the 5’ bifurcation (Figure 2A, red caret). Twenty-two different *Homo* ITS2 fragments had this identical alteration from ITS2 to *Alu* at exactly the same position (e.g. GenBank ID:AC097532); none were found for *Pan*. However this happened initially, it has been perpetuated in multiple copies, and since *Alu* repeats transpose into new positions with high frequency, such sites might serve as homolog markers, useful for tracing the lineages of ancient *Homo sapiens* subgroups.

## Conclusions

Mammalian ITS sequences are nearly the last major eukaryote group to be assessed for sequence and secondary structure. It is now clear that mammals have by far the longest ITS2 regions of all known eukaryotes, and yet retain the hallmark helices II and III. The ITS2 sequences of the Placentalia, with the sole exception of the insectivore *Sorex*, include a novel helix inserted between helices II and III. A further novelty appears in ITS1; the Mammalia incorporate a highly conserved sequence in mid-ITS1 which is unique to warm blooded animals, and perhaps serves as a processing site for the long RNA transcript. With the current ease of PCR and sequencing of >1kb targets, the stage is now set for the further use of ITS comparisons at intra-ordinal to species taxonomic levels where they have proven so useful with other eukaryotes.

## Materials and Methods

Although the ITS sequences of a few rodents and three apes are identified in GenBank, for all the species examined here (Table 1) a different approach was taken. Although an initial step of selectively removing rDNA from DNA preparations for genome projects is sometimes utilized, it is never 100% effective; there are literally thousands of ribosomal repeat genomic fragments in the genomic libraries, mostly not identified at all, but sometimes misidentified as putative portions of some protein gene. These can be recovered from the trace archives as well as the libraries of nucleotide collection, genomic survey sequences, and whole genome shotgun contigs, all of which were searched. Using BLAST, and starting with conserved sequences at the 3' end of SSU and the 5' end of LSU plus the 5.8S sequence, one can recover multiple overlapping fragments, gradually assembling the entire SSU to LSU region.

The identity of almost all nucleotide positions in all species here is supported by many sequenced DNA fragments; where fewer fragments were recovered for a subregion, a minimum of at least three such independently sequenced nucleotide fragments were required before the sequence was accepted.

In a few Placentalia (Table 1) we were unable to complete a junction with certainty. In ITS1, *Bos* and *Bubulus* are missing some sequence from the first half. In ITS2, *Bubalus, Pteropus* and *Procavia* each have a short gap. Thanks to the highly conserved regions representing bases of helices, sequences could be aligned by eye using MacVector software (Kodak, International Biotechnologies Inc., Newtown, CT). 

After alignment of conserved regions, potential secondary structures of ITS1 and ITS2 were derived by comparisons of the ten most stable foldings, as produced by Mfold [20] using the default settings. The structure common to all was revealed by the conserved basal pairings of helices and their compensatory nucleotide changes that support their validity. The cladogram in Figure 3 was derived as described, using the MacVector program.

## Supporting Information

Text S1 **ITS1 Nexus alignment file in TextEdit.**
(PDF)

Text S2 **ITS2 Nexus alignment file in TextEdit.**
(PDF)
