# Supplementary material for: Analysis of Mammalian rDNA Internal Transcribed Spacers
Source: PLoS One. 2013 Nov 19;8(11):e79122. doi: 10.1371/journal.pone.0079122 (PMC3834078; doi:10.1371/journal.pone.0079122)
Supplement: Text S1 — ITS1 Nexus alignment file in TextEdit. (PDF) [file pone.0079122.s001.pdf]

```
#NEXUS
begin data;
	dimensions ntax=34 nchar=3188;
		format
			gap=-
			datatype=DNA;
	matrix

Pongo
GGAAGGATCATTAACGGAG-----------------------CGAAGA----
GCGAGGCCCGCGGCGG---------CGCCGCCGCGGCGTCCTTCCTCGTCGGCCGGCCGGCCGC---------
GTTTCTCCCCGCTTCCCGCGGCGCGTGCGCGGGCGGGGCCC-----GTGCC---
GTTCGCGCGCACG------------------------
CGCGGGCGTGCGTGCGTTCGTCGCCCGGCCCCGCCGGCCGCG-------AGAGCC-GGAGAACC-----------
TCGGGAGGGAGA---GAGAGGGGGAGAGA-----GAGAGCGGTGT---------------
GTGTGTGCGCGCGCGCGTGTC--TCGGGGGCGGCCGGCG----
TGGCGGGCGGCGGGGAGCGGTCCCCGGCCGCGG-CCCC----GACGTGTGTGTCGGCGGGCGCGGGTG-------
CGGTCCTCGGCGGCGTCGCGGCGGGGT---
GGGGGGTGTCTC---------------------------------------------------------------
---------------------------------------------------------------------------
-----------------------------------GATGCCC---TCCCCGC---------------------
TGGGGC-CG--TCATCCC--GCCCTGTCCT--GCC---GGCTCGGCACAATCC---
CGCCTGCTCCAGTCGGGGCCA-GCTGGGTTCCC---
ATCGCTGCCACTGCCGCCATCGTTGCCTCTGCAACACCATGCCACCGGGCGCGGCCTGGCCCACTGGCTCTCT-
CCAGCCTTCCCA-CTA------------------------------GGATGTCTCAAGGATAG-
GGGGCCAGA------------------------------------------------------------------
---------------------------------------------------------------------------
---------------------------------------------------------------------------
---------------------------------------------------------------------------
---------------------------------------------------------------------------
---------------------------------------------------------------------------
---------------------------------------------------------------------------
---------------------------------------------------------------------------
---------------------------------------------------------------------------
---------------------------------------------------------------------------
---------------------------------------------------------------------------
---------------------------------------------------------------------------
---------------------------------------------------------------------------
---------------------------------------------------------------------------
----------------------------------------------------------------------
TGCCT-GTCCCCTCC--------------TCCTCGTCC-GCC---------CCCACAAT-
CCATGTACCTAG----CGCGTT-GTGGCGTGGAGGTTTAAGGACCCCTTGGGGG------GTC-
ACCCGTCCGCCC--ACGGG-TTGGGTGC---TGT-GAGC----CCAC------------GGGGAGTCCTT-----
TCGGGAGAGGCCC--------------------------------------------------------------
------------------------------------------ACCCC-TCCCTT----------GCCTCCCCC-
GC-----CGAC------TCCGTACC----CCCAGCCGG----------------------------
```

CTGGGACCGCGCCTCACTT----------------------------GCGTGTTG-CCACTGAT-GCC-
GGTGGGGG-------------------CTTTACTCAGTGGCT---
GTCATGCTGTCGCATGTGTGCCGCG--
---------------TGTGTGGTGTGTGCCCGGCGCCATG-------GGGGCAGGATTCCTC------
GGCCGCCCGTGGGGT----GGTGTCAGC---GTTTGCCC----------------
CCTGC------------------CCCGTGGTCGAC--AC-TGCC------TCCCT-GTGTT---------
GTGAAA----------CCT-TCC--AACCCCAC-------TCTGGAGT-------CTGGTCCC--
GTTG-------CTGTCT--GACTGGCC-
AGCCTGAGGTAACCCCT--
----------------------------CTGGAGGATGTGCTGT-GCC-AGG-AGGG---
CCTCCCGTT---------------GTTGGGAGCACC--------CTCGT-------------
GAAAATCGACCTT--GTATGACTTTTAG

Gorilla
GGAAGGATCATT-ACGGAG-----------------------CGAAGG----GCGAGGCC-
GCGGCGGTGG---------CGCCGCCGCGTGCTTCCCTCCC-----------------------------
CCCCACC--GACGCGGCGCGTGCGCGGGCGGGGCCC-----GTGCC---
GTTCGTTC------------------------------------GTTCGTTCGTTCGCTGCCCGGCCCCGCC-
GCCGCG-------AGAGCC-GGAGGAC------------TCGGGAGGGAGA----
CGGGGGGGAGAAGAGAAAGGAGGCC--TGTCC-------------GTGTGTGCG--------TGTCG--
TGGGGCCGGCCGGCCGCGCTGGTGAGCGGCGGCGAGGCCTCCCCGGCCGCGG-CCCCGAC-
GACGTGTGTGTCGGCGGGTGCGGGGG-------CGGTTCTCGGCGGCGTCACGGCGGGTT-----
TGGGGGCCTC---

---------------------------------GGTGCCCTCCTCCCCGC---------------------
CGGGGCCCG--TCGTCC--GGCCCCGCC--GCGCC---GGCCCCCCC-----------------
GTCGTCGGGGCCG-GCCGGGTTCCC---GTCGCCGCCGCCGCCGCCGTCGCCTCCGCC-----
GCGCCACCGGGACCGG----CCCCGCTCGCTCTCC-CCGGCCTTCCCG-
CTA------------------------------GGGCGTCTCGAGGGTCG-
GGGGCCGGA--

CGCCG-GTCCCCCCC--------------TCCTCGTCC-GCCCC-----
TCCCCGCCGTTCCAGGTACCTAG----CGCGTTC-CGGCGCGGAGGTTTAAAGACCCCTTGGGGG------ATC-

GCCCGTCCGCCC-CGTGGG-TCGGGGGC---GGT-GGGC----CCGC-----------GGGGGGGTCCC-----
GTCGGGAGGGGCCC---
--GGCCCC-TCCC---GC-------GCCTCC-
ACCGC-----GGAC------TCCGCCC---------CCCGG----------------------------
CCGGGGCCG---CGGCGGCC-
GTCGGGTGGGGG-------------------
CTTTACCCGGCGGCC--
---GTGCGCCCCGCGCCGTG-------
GGGGCGGGAACCCCC------GGGCGCCTGTGGGGC----GGTGTCAGC---
GCTCGCC-----------------CCCGC---------------------GTGGGCGGC--GCGCGCC------
TCCCC-GTGGT---------GTGAAA----------CCT-TCC--GACCCCTC-------TCCGGAGT-------
CCGGTCCC--GTTTG---TTGCTGTCC--GTCTGGCC-
GGCCTGAGGCAACCCCCCCCTCCTCC---
---------------------GTGGGGGGGGGGGGACGTGCCGC-GCC-AGG-AGGG---
CCTCCCGGT---------------GTCGGGAGCGCC--------CTCGC--------------
CAAATCGACCTC--GTACGACTCTTAG

Homo
GGAAGGATCATTAACGGAGC---------------------CCGGAGG----
GCGAGGCCCGCGGCGGCGCC------GCCGCCGCCGCGCGCTTCCCTCCGC------------------
ACACCCACCCCCCCACCGCGACGCGGCGCGTGCGCGGGCGGGGCCCGC---GTGCCC--GTTCGTTC--
GC------------------------------
TCGCTCGTTCGTTCGCCGCCCGGCCCCGCCGGCCGCG-------AGAGCC-GGAGAAC------------
TCGGGAGGGAGA----CGGGGGAGAGAGAGAGAGAGAGAGAAAGAGAAAGAAGG---
GCGTGTCGTTGGTGTGCGC-GTGTCG--TGGGGCCGG-------------
CGGGCGGCGGGGAGCGGTCCCCGGCCGCGG-CCCCGAC-GACGTGGGTGTCGGCGGGCGCGGGGG-------
CGGTTCTCGGCGGCGTCGCGGCGGGTCT---
GGGGGGGTCTC--

----------------------------------GGTGCCCTCCTCCCCGC---------------------
CGGGGCCCG--TCGTCC--GGCCCCGCC--GCGCC---GGCTCCCC------------------
GTCTTCGGGGCCG-GCCGGATTCCC---GTC---------GCCTCCGCCGCGCC-------
GCTCCGCGCCGCCGGGCACGG----CCCCGCTCGCTCTCC-CCGGCCTTCCCG-
CTA------------------------------GGGCGTCTCGAGGGTCG-
GGGGCCGGA--

CGCCG-GTCCCCTCCC--------CCGCCTCCTCGTCC-GCCCC-------CCCGCCGT-
CCAGGTACCTAG----CGCGTTC-CGGCGCGGAGGTTTAAAGACCCCTTGGGGGG-----ATC-
GCCCGTCCGCCC--GTGGG-TCGGGGGCGGTGGT-GGGC----CCGC-----------GGGGGAGTCCC-----
GTCGGGAGGGGCCC---
--GGCCCC-TCCC---GC-------GCCTCC-
ACCGC-----GGAC------TCCGCTC---------CCCGG----------------------------
CCGGGGCCGCGCCGCC-----------------------------GCCGCCGCCG-CGGCGGCC-
GTCGGGTGGGGG-------------------CTTTACCCGGCGGCC---
GTCGCGCGCCTGCC---
------------GCGCGTGTGGCGTGCGCCCCGCGCCGTG-------GGGGCGGGAACCCCC------
GGGCGCCTGTGGGGT----GGTGTCCGC---GCTCGCCC-----------------
CCGC---------------------GTGGGCGGC--GCGCGC------TCCCC-GTGGT----------
GTGAAA----------CCT-TCC--GACCCCTC-------TCCGGAGT-------CCGGTCCC--GTTT------
GCTGTCTC-GTCTGGCC-
GGCCTGAGGCAACCCCCTCTCCTCTT---
------------------GGGCGGGGGGGGGGGGGACGTGCCGC-GCC-
AGGAAGGGCCTCCTCCCGGTGCG----------TCGTCGGGAGCGCC--------CTCGC--------------
CAAATCGACCTC--GTACGACTCTTAG

PanA
GGAAGGATCATTAACGGAGC----------------------CGAAGGGGGGCGGAGGCC-
GCGGCGGC------------GCCGCCGCGCGCTTCCCTCCCCC-----------------------
CACCCCCCGCCACAACGCGGCGCGTGCGCGGGCGGGGCCCGT---GTGCC---
GTTCGTTC------------------------------------
GTTCGTTCGTTCGCTGCCCGGCCCCGCCGGCCGCG-------AGAGCC-GGAGGAC------------
TCGGGAGGGCGACG--TGGGGGGGAGAGC----GAGAGAGACG--GAAAGAAGGGG-GCGCG-
TGTTCGCGTGCGC-GTGTCG--CGGGGCCGGCGGGCC-----
GGTGGGCGGCGGGGAGCGGTCCCCGGCCGCGGG-CCCGACGGACGTGTGTGTCGGCGGGCGCGGGGG-------
CGGTTCTCGGCGGCGTCACGGCGGGTTTGGGGGGGGGGTCTC---------------------------------
----

GGTGCCCTCCTCCCCGC---------------------CGGGGCCCG--TCGTCC--
GGCCCCGCCGCGCGCC---GGCTCCCC-------------------GTCGTCGGGGCCGGGCCGGATTCCC---
GTC------GCCGCCTCCGCCGCG-----CGCCGCTCCGCGCC-CCGGGCACGG----CCCCGCTCGCTCTCC-
CCGGCCTTCCCG-CTA------------------------------GGGCGTCTCGAGGGTCG-
GGGGCCGGA--

CGCCG-GTCCCCGCC--------------TCCTCGTCC-GCCCC--CCCCCCCCGCCGT-
CCAGGTACCTAG----CGCGTTC-CGGCGCGGAGGTTTAAAGACCCCTTGGGGG------ATC-
GCCCGTCCGCCC--GCGGG-TCGGGGGCGGTGGT-GGGC----CCGC-----------GGGGGAGTCCC-----
GTCGGGAGGGGCCC---
--GGCCCC-TCCC---GC-------GCCTCC-
CCCAC-----GGAC------TCCGCCC---------CCCGG----------------------------
CCGGGGCCGCGCCGCC--------------------TCCGCCGGTGCCGCGGTCG-CGGCGGCC-
GTCGGGTGGGGG-------------------CTTTACCCGGCGGCC---GTCGCGCGC------
GCGTGCCGCGC---
GCGTGTGGCGTGCGCCCCGCGCCGTG-------GGGGCGGGAACCCCCC-----GGGCGCCTGTGGGGT----
GGTGTCCGC---GCTCGCC-----------------CCTGC---------------------GTGGGCGGC--
GCGCGCC------TCCCC-GTGGT---------GTGAAA----------CCT-TCC--GACCCCTC-------
TCCGGAGT-------CCGGTCCC--GTTT----TTGCTGTCTC--TCTGGCC-
GGCCTGAGGCAACCCCCTCTCCTCT--
-------------------------GGGGGGGGGGACGTGCCGC-GCC-AGG-AGGGCC---
TCCCGGTGTG---------TGTGTCGGGAGCGCC--------CTCGC--------------CAAATCGACCTC--
GTACGACTCTTAG

PanB
AGAAGGATCATTAATGGAG----------------------CAAAGAGC----AAGGCTT-----
GGTGCT----------GCCGCCGTGTCCTCCCTCTTCGGCTGA-------------CCGTGTTTCCCCC------
ACTGCGGTGC-TGCACGGGTGGGGCCC--------------
TTGCCATTA--
GTTCGCTGCTCAGCCCTGCTGGCCGTG-------AGAGCC-GGAGAAC------------
TCGGGAGGGAGA----TGGGGGAGAGGGAAG------------------------
GTGTGTGTGTGTGTGTGTGTGCATGTTG--
TGGGGCTGGCTGGCTGGCCTGGTGAGTGGCTGGGAGTGGTCCCTGGTTGTGG-CCCC----
GATGTGTGTGTCCGTGGGTGCGGAGG-------TGGTTCTCGGCGGCGTCACGGCGGGGTTGGT----
GGGTCTT--

------------------------------GGTGCCCTACTCCCTGC---------------------
TGGGGCCCG--TTGTCT--GGCCCCACCCC--GCT---GGCTCCC-------------------GTC---
AGGGCTT-GCCGGATTCCC---ACCGCCGCCGTTGCCTCCGCTGTG-----------------
CCACTGGGCCCGG-----CCCGCTTGCTTTCC-CTGGCCTTCCTG-
CTA------------------------------AGGTGTCTTGAGAGTCG-
GGTACTGGT--

TGCTG-GTTCCCCCC--------------TCCTCGTCT-GCCA------CCCCTGCTGT-
CCAGGTACCTAG----TGCGTTCATGGTACTGAGGTTTAAAGACCCCTTGGGGG------ATT-
GCCTGTCTGCCC--ATGGG-TTGGGGG---TGGT-GGGC----CCTCC-----------GGGGAGTCCC-----
GTCAGGAGGGCCT--
--GGCTCC-TCCC---AC-------GCCTCC-
ACCAC-----GGAC------TCCGCCCC---------ACGG----------------------------CCGGG-
CCGCACCGCAC---TCGCGTGC-------AGGTGCCGATGCTGCAGTCC-TGGCGGCC-
GTTGGGTGGGGG-------------------CTTTACCCGGCGGCC---
GTCGTGCAGTTGCGCGCGTGCCGTGC---
----------------CTGTGGTGTGTGCCTTGTGCTGTG-------GGGGTGGGAACCCCC------
GGGTGCCTGTGGGGT----GGTGTCTGC---GCTCAAC-----------------
CCTGT---------------------GTGGGCAGT--GTGCGCC------TCCAA-GTGGT---------
GTGAAA----------CCT-TCT--GACCCCTC-------CCCAGAGT-------CTGGTCCT--GTTT------
GCTGTCTC--ACTGGCT-
GGCCTGAGGCAACCCCCTCT---

---GGGGACCTG--GTACAACAGTTAG

Macaw
GGAAGGATCATTAACGGAG-----------------------AAAGAGC----GAAGCC--
GCGG------------CGCCGCCGCCGCGTCCTTCCTCGTCGGCTTGACCGTG---------------------
TCCCCCCTGCGGCGCGTGCGCGGGCGGGGCCCGT---GTGCC---GTTCGTT--
GAC--------------------------------------CGGGCG---
GCCCGGCCCCGCCGGCCGCG-------AGAGCC-GGAGAAC------------TCGGGAAGGGGG----CGA-
GAGAGAGAGAGAGACA--------GCGGGGACCCCGGGACC--------GCGCGCGTGTGTGCG-
CGGGGAGGGGGTGGGG-----------------------TCCCCGGCCGCGG-CCTC----
GACGTGTGTGTCGGCGGGCGCGGGGGGGAGGG-CGGTTCTCGGC---GTCACGG-----------
TGGGGGTCTC---

---------------------------------GGCGCCC---TCCCCGCCG------------------
CCGGGGCCCG--TCGTC---GTTCCCGTCCC--GCC---GGCTGCC-------------------GTC---
GGGGCCG-GCCGGGTTACCGCCC-----------GCCTCTGCCGCGCCGCGGCGC------------
CGGGCCCGG-----CCCGCTGGCTCTCCGCCGGTCTTCCCG-CCA------------------------------

GGGCGCCTCGAGAGTCGTGGGGCCGGAC---

--------GCTGGTCCCG-GTCCCCCCC--------------TCCTCGTCC-GCCCC-------CTCGCCGT-
CCAGGTACCTAG----CGCGTTC-CGGCGCGGAGGTTTAAAGACCCCTTGGGGGGT----GTC-
GCCCGTCCGCCC--GTGGG-TCGGGGGT--CGGC-GGGCGCGCCGGC-----------GGGGGAGTTCC-----
GTCGGGAGGGGCCC---
--GGACCCCTCCC---GTC------
GCCTCGCCCCGCAC---GGGC------TCCGCCC---------
CCTGG----------------------------CGGGGGCCGCGC-GCGC---GCGCGT---------
CGCCGCCGACGC-GC-GCCG-CGGCGGCC-GTCGGGTGGGGG-------------------
CTTTACCCGGCGGCC---
GTCGTCGTGCCGTCGTCCGCGT---
-----GCCGCGCGCGTGTCGTGTGCGTGCCCCGCGCCGTGT-----GGGGGCGGGAACCCCC------
GGGCGCCTGTGGGG-------TGTCCGC---GCTCGCCC------------------
CGTC--------------------GTGGGCGGC--GCGCGGGTC----TCCCC-GTGGAA--------
GTGAAA----------CCT-TCC--GACCCCTC-------TCCGGAGT-------CTGGTCCC--GTTATA----
CTTGTCTC--GCTGGCC-
GGCCTGAGGCAACCCCCGCTC--
--------------------------------GGGGCGTGCCGT-GCC-AGG-AGGG---
CCTCCCGGT---------------GTCGGGAGCGCC--------CTCGC--------------
CACATCGACCTC--GTACGACTCTTAG

Nomascus
GGAAGGATCATTAACGGGA------------------------CGGAG------
GAGGCTCGGCTCGGC-------TCGG-
CCGACCGCGTCCTTCCGAA-------------------------------------
ACCCCTGCGGCGCGTGAGCGGGCGGGGCCCGT--
GGTGCCCAAGTTCGTTCGTGCC------------------------
TTCCGAGGTTCGTTCGTTCGTCGCCCGGCCCCGCCGGCCGCG-------AGAGCC-GGAGAAC------------
TCGGGAGGGAGAGAG-CGGGGAGAGAGAGACGAGAGACGCGTGTGCGGGGGA--
CGGGACCGTGCGTGCGCGCGCGCGTGTCG------TCGGGGGCGGGCC----------
GGCCCGGAGCGG--------------CCCC----GACGCGTGTGTCGGCGGGCGCGGGGG-------

CGGATCTCGGCGGCGTCGCGGCGGGGT-----
CGGGGGTCTC---

---------------------------------GGTGCCC---TCCCCGC---------------------
CGGGGCCCG--TCGTCC--GGCCCCGCCC--GGCC---GGCTCCC-------------------GTC--
GGGGGCCG-TCCGGGTTCCCGCCGTCGTCGCC---GCCTCCGCCGCGCCGC--
GCCGCCCGGCCCGCTCGCTCGCTCGCTCGATCGCTCGCTCTCC-CCGGCCTTCCCG-
CTA------------------------------GGGCGTCTCGCGGGTCG-
GGGGCCGGA--

CGCCG-GCCCCCCCC--------------TCCTCGTCC-GCCCC------CCACGCCGT-
CCAGGTACCTAG----CGCGTCC-CGGCGCGGAGGTTTAAAGACCCCTTGGGGG------ATG-
GCCCGTCCGCCC--GTGGG-TCGGGGGC---GGT-GGGC----CCGC-----------GGGGGAGTTCCC----
GTCGGGAGGGGCCC---
--GGCCCC-TCCCC--GC-------GCCTCCCTC-
GC-----GGAC------TCCGGCCC-------CCCCGG----------------------------
CCGGGGCCGCGCCGCGC---TCGCGTGC-------CGCCGTCGACGCCGCGGTCG-CGGCGGCC-
GCCGGGTGGGG--------------------CTTTACCGGCGGCC---
GTCGTGCCGTCGCGCGTGC--
-----CGCGCGGGCGTGTCGTGT--GTGCCCGCGCCGTG-------GGGGCGGGAACCCCC-------
GGGCGCCTGTGGGGT----GGTGTCCGC---GCTCGCCCC---------------
CGCG---------------------TGTGGGCGGC--GCGCGCCTCC---TCCCC-GTGGT---------
GTGAAA----------CCT-TCC--GACCCCTC-------TCCGGAGT-------CCGGTCCC--GTTG------
CTCGTCT--GACTGGCC-
GGCCTGAGGCAACCCCCCCCTCC--
-----------------------------GGGGGGACGTGCCGC-GCC-AGG-AGGG---
CCTCCCGGTGC-------------GTCGGGGGCGCC--------CTCGC--------------
CAAATCGACCTC--GTACGACTCTTAG

Microcebus
GGAAGGATCATTAACGGGA-------------------------GAAGCCC-GCGCGGGGTTCG----------
CTCGCGTGG-GCC--GTCCTCTT--

CCGCGC-------------GCGGGGCCC----
GGCGCCGTGTCGGGCC--
CCCCACACTGCGCCTTTCCCGC---------------
C--

GGGAGCGGTCCGTGG---
TGCGGGAGCCGCGCGTGCGCGCGCGCGCCCCTCTCGCCGCGCCCGCGCCC-------------------------
----
GCCGGCCGCCGTCCGCGGCCGCCCCGCGCCCGCGGATCGCGCGGTCG----
CGGCGGGTCCCGTCCCGCGTGTGGCGTTCGCCGGAGGTTCCC---------------------------------
GGGGGG-------------------ACCCGGGGCTTCCGGGAC--------GCCCCGGGGGGCGGG---
TCCGCCACCGCCC---
GCCCCGTGGACGGGTTCCG--

----------------------------------CCGCCCCCCG-----
CCCCCGACGGTCG--
---------------------------CCCGC-----
CGTCCGTCTGTGGGGTGGTCGGGGGGTGGTGG---

---------------------------------GGTGACCTCGTCGACGCGGGCGGGTG-GCGC--
CGCCAGGACCCCCCGCGAGGACGCGGGTCGCCG--
-------------CGGAGCCGGGGCCGGCGACGCGGTGGCGGCGATGGG--TCGACGCGCCC-
GCCGTCTGCCCTTACCC--------GTCC-GCTCCC------CCCCCTGT-CCAGGTACCTAG----CGCGTTC-
CGGCGCGGAGGTTTAAAGACCCC-TGGGG-------GTC-GCCCGTCCGCCC-CGTGGG-TCGGGGGGC-CGGC-
GGGC---CCGGTGTCGTCCGGGTTGGGGAGGGTGGAG--
GTCGGTTGGTTGAGAGGGCCCGGCCTCG--
CTCT-------------------
GGCGAGTCCACCTGGGTTCCCCTCCTTTCCTCCTCCTCCGTCTCCTCCTCCCGCGACGGCCCGGAAC----
-CCCGCC---------CCCGGG----------------------------CCGGGGTTGCCGGGCGC--
CGCACGTGC---------------
GACGAGGGGTCGGCGGCGGCCCGCCGGGCTC----------------------TGGCCCCGGCG------
GTCGCGGTCGTCGTCGGTCCGTC--
------GCCGCGCGTCGTCGTCC-GGCGCCCCGTGCCGT------TGGGGGCGGGGACCCCC------
GGGCGCCTGTGGGG-------TGTCCGC---
GCGCGA---GCGC---
GTGCGCCCC-GTGT----------GAGAAA----------CCT-TCC--GACCTT-----------
CGGAGTT------CTGGTCCTT-GTC----------GTCTC-GACTGGCC-

GGCCTGAGGCGATCCTCCCCGCGCCTTC---
-----------------------GGCGAGGGGGGGAAGTGTCGC-GCC-AGG-GGGG---
CCTCCCTCC---------------GTGGGG----------GCC-CTCGC------------
CCGAATCAAATCTC--GTACGACTCTTAG

Otolemur
GGAAGGATCATTAACGCTGA----------------------CGGAGGCACCGCGAGCCGGGT-----------
CTCGGCCTCGCGCGCGCGCTTCCTTTCC------------------------
TTTCTCCACCTTCCCGTGCGGTG------CGGGCGGGGCC-----
GGTGCC---
GCGCCGGCCCCGCTGGCCGCC------AGCAGAGCGATCCCGCAGCGGGTC---
TCGGAA---
GGGAGGGGTGGGTGGTGCGTGCGTGCGTGC---GGAAGGAGGGGGTGTG--------------------
GCGCGGTCAGGCCCGCCTCCCCG-GGATCGGGCCGCTCCCGCT---TGGGGG-------TGG------------
TCACGGCGTCGAA---
GGGGGGGCCTCTCC---

-----------------------------------GTGCCCCGCCCCTGCCGGTCCCCCCT----------
CCGGGGCCCG------CCT-GGTCC--
CGGGCCG------------GCCGTC----------TGG-------------CCGTGCC-----
GCTGACGTCTTTCCTGCCGCCGCCGCCGCCGCCGCCGCCTTTCCCCTCCC--CTC-----------
GCCCT--------GGGCGTCTCGAGGGTCG-
GGGTGGCCGCCGCGCGCCGAGTCCCTCCGACCCCTCTCCCCACCCCGCCCTCACTGTCACCCC----
GTCTTTGCCGGTCCC---------------------------------
GGCCGACCTGCGGGGCAGGGCCCTTCGGC-GCATCT---CGGGCCGCGTGGCGGTGGC--
CGGCGGTGCCCTCGCGGAGCTTCC--------------------------------CCCGGA------------
GGGGGTGGCCCTGCCGTTGGGACCCGCCGCGTCCCGCCCCGCGCG----TCCCG--CCTCCC---ACC--
CTGGACGGATTCCGTTCCCCGCCTCCCTCCCTCGCCCGCCGCGCCC-----------------------------
----

TCACCCCCCACCTCGGCCGCCGGTC--
---TCC------------------------
TGTCGGCTGGGTCGGGTCGT---

GGTGGGTGGGGCGGGTTAGGCGGAGGGACGGGGGGAGGGGGGGTGCCGGGTCCCCGTC---GCGGGCGGGTA-
GCGC--CGTCGGGACCGTGTCCC------------
GTGGGCGGCCGGGG-----------------------------CTGGGGCCGGGGGGGCTGTGGCCGGTTC-
CGGGGGTTGGGTGGTTGGGTGCGGGGGGGTTCGCGCCCTGGCCCGGGCCTTTTCCCTTCCCCGTTC-
GCCC----------TCCTGT-CCAGGTACCTAG----CGCGTTC-CGGCGCGGAGGTTTAAAGACCCC-
TGGGGG------CTC-GCCCGACCGTCC-CGTGGG-TCGGGGGC---GTC-GGGC---CCGGCGTCGTT----
GTGGGGGGTTG-----TGCCGGGAGGGT-
CGG---
CCTCGTT--CCGGCTC--TCCC---GTCCC---
GCTCTCCCGC-GCGCTC-GGAC-----TCCCACGC---------

CCCGGTGC-------------------------CCGGGGTTGCGGCGGCG-----GCGCG-------------
CCCGGGCCGCGGTCGCCGGCGCCC-GTCGGGAGGGGGCTGACC------CCCGACCCCACCCCGACGGCC---
GTCGTGCCCGCGCGCTCGCGGCGCGTCCGCGCCCCCCCC------------------------------------
------CCGCG-----TCGCCGGGTTTGGGGAAAACACACTAGGCGCCTGTCGGGAACCCCT------GGGCG-
CTGTGGGGGC----TCGTCCGC---GCCC----------------------
TTCC--------------------------GGC----GCGCCGGAC-
GTCCCCCGTGTTTTTTTTTTTTTTAAA----------CC---CT--GACCTTTC----------
AGAGT-------TTGGTCTC--GTG----------GTCTG-GACTGGCC-
GGCCTGAGGCGGCCCCTCCCTGCC---
--------------------------GGGAGGGGTGAGTGTCGC-GCC-
AGTGGGGGCGCCCCCCTCCGCTTT----------GGCGGGGTGGTGGTGGTGCC-
CTCGCCTTACGGCAAAAAAAAAAAACAGCTCT-GTACGACTCTTAG

Tupaia
GGAAGGATCATTAACGGGA-------------------------AAGG-----CGAGCCGC----------
CGCCGTCGGCGCGCGCGCGCGTCT--
GAAAGTCTGGT-----------CGGGGCCCC---
GGTG---

GGCCGCC--
---------------------GGAGTTCCGTTCTGTTTCTC-------------
GGTGGGGAGATGGCGAGAGAGAAGGGTGGGAAGGGTGAC----GGTGGCC------
GGGTGGTTGTACGCCCGAGGAAGGAGGGGG---------------CGGTTCACGGCGCCGCTGGGAGAGGGA--
CCCC---

------------------------GTGTCCCCACCC-
TGCCGGTCCCCCCTCCTCC--
--------------------CGGGCCG------------
CCCGTCGCTGTCGCCGCCCCTCTCCGGACCTCT---------------------------
CCTCCCTCGTCGTTTTCCCTGCCTTC------------------------------------
GGGGTGGCTCGAGAGTCCTGGAAGGGTTGCGGGGGGGGGGGGAGGCGCACGGGCGCTTCCCTTCCCTCCTCGCAC
CCCCTTGGCCGCCAGCCCGCGGCGATGCCCTTCGGCGTCGGTCGTTGCTGCCGTTGGGTCCAGCCCGCGCG--
CGGGACGCCCGCGGC-GCCTCT---
CAGGAGGCGCCGGCGGCGACGGCAGCGACGATGGCGCGCCGAGGC------------------------------
---CTCA-----------------------GCCACGATCCGCGTCGCCTGCCGCCGTCGCCACCGCC--
TCCCG--CCGCTC---GCC--
CTGGGCCGGTCGACCGACCCTCGGTCGTGCCTGGGTCGTCCTCCTCGAT--------------------------
----

TCCCCCGCCGCCGGTCGTGTCCCGCCTGGCCCTCGGGGGCCACGGTGTGGGGGGGGCTGCGTTCCCCCCACTCCC
CCCTCGTCCGGCCGCGCTCCTCTCCCTCCCTC----GCTC--TGAGGTTGGGGGTGGGAGGTCGG-
TGGTCCGGGTCGCCGGGCGGGTG--

CGGTCGGTCGGTCCGCGTGGTCTCGGTGGCGGCCCCGTGCG-GTC---ACGGGCGAGCA-GCGC--CGC--

GGACGGTCCCGTAGTCTGGCGGTGCTCCTCCGGTGGGGCGG----------------
CCGGCCGCCGTCGGGTCGCTCGTCGTGATGTCTGGCGATCGGGGG---
TCGGGGAGTGCGGGGGAGGACGCCGGCTTCTCCCCCCCACCCTTCCTCCTCCCGTCC-GCCC---------
CCCCTGT-CCAGGTACCTAG----CGCGTTC-CGGCGCGGAGGTTTAAAGACCCC-
AGG--

----------------GGGTCTGG--------TCCATCCCCCCC--
CCCGGGG---------------------------
CCGGGGTTGTCGAGCGCGTGTCGCGTGCC---------------GCGCCCGTCG-CGGCGACC-
GCCGGGAAGGGG---------------------CTGCCCGGCGGCC---
GTCGCGGGGCGCGC---
----------GTGTCCTGCGCGCGGCTCCCTCGTGCCTTCGTTGGGGGGGGTGGAGGCCCCC------
GGGCGCCTGTGGGGG-----TCGTCCCGG--GCTCGCCCCG---------------
CTGG-----------------CGGGGTTTGAGGCCCCC-----GGGTGCGCCCCCCTGTTT------
TGGTGAAA----------C---TCCT-AGCGGCC---------TCGGCCG--------CGGTCTC-
GGTTTT--------GTCC---GCTGGCC-
GGTCTGAGGCGACCCCCTTCCCCGCTCC---
------------------GGCGGGGGTGGAGGGAGATGTACCGT-
GCCCCTGGA-----------------------------GGCGGGGGGCTCC---------GGCCCCCCAC-----
CCTTCTCAAAACCTC--GTACGACTCTTAG

Rat
GGAAGGATCATTAACGG---------------------------AGAA-GGCCGAGGGGGGT--
CGTGCCCG----TCCCC-
TCTTGGGCCTGTGTGAGTGTTCCTCCTTC--

-----------------------------------TCGCC-
GGGAGGC--------------------------------------
GCGTCCCCGGGTGGGTCCCCGTGTCCGGCGTCCTGGCGTCGTGGCGCGTGCCGTGGCCGGCCGGCGGAGGGGGTT
TCGGGACAGGTGTGC----------------------------------
GGTGGC----------------------------AGGGTG---TCGGGTCT-GGTCCGCCGC-GGGACCT--
CCTCC--

GTTTTCTCGCTCTTCC--------------
CTCGACGCCTCCGTGCGCCCGCCTCCGCCTCCGCCGCTGCC-----GTCGGCAGC--
GAGGGCGTTCTGCCTCTTTCCCGA-CCGGC---TCCGTGATCTCGTGTGCACC----
GGGGGTGGTACGTGATCTCTCCGGGCGGTGGCCGGGACGC---------------------------
GCTCGCTCTCCTCGA--------------------
GGCGCCTGCCGCTCC--

TCCCCCGCCGCGGCTCTCCGGC---
---------------------------ACTGACGGTG---------
CCGGTGGCTCCGCC---

GCGTGCGGTGGGGGGGGG---
TGTGTGTGCCGGTGCCGCGGGTGGCTCGCGCGCCCCGCCGCGTCCTTCTGGGCCTCC--GTGTCC-
GCCCC-------GCTCCTGT-CCGGGTACCT-GCTCTCGCGTTC-CGGCGCGGAGGTTTAAAGACCC--
CGGGGGG-----GTC-GCCCTACCGTCCC-CGGGG-TCGGGGGGG-CGGTGGGGC----CCGT------------
AGGGAC---------
GTCGGTCGTCCGCGGGAGGGCT---
---------------------------------------TCCCGGTCCCTCCCCGCGTCCGGCG---
CTCCCTC--------AGAC------
TCAGCGGCGCACCCCTCT---
-----------------------------------GCC-GCCGGCGC------------------------
TTCCCGAGGCGGCG---
GT---
-------------------------------------TCGGGGGGGCGTGTCGCGAGCCCCCTCT---
GGGCGCCCGAGGGGTTC--CCGGCCCGA---GCGCCT--GGCTTGTCCCGCC-----CGTT-----
GGCGGGCCGCCGCCGGGTGCCGGGTT-CGTGGC------GTCCC-GTGTC-----CCGTGTGTCCCG---
CCTTTCCG-TCTCCGACCCTGTCTTTTTTTTTTTATTTTCTTACGTGTG---CCGTTTC--------
GTTTCGCGCTGGCC-
GGCCTGAGGCGGAGCCCCCTCGCCGTCCGTCGGGC-------------------------------------
CCCTCCCATCCCGCGCAGGAGGGGCGGGCGGGGGCGCTTGTTGCGGTCGTC-AGCA----CCCCGTGT-
GCGGAG---------------------------TTCCGCTCACACCTCAGA-----------TAACC----
GTACGACTCTTAG

Mouse
GGAAGGATCATTAACGG--------------------------
GAGACTGTGGAGGAGCGGCGGCGTGGCCCGCTCTCCCCGTCTT------
GTGTGTGTCC---

-------------------------TCGCC-GGGAGGC----------------------------------
GCGTGCGTCCC-----GGGTCCC---GTCGCCC-GCGTGTGGAG------------------------
CGAGGTGTCTGGAGTGAGGTGAGAGAA--
---------------GGGGTGGGTGGGGTCG-GTCTGGGTCCGTC-T-GGGACCG--
CCTCC--

GATTTCCCCTCCCCCT--------------CCCCTCTCCCTCGTCCGGCTCTGACCT--
CGCCACCCTACCGCGGCGGCGGCTGCTCGCGGGCGTCTTGCCTCTTTCCCGT-
CCGGCTCTTCCGTGTC--------TACGA----GGGGCGGTACGTCGTTACGGGTTTTGACCCGT-
CCCGGGGGC-GTTCG-
GTCGTCGGGGCGCGCGCTTTGCTCTCCCGGCACCCA---------------------------------------
----

TCCCCGCCGCGGCTC--
--------------------------TGGCTTTTCTA---------
CGTTGGCTGGGGCGG--

TTGTCGCGTGTGGGGGGA-----TGTGAGTGTCGC--GTGTGGCTCGCCCGTCCCGAT-GCCACGCTTTTCT-
GGCCTCGC-GTGTCC-TCCCC-------GCTCCTGTCCCGGGTACCTAGCTGTCGCGTTC-
CGGCGCGGAGGTTTAAAGACCC--CGGGGGG-----GTC-GCCCTGCCGCCCC-CAGGG-TCGGGGGG--
CGGTGGGGC----CCGT------------AGGGAA---------
GTCGGTCGTTCG---

GGCGGCTCTCCCTC--------AGAC------TCCA----
TGACCCTCCTCCCCCC---
---------------------------GCT-GCCGCCG-------------------------
TTCCCGAGGCGGCG---
GTCGTGT--
-------------------------------------GGGGGGGTGGATGTCTGGAGCCCCCT-C---
GGGCGCCCGTGGGG--------GCCCGACCCGCGCCGCCGGCTTGCCCGATTT---CCGC-------
GGGTCGGTCCTGTCGGTGCCGGT---CGTGGGT------TCCC-GTGTCGTTCCC---GTGTTTT-------
TCC-GCTCCC-GACCCTTTTTTTTTCCTCCCCCC--CACACGTGTC---TCGTTTC--------GTTCCT-
GCTGGCC-GGCCTGAGGCTACCCCTCGGTCCATCTGTTCTCC--------------
TCTCTCTCC-----------------------
GGGGAGAGGAGGGCGGTGGTCGTTGGGGGACTGTGCCGTCGTC-AGCA-----CCCGT------
GAG--------------------TTC--------GCTCACA------------CCCGAAATACCG---
ATACGACTCTTAG

Hamster
GGAAGGATCATTAACGAA----------------------
AAAGAGAACGGCCCCGGCGGGGGGCGTGTCTCT--------GTGCC-----
GCTTCTTCTT---

-------------------------TTGCC-
GGGAGGC-------------------------------------GCGTTCC-----
GGGTCCTGCTGTGCTC------------------------------------CGGAGGGTGGAG-
GAGGTGGGTCGTGGT-----------------------------
GGTGGCGGTGGTGGTGGTGGCGGCGGTGGCGTGCCTCTGCTCGGACGC-
GGTCCGTCCTCGGGACCG---

TGTGTTCGCCGGAGCCCGGAGGCTTGCTCCCTTCCGTCCATCCATCCAGCC-----
ATCCGCCCCGCCCTGCCCGCCCGCCGATTGCTCCTTCTG-AAGCCGCTAAAGCCCGTGGGCGTC---
CCTCTTTCCCGT-CCGGC-CTCGTTCGTCTTCACCGTTACA----GGGGCTGGTTCGC-
GTTCGTTCGCCATTAGTT-
GGGTGGGGAAGGAGCAGAGGTGGCTCGCCCGCTGTTGCGTTCGGGC---------------------------
----

TCCTGCCGCCGCTGCCGCCGCCTCGCCTTACCGCCGTGGCTACGGCTTTCTCG--------------------
--ATGCG-----
CCGGAGCCGGTGTCGTGTGGTTTGGTT--

GAGTGGCGCGCGGTGTCGGGTGTCACGTCACGTCGCGGCGGGACCCGTCTCTCTCCCGCCTCATCCCCTTCTGGC
TTCGGAGCGTCC-GGCCCC------GACCCTGT-CCGGGTACCTGGTTATCGCGTTC-
CGGCGCGTAGGTTTAAAGACCC--CGGGGGG-----GTC-GCCCTTGCGTCCCCGCGGG-TCGGGGGGG-CGGA-
GGGC----CTGT------------GGGGAA---------
GCCGCCCGT--
--GTGGCGG--
CTCCCTC--------GGAC------
TCCCGTTCCTTCCG---
----------------------------------CGG-ACGA-----------------------
GGCCTTTCCCGGGTCTC----
GCCGC--
---------------------------------------GGTC--TGGGTCGGGAGCCCCCTT---
GGGCGCCCGTGGGGC-----TCGCCCCTC--GTCGCCC-GGCTTGC----------CCGG-------
TGC--------GTCGGT---GGTCTC---GGG-------CCCC-GTGTCGTTCCGCGTGTGTTTTT-
TTTCCTCCCG-TCCC-GACCCTCTTC-----TTGAGAGTTT-----GTGTCGTCTTCTGGTTTT-----
GTCTC--GCTGGCC-GGCCTGAGGCGAATCCCCCCCTTATTCCGTCCTCCCCGTCCCTCCTGTTGTGGG-
TTGT----------TCCTTGGGAGAGGAGGTGTGGGGGTCGGTGATATGGGGGAACCGTGCCGCCGTC-AGC---
TTCCCAGTTGGGTCGAG------------------TTTTCGT--CTCTTCCCAC------------

GTTCGAAACGTTG---ATACGACTCTTAG

Cavia
GGAAGGATCATTAACGGACGATGACC----------------
CGGGCGACCCGAACGGATGGACGGGCGGGACGGACGAA-CCGGGGAAGGCGGGCCGGGAGCGGC--CAAGG-
GGCCGGCTCTCTCTCTGAGCCCTGGCCTTTCCTGCCGGTCGGTCGGTCGGTCGTTCG------------------
--------ATTGGGATCGCTT-----------------------------
GACGAGTCTCCTGTGTTCCTCCTCGAGAAGCC--GGGAGGCCCCGTC-CTCG-
CCCGCGTCGTTGTCGTGTC--
GCGTCGCGTCGCGCCGCGCGTGTGGTGCGTTGCGGTACGGGTCCCGGGTTCCGGGACCCGGCCCGGTCCGTGCC
AGGCTTGGGGGTTGGTGGCGGCTCCGGCGATCGCTCCCTCTGCGCCCGCCGGCTCCGCCCCCCCGCCCGTTCC--
----
TGCCCCCCCCCCATGTCGTGTGGCACCCCGCGCTAGCGA------------------------------------
-----------------------------GCGAG-CTTGGC-------
GCCTCGTGTGTGTCGCGCGGTTGGGGGGTTGGGGGTGGCGGTGGGGCGGGCGGGTGCGTGCGCGAGCATCGCCGG
GGGTCAGC---------
GCCCCCTCTCTGCCCGGTTTCGGGCCTGGCCCCGGTTCCCCCCGCCCCGCCCTCGCTACCGCCGCGCGCGTGCCG
GCCGGCCGTGTGGGGGGGTTCTCTTCACGCGACGACAGGCTT-CCCG--
CTCAGGGCGGTGT-------------------
CGGGCGAGAGCTGAGCGAGCCAGCGAGTGGAGCGGGCGGGAGTGTCGGTGACCCG---
CGTGTGCGCCTCGGGTGGGCGTGGCGTG------------
CGAAGGGCGTGCGGGCCGGTGTCTGTCGGTCGCGCCGCTCTCCCCCT-
GACCCTCC---

TCCCCGCCGCCTCTGGCGCCGTGTCC---
---------------------------GCTG--------------
GGTCGGCGTCGTGTCGGGGCGCGCGTGGGAGGACTTGGGGGCAGGCGGGAGGGCGGGCGGGGTCGGC--------
----

TGGCCCGGCCGCCCGTCTCTTCCTCCGGGGCCGCCGTGGCCGCGCTCCCGCTCCCGCTTCTGCACGTGTGCGTTC
GCTCCGTG-------CTTCCGCGC-GTCT-GCCC--------TCCCTC---TCAGGTACCTAG----CGCGTCC-
CGGCGCGGAGGTTTAAAGACCCCTTGGGGGGGGTG-GTC-ACCCCGCCGGCCTT-GGGG-TCTGGC----CGG-
CGGGG----TCGCGCT---------
GGGGGCTCCTGGGGGCCCGTGTCGGGGGTTTGGGCGGGAAGGACTTTGGACGTGAGAAGTCGGCACGTCGGGGGC
GTTGG--------
TCCCCGATGCCGCTGTGCTCTCTTCTCCGCGCCGGTCCTCCCGCCTCCCCGCCCCTCCCCCTGCCCCCACGCGTG
T--------GTACTCTT--TCCCCGCCCGCCTCTCCTCGG----------------------
TTTGGGCCGGGGCGCC------------------------

GCGCGCCGCTGACGTCTCCGGGCGTCCCGGCCGGGTTCGGGGG--------------------
TATACCCGGCCGGTCGTC--
---------GGGGC-CGTGTTGGCGTTGGGCGCGGCCCTCCCTTTCCGTTGAGGGG---
GAAGGTGGTGGGGATCCCCCCCCCCGGGCGCCTGTGGGGTC-----GGTCCTTCC-
GTCCCCGTGCGCTCGTCCGCT--CCGC------TGCGGGCCGTGGCGCCGGGCTC-GGTGGGTTGCCGAAGA--
GCCCC---GTCTCAGCGCGTGATAGACGCGCTCT-CGTGGCCCCTAACCC----------TCGGAGT------
TTCTGGT-----GTCGTTCTGTTCTTTCT-TGCT-GGCC-
GGCCCGAGGCGACCCCGTCTG---
-------------------CCCCGTGCGGGCGTGGAAGTGTCG-CGCC-
AGCGTCGGGGGTGCTTGTGGGGAGGTGGAA----
CGTTCCTCTCCCCCTCGCACGTCTGAAAAAAGAAAAAAAGCGAAACGCTCAC--GT-----------

Diplo
GGAAGGATCATTAACGGAAAGGGCACGGACTCGCGCGCGCGCGCGTGCGGGGCGAGCTCGGCGCGAGGGTGGC
CCTCCGCGATCCGCCGGGGTTGCCGGCGCCGCGGTCCCTCGGGGCTCCTCCTCGTCGTCGTCGCCGTCTTTCCCG
CGTCGCCGTCGCCGTCCGTCCCCGTGTGGCCGCTCTCGCACGTCGGGGCCGGCCTGGGAGGAGGGGCCCGCCCGT
TCCCGGGGCGGCCGTGCCGGTGAGGACCTTTCCCCCGTCCCGGTGTTTCCCGCGGCCGCGGACGGGTGAGTTGGC
GAAGGGGGCGCGAGAGAGGAGGAAGGAACGGTAAGAGAGGCCGGCTGGGGTCGCGTGGCCGGCGTCGGCCCGGTG
GGTGTGGCCGTCTGCGGTGCCGTCCCCGCGGCGCCCGTGCTGCGGGCGGGCGAGCCGGTGCCCGGGTTAAGGCAT
GTTGCGGGTGGGTCGTGTCGCCCGCCGGCTGCGGGGGTTAGGGTAGCCGCGGTGGGTCCGGGTTCCGGGCCCGGG
TGGCCGGGAGCGTCTGGGC-
GCGTGCCCTCTCTCCCGCTGGCGCCGCGCGGGCCCGCGCGCCCTCCCCGCCGCGGTGTCGCCCCCCCCCGCTGCC
TCCGCGGACGTCCTTGTCCAGATCACCCGCTTCCCCCTGCCCCCGCTCCGCCGACCCCGCCCGCGTCCGGGCCCG
CGTGGCGCATCGCATTCCCCAGCCGCTGGGGGCCGCCGCCGCGTCCGCTTTTCCCCGTCGGCCCGCCTCCGCCGT
CTCTGCGCTCGCTCCCCGCGTCCCCGCGCTCCTCTCCCGTTCTGCGCCACCGCGGGGCACCGCGCCTCGGGCCCC
TGCCTCGCCTCGCCTGTGCCGCCCTCGTGGCGGTCCCCTTCCCCGCTGGCTTTCCCG------------------
----
CGCTGGGCGCCTCGCGCGTCGGTCGGGGCGGGCTCGGCCTCGCCCGTGTGCGGCCGCCGTCCTCCCCCCCACCTC
TCGGTCCGCCTTCGCCTTCCCCTCCGCCCCCCGGTCTGGGGCCGGC----------------------------
----

TCCCCGCGGTGGCACCTTACCC--
----------------------------CGCCTCTGGC-------
GGGGTGGCCCCCGCTACCGGGCTTGCCGCGTCCCGTGGCTCGGTT-----------------------------
----

-----------CCCCCCCCCGCCCCCGTGTGGTTGGGGGGGCGG------------
CGGCGGGTGGGGCTGGCGGTGGTGCGGCTCGGGGGGTGGGGAGGGTGTCCGGCGGCCGCCTCCCCGGCGTCGCTG
CCCCCCCCGCCTCGCTCGTCC-GCCC--------TCTCGTGT-CCAGGTACCTGG-TATCGCGTTC-
CGGCGCGGAGGTTTAAAGACCCATCGGGG-------GTC-GCCCGTCCGTCC-AAGGGG-TCGGGG----CGG-

CGGGC----CCGT------------GGGGAG---------
GCCGGTCC---

CCGGTCTCTCCC----------AGAC------
TCCGCCCCGTCCC---------------------------------
GGGTCGGGGC---------------------------
GGCCCGCGCTCGC---
TCG--
----------------CGGGC-----GGGCCGCTTCTCCCGCAGCCCGGGCTTCGGGGGTGGGCGCCCCC-----
GGGGCGCCTGTGGGGTCGGGGGCGTCCGA---GCGCCCGCCCGCCGCGCC---CCCCGCGCT---------
GGCGCGGCCGCGGC---GCCGGGCGTCCCC----GTCCCC-GTGTCC---CGTGTG-------------------
TCC--GACCCCCTCC--------------------GTGTCTCGTTCTCGTTCT-----GTCTCTGCT-GGCC-
GGCCCGAGGCGACCCGACCC---
-------GCCCGCGT-----GCGGGACCGGGAAAACAGCGCCG-CGCC-
AGCCGC--GTGTGCCCCG--CGTCGGG--
CCACGTCCCGA---TTC--GT-----------

Sperm
GGAAGGATCATTAACGGTT--------------------
GCGCGAGGAGGGGAGATCCGGGGCGCGCCCTCTCC-------------------
TTCTCTTCC--

--------------------------GCGTGAGAGAGTTCCCGTGG---
CCGGGAGGGCTCCCGGGGGGGGACGGCGGTGGGCCC---------------------------------------
----

-
AGTCGGT--

---------------------------GGCCGCCGCCGCCCGCGAAAC-CCCGCGGT-
GTTTCCTCTGTACGTCGGCTT-CTCGCTA------------------------------------
GGACGGTTCCAGCGTGCCGCCTCCCGCGTGGGGTGCGGGGCGGAAGATCCGCCGCCCGCTCGCCGTTTCCGACGC
CGAGCCGGTCGCGGTCGGGGCGCGACCCGCGGCGCAGTCTCTCGAGGCGCCCGTGTGGGTGCGTGGCGCGCGCGC

GGCGGTGCGTGCCGTCGTGGTCGTCGTCGGGCGCACGCGGCCCGCGCGGGGGAGCC-------------------
---------------------------------------CTCCG----------GGGTGCCCCCCGC-
GCGAGGTGGTCGGGTCCCGTCGGCGGGCCAGGTGGCCTCGCCGCCGCCGCCTCCGCCACCACCACC---------
----

--GCCGCCTCCTGACG-
GCCCGCTCTGGACGGTGTTCCGCCGTCGCGGGTGGGTAGCGCTGCGGTCCTCGCGTCCGGCCGGGGCCGGGGTCG
GCGCCGGTTCCCGGCGCTTGGCGGCGGGACGCCCCTCCCCGCGGCCCGAGTGCGCTCGTCCCGTCCCC-----
--CCCTC---CCAGGTACCTAG----CGCGTTC-CGGCGCGGAGGTTTAAAGACCCT-CAGGGGC-----GTC-
GCCCGTCCCCCCTTGGTGTGTCGGGGGA----GACGGGC----CGGT------------GGGGA----------
GCCGCGG--
AAGG--
GCCCGGCCCGACCTCCGCTCCCCC--------AGGC------
TCCGCCGCCCGCCCC--
--------------------------------CCCCG--GTCGGGGTGCGTGCCGCGT----
CCCGCCGCTGGCGGCCG-
CCGGGAGGGGGTGC---
CCGGCGGTCCCTTAGCGGCGGGCGTGTGTGCCGCTCCGCGCCGTCGGTGGTGG--
TGGGGCTGGTGGGAACCCCC------GGGCGCCTGTGGGGCCC----GTTCCGT---GCGCCCG---
GCCGCGC-------CCCGG-------TGCGG---TCCTGCGGC---GCCGGGCGA---------GCCCC---
GTCGTGG------AAAAA----------CCCCTC---GACCGCCACTGCGTTTC------TG---
TTCTGGTA--------------CTCTTGCGTGCTTGGCC-
GGCAGAGAGGCAAGCTCTCTCTCTCTCGCCCCCGTCTTCCCCGCCCC-----------
GTGCCGCCCGTGCGGCGGGGTTGGGGGGGGCTGTGGGGTGGGGGGGAAGAGCCGGTGCCG-CGCC-
AGGACCAGCGGGACGAGGG--
CCCCTCGCGGCCCTCCTTCCGAAACCTC--AT-----------

Ocho
GGAAGGATCATTAACGAGGAC-----------------CGCCCGCCCGCGTCGTGGCGTGTGGC-
GTGTCGGCGACC---

TGCGCCCGGGGGGGAAGGAGGAGCCGTCTCTCTCGGTGCCGGCGGGCG---CGGGGATCTTACC---
GGGGACGTCGGGGCGGTGAGCCGCGCGGCGGCGGCGCTGGGCGGTGGCGGTGGCGGTT-----------------
----

GCCCCCGTTCCCCGCCTC---

----------------GGGGTGG-CGTCG-----------
GGGGGCCTGGCCCCTCCTCCCCGCGGT--
-----------GCCTCCTC--------
GGGGGCGCCGCGTTGGGGGGAGGTTCCCCCGCCGCGCCTCGGGGTTGGGGCTCGGGGGGGACTGGCAGCGTTGGC
CGGCCGGCCGGTCGGTCGTCGTGCGTCCCTCG--CTTCCCCTCTTCCCC--------------
TCCCCGCCCTCGGCCGGGGGTGCTCTCCTCTCTGGCCCCCCCGCACCTCCCGTGTGTCTCGTTGTTGGCCTGCCG
GGGGGAGCGCGAGGCGGGCCGCGTGTGTCGCGCGTCGAAGGGAGCGCGAGGTGGTGGTCGTGCCGTGCCTGTGGG
GCCGTGGCCGGCGGGCC--

TGGCTTGCCGAGGCGTTGGTGGTGGCGGCGGCGGCGTGCCGTCTCCGGGGCTGTGTGCCGTGCC-----------
----
TCCGTCGGCTCCGGCGTGCCCGTCTCGAACCTCCGGGCC------------------------------------
-------------------------CCCCTTCCGT--------
GGTCCGGGGGCGTGGTGGCCGAGCCCGGG--

GGAGGCTTCTGGCGTCCTCAGCCCCGTGTGGGGCTCCTCGCCCCCCTTCCCAGCCCTTGCCTCGCCTTGCTGTGC
CGCGGGCCCACGGCGGCCCCCGCCTCCCCCTACCGCCGGCCTCGGCCACGTGTGTCCTCG-------TCCTGG-
CCTCTCCTCTG----GCT--------TCTCTTGCT-CCAGGTACCTAG----CGCGTTC-
CGGCGCGGAGGTTTAAAGACCCC-CGGGGCC-----TAC-GCCCTTCCACCC--TGGGG-TCGGGG----TGG-
TGGGC----CCGT------------GGGGAG--------
TTGGGGCTTGGGGGGGGTTC---

AGCCTCGGCTGTCCGCCCTTGCCGCTTCTCCTCCT--------GG------
CTCCGCTCCCTTGCCCTGGCCTGTTGTCCCTCCTCCTCCCTCCACCTCGCCCGCCTCTCCCCTCACCCCGTCCCG
ACCG-GTCGGGGGGCGTGCGCCTTCCCCCCCCACCC----GTCGGCGTTGGTCTCGTC--
GTGGTGGTTCCGGCCACCCGTGCGGGGGCAGCGCCGGTGGGAGGGAGGGCTGTGGCGTCGCGTTCCCCCGCGCCG
TGCC------
CGGGGAGGGGGTGGGTGGCCGGCGGTGGGTCGGTGGTGGTCGGGGTTGGGTGGCGGCGGGGCTTCGGGGGCGGGA
GCCCCCT-----GGGCGCCTGTGGGGTG-----CGTCTGC---
GCGT--CAGA-
CGCGCCCC-GTGTGTC----------AAACCCA----------TCC--
GGCCCCGCCAGTGT----------------CTGGTT----GTCGTTTCG-----AGTCTGCC-GGCC-
GGCCTGAGGCAACCCCCTTCCCCTCACC---
--------------------GGGTCGGGGACGGCGCCGTGCC-TCGC--
AGCAAC---
GTTCAAGAAACTC--GT-----------

Orycht
GGAAGGATCATTAACGA-GAC---------------------CGGGCTTGGCCGG----CTGGCCGTGCCC-
TGCCTG---
ATTCCG---

TGC--
-----------CCGGGGGGGAAGGA-GAGCCCAGAGAAGGGAGTGTTCCTCCCCCCC-CCTCCTCT--
GGCGGGCGGTGTCGAGGGTGGCTCGGGGAGTCGCCGGGGATTGTGGCGAGGAGCGTGCGGT-----
GTCGCGGACCGGGTTGCGTGCGTCGGGGGGTCCGGACCCTCCCCCCCTCCTGCGCGTGCTCGGTGGTGACCGGGG
TCTGGCCGCCCTCTGGCGGTGGAGGGCCACGGGCGTCCTCCCTGGAGGAGAGGCGAGGAGGGGTGAGGCCGAGAG
GCGTGTGGCGTGTGTGGTGTGTGTGTGTGGTGGTGGGAAGAGGCGCTGGGGGGGGGGCGGTGTGCCGCTGGCGGT
GGTGACGGGGTGTGGGGCGTGTGC------------------------------TGGTC------
GCGCGTCTCCCCCTCGCCGTCTCGGGCTCCGCTCCCTTGGCTCCTCTCGCCCCCTCCCCCCACTCCATGCCGCTG

CCACCTCCCCCCCGCCCCTCGTCTTGTCTCTCCATCCCGGGGCCCGTCATCCCTCTCTCCGTTGGAGGCCCGGCC
GCCCCGGAGCCACCTGGGCGCGGAGCAGGGAGGAGGAGGTTGTCCCCTCCCACGCACGCCTCGGGGCCGTCGC-
CGTCCTCCCGCCTTTCCCTCTCCCCCTACCCCTTCTG----------
CCGCACTCCTCTCACCCCCAACTTCGTGGCGTCCCGAGAGGGCCTCGAGGTCTGTGTGTGCTTGTCGTTGGCGAT
GGGGGTTGTTGGGGGTGGA--

CGAGGGGGCGTGTGCCGTCGCTGCCGCGTGGCCGTGGCGGTCCGGGTTGGCCGGTGGGGTGCGTCTGGGTGGTGC
GTGTGCGCCTCTTGCCCTCCTCTTCCGTCTCCGGCG-----
TCCGTCTGAGCTCCCCGGT--
----------------------------CCCCTGT--------
GGCCGGGGGGCGGGCGTCGGGCGGGGGGGG---

TGTCTGGAGGTGGCCTCGGTGCCACCTCCCGTGCCTCATTCGGTTTCCCCGCCGTCGTCCTCGCGCGCGGCTTGC
ACCGCTTCCCCCTCCCCCCAACTTCTCCC--GCCT----------CTCCT--CCAGGTACCTAG----
CGCGTTC-CGGCGCGGAGGTTTAAAGACCCC-CGGGGGT----GTACCGCCTGTCCGCCTC--GGGG-
TCGGGG----CGG-CGGGC----CTGT------------GGGGAG--------
TCGGGCGGGTCCCG---
---TCCCGTCCCGTCCGT-
CCTCCCCCT-------GA------
CTCCGCTCCTCCTGCCCGGCCGAGG--------------------------
CGGGGGCGCTCGCTC-----------------GCGCGCCGCGCAGCCTCTCGCGGCGGCTGCC-
GTCGGGAAAGGGGCGC---

CTGACCCGGCGGTGGTGTCGTGCCGCGAGAGGGGTGCGCGCGGGGGCGTCGCGTCCTTGTCCCGTGCCGTGTCGG
GGGGGGGCGGGAGCCCCCTT----GGGCGCCTGTGGGGT------CGTCTGC---GTGCTCGCCC-
CTCGCCGCGGC------------GGCGGCGGCGGCGGAGGC-GGCTGGC----GCCGGG-CGCGCCCC-
GCGTC-----------GAAACCCA-------------CCGACCCCTCCCAGAGT-CTTTC---------
ATTGTGTCC---------------AGTCTACT-GGCC-
TGCCCGAGGCAACCCCTTCTCTTCT--
-----------------------GTGGGTAGGGGATGGTGCTG-TGCC-AGG--------
CTTTCCCTT---------------------------------------GACGGGAAAG-
AGCCAAAAAAAACCTC--GT-----------

Dasypus
GGAAGGATCATTAAC--GAGA---------------------GGAGCGCCGGCG----AGGCGCC----
CGAGCCTGCGTTCGGGCCCGGCCGAGCGTCCC-----------------------------
GTTTTTTGCCCGTGCGGCG------CGGGC-
GGGCCGGCGCGGTGGCC--
--------GGCCCGCTGGCCGCGA--------GAGAGAGCGAGAAGCC---CGC---

```
GTGGCGCGCGAGGTTGC-----------GGGCGAGGGCCGAGGTTG-
TGGAGGTAGGCGAGGAGGGGCCGGGGTCGGGCGCGCCTCCGCTCGCGGTTGCCGCGGGTCGTGGGGGGGCGG---
-------------------------------------------------------------------------
CTCCCGTCGTGTCCCCCGC--------------------------------------------------------
---------------------------------------------------------------------------
------------------------------TGTGG------------
TGGGGGGGCTTTCGGCGGGACCGCTGTGGTTCCCCACGCCGCCGCCGC---------------------------
-------------------------------------------------------
TGAGGGGGGGCGCCGCCCGGCCGCCCGGCCGCCCGCCTGCCTGCCTGCCGCCGTGCCCCCCGCCCGCCCTCCGTG
TGCCGCGC--------------------------------------GAGGCGGTGCGAGAGTTCC-
CGTGTCTCCGTGTCCCCGCCTATGCCGCCGAGTCCTCGGGTGCCCGGTCCGCCGCGGCCGCCGCCGCTGCCGTCG
AGCCCGCCCCGCCCGCCTGGCTGCTGTTCGCCCCCGGGCCGGGTCTCCGGGCGACC-------------------
---------------------------------------------------------------------------
---------------------------------------------------------------------------
---------------------------------------------------------------------------
---------------------------------------------------------------------------
---------------------------------------------------------------------------
---------------------------------------------------------------------------
---------------------------------------------------------------------------
---------------
GGGTGCGC-------------------------------------------------------------------
---------------------------------------------------------------------------
-------------------------------------------------------
CCGGTCGTCCGTTTCCCGGCCCCTTGGGGCGTCGCGGCCGGCGCCGCGGCCCC----------------------
-------------------------------------------
GCGCCCTCGCGGGGCGTCCGCTGGCGGCACGGGCCCCCCGGGCCCGCGTCGAGGC-------------------
----------------------------------------------GGTGGCC-GGCTTCTCTCGCCC-
GCC----------GCCCTGT-CCAGGTACCTAG----CGCGTCC-CGGCGCGGAGGTTTAAAGACCTT-
TGGGG-------GTC-GCCCGTCCGCCC-GCGGGG-TCGGGG----CGGGCGGA----CCGT------------
GGGGAGCGG------
CGGGGCCGGCC--------------------------------------------------------
CTC-----------------------------------------------------GGCCGGGCCT---
CCGCTCTCCCCC--------AGAC------TCCGCCTTCCTGGG-----
CGGGC-------------------------
CCGGGGCGCCGAG--------------------------------------------------------------
---------------------------------------------------------------------------
----------------------------------------------------------------
GTGCTCCGTGTCC-GCCGGGGAGG--CGGGAACCCCC------GGGCGCCTGTGGGGTT--GTGTTGCCGC---
GCCCGGCCGGCC------------CCTG-----------TGGCCGGCCGAGCCGGCCGAC------------
GCGCCCC-GACTGTCAT-------------------CCCC--TCCT-GACCCCC--------
TCTGGAGT-------CTGGT--CTCGT-----------GTCTTT--CTGGCC-
GGCCAGAGGCACCCCTCCCCTTC----------------------------------------------------
-----------------------GCGGGGGAGGGAGAGCGCCGTCGCC-
AGGTTTGGATTTG-----------------------------------
CTGCAAGTCCAAAACC------------AAAAAAACTCT-GTACGACTCTTAG
```

Choleops
GGAAGGATCATTAACGCGAGA----------------------
GAGCGCCCCCGCCGCGGGTCCCCCGAGCTCGATCGGGTGTCCCCTACGGGTCGCGTGACCCC-------------
------------------GTCCCGCGCGGCGC------------
GGTCGGCCGGCC---
------------GCCGGCCGCGC--------GAGAGAGAAGCGAGGATAAG-----
GCGGCTGCCGAGCC---

CCTCTCCATGCCGC--
GAGGCGGCGCTAGAGGTCGACGTGTCCGCCACGCCCCA-------------------------------------
-----------CGCGCCGGTCT-CCCCGGTAGCCGCGG------------------
CCGTCCGCCCGCTGGTGGCATTCCCCCCGGC-GCGTCT---
CGGGAAGGGAGGGCGGTGTCGGTGGCACCCGCGGAGCCCTCCGGGGTCCTGCC----------------------
ACCCG-------GGGTGGGGCTCGGAGCCTCGGCTCACGCGGGGC----TGCCGCCGCTCCGCCTCC--
TCCCG--CCGCCC---GCC--
CTGGGTCGGGTCTCCCGGGCGATCCGCCTGTCGCCGACCCTCTCCGTCCGTCCCGTCCCGTCCCCCACCGCCGGC
GTCGCTTTCCTTCCCGGCTCCGCACCCCGGGCTTCGCCGCCATCCGGCCGCCACCGA------------------
----
----------------TCCCTGCCTC-
GCCTTGCGCCACCGCGTCCCTTCCGTTGACCGCCGCCCCTCCCT-------------------------------
--GGGTGTG-----
GGGGAGGGAGGGGTCCCTGGGTC--GCGGTT-
GCGGGGAAGGCAGGGGCGTGTGTGGGG--

CCGGGTGAGAGCGGCGGGGCCCCGTGGGCGCGGGGTCCGGTGGGGGGGTCTGTGGCGACCAGGTGCGTGGGGGGG
GGTCCCGGGCGCGGGGCGGGGGGTGGCGCCGGCGGCGACGGTGCTCGGTTGTCCCCCGGCC--ACGGGTGAGCA-
GCC--GCCGCGGTGCTT-GTGTCCCC-GCGGG--------TGGCGGCG-----------------
GCGTTCGGCCGGGGCCGGAGT--
-----GGGGGCGGTGGCCGGGCACCTCTCGCCC-GCC----------ACCCTGT-CCGGGTACCTAG----
CGCGTTC-CGGCGCGGAGGTTTAAAGACCCT-AGGGG-------GTC-GCCCGTCCGCCCCTTGGGG-
TCGGGG----CGGGCGGGA----CCGC------------GGGGAG--------
TCGGGGGCAAGCCCCT---

CCGGGTCTGCCCTTTCTTCCCCC--------AGAC------TCCACCCCCCTGC---
CCTCAGGC--------------------------
TGGGACG--CCAC-
GTCGCGCCGCGCCTCC---------------
GTTCCGCTAGGCCGGCGGGCC--
---------------GCGCGCCGTTCGCGGCGTGCGCTCCCGGTTGTTGAGGGGTTTGGGGGGGGGG--

TGCGCACCCCC------GGGCGCCTGTGGTGG---CGGTGTC--A---GCCCCCGCGCCCGCCCTC-------
CCG---------------GGGGCGAG-TGCGTCGGGGGAAGGCCGCCAC---------------------
CGGT-----------CCA-TCCC-GACCCTGC--------TCCGAGT-------CTGTTT-
TTCGTTTCCT--------------CTGGAC-
GGCCAGAGGCGCCCCTCCTCCG---
---------------------------TGGGGGGAACGTGCCGTTGCC-
AGGTGG--------------------------------------TTGAAGAG--CAATTC-AA--------
AAAAAAAAGAACGC---GTACGACTCTTAG

Echinops
GGAAGGATCATTAACGGAGG--------------------CGCGAGCCGGGAGGCGCCTGCGCGCG--
CGCGCGTGGTGCGTTCTCGGCCGCGAGCCC---------------------------------
GACCCCGAGCCGGCGGCG----------------CTCC--
GATG---
------CGCCGCGAGCCG----GAGAGAACGATAGA-----CGCGC-
GCGGTGCGTC---
TGGGGTGGGGCGCGGGCCGGTGCGGCCCGGCGGCGGTGACGGTCCGGGGCCCCGTCCCCGGTTCCC---------
----
CGGGCGGGCGGG---

----------------------CCGCC----
GTCCGCTCCGCTCCGGGGGCCGGTCGCGGCTCCC---

TCGCCGCTGCCGTCGTCGCCGTGGCCGCCGCGCCTC-------------------------
TTCTCGCCGCGAGGCGAAGCGGGGGGGCGGAGTCGCGTCCCCCGCGCCCTCTGGCGCCGGCGCCC----------
----
GGCCACCCGCGTGTTTCCCGTCGGGGCTTCGCCGGCCCCCAGCGGT-GCGTCT---CGGGAGGGG--
GCGGCGCGGCGTGGCGGTTTGTGGGGTGGGGGGCGC-----------------------------
GAGC-----------------------GCGTCCCTCTCCTCCTCCGCCGCGTCTTGCGTCGCCGCC--TCCCGC-
CCTCCT---GTC--
CTGGGCTCGCGTGGTGTGCTGGTTGCGGGGTGGCCTCCCCGCCCCTTGTCGGGTCCCGCCTCCGTCCTCGGCCTC
GTCGCCCTGGCTTGTGCCCGGCCGTCCGTCCTTCCGCCTCTCCTCCCCTCCCGTGTGCTTTTCCCCCCCCACCAC
CACGCTTCCCACTGTCCCCCTCCGCCGATCTCTCTCCCCGTGGCACCCTTCCGGGTCCCGGTGACCGGCTCCTCA
CGCGCCTGCTCGTCCCGAAGCCCCGCGTGGTCCCTCTGTCGC-
TCCCGCCGGCCCCT---
------------------------------CAGC-----
GGCGCCGGCGGGGGGGGCCTTGGGCCGCGCGGGTAGCGAGTTAGGTGTGGTGGGCCGGTTGCGGGGGACCTCTCG
GGTCTGGGGTGAAGGCGGTCGCGGCGACGGTTGGGGGCCTCTGAGGTGGGGGGAGGCCGCGCGGTCTGGGATGGA
AGATGGCGCGCGGGTCCGGGCGTGGCCGGGTCGCGCCGGGTGCGACGG--
GTGTCCCGGCGACGGCAGCGTTGGGGCCCGCGGGGTTTGGGGGGCATCCCTCCGCCTTCTCCTCGCTGTC--
ACGGGCAGGC-CGC---
GCTGCGGCGGGGTCTCGGTGTCTCCTCGGCGGGGGACGTGGGCTGCG-------------
ACTGAGGTTCGGCGTCAGGAG--
--CCCGGTGGCCGTCTGCCGGCCTCCTCTGCTCTGCC----------ATCCTGT-CCAGGTACCTAG----
CGCGTCC-CGGCGCGGAGGTTTAAAGATCCT-CGGGGGGT----GTC-GCCCGTCTGCCT---CGGT-

TCGGGG----CGG-CGGGC----CCTC-----------GGGGGAGC-GTCT--
TCCGGAGGGGCGTCCTCA---

TCCCCCTCTGGTCCTCCCTCCC-----------GGA-------CCCTGAC----------
CCCAGGC--
CCTGGCGTTGCGCGCCC-GCATC-----CACCGGCCC-GTCGGTCGCCGGGAG------------
TTTTGGGAACCCGGTGGCTGCCGCCG---
-----CTTAAGGCCGTGTGGCTTCTTGCCGTTGCGCGTTGACAACACACGCCTCGTGTCTCTGGGGGCGGG--
TGGGGACCCACT---TGGGCGCCTGTGAGGG---CGGCGTCCGC---GCCCGTCCT------------
GTCCTCCT--------------------GGG--AC-GGCGTTGT-GGGGGCGCTGCCCCG--GTCCC------
TTCCGTG----------CCA-TCC--GACCTTTCC--------GGGAGT-------
CTGGCTTCTCGTTTTT-------GTCTT---
CTGGGCCGGGCTGAGGCGCCCCCCCGACGCCCTCCTGCTGCACGCCGCCCCCTTCCCACTAACGGGAAAGGAACG
GGTGGCCGGCGGTGCGGGTCGTGGAGCGGTTTCGGGGGCAAGATTTGCCGC-GTC-
GGTGGAAA---
AAGGAAACCAAAGCGT--TACGACTCTTAG

Loxo
GGAAGGATCATTAACGCGA----------------------GTGAGCCGGGCGTCGCCGCCA------
GTGCGTGCGTCCCGGCCGCGCGACT--
CCGGCGCGGTGGCG------CGGGC-GGGCC-----
GGCGCGGTGCC--
-GGGTCTGCCGCGCCGCC---AGAGAGAGAGAGACAGAG--
CGCG--
TCGTGTGTGCGTGTGTGTCGG-
TGGGGGGGCGGCGACGGCGGGGGTCGGTCGGTCGGTCGGCCGGGGGATGTGGTCGC-------------------
----
GGAGGGGGGGAGGTGCCGCGGTGGGACGGGGGGGCGC--------------------------------------
----
--TGA---------
GCCCCCCCTCCGCCGCCGG--

TCGCCCTCCCCACTCCGGCGGACACGCCGCCGCTGCCGCCGCCGCCGGCCGGCCGCCCGTCGGGGCTCTCCGCGG
CCGCGTCGGCGCCGCCGTCGTCTCCCTCTCCGTCTCGCGC-----------------------------------
---
GAGGTGGAGTTGAGAGCCGGGGGGCCG--
TGCCCCGTCTCCGGCGCCGGTGTCCCACCCCGGTCGCCCGCCCCGC-------------------
CCGTCGGGCGTACGCGTCCGCGGC-GCGTCC---CGGGACGCGC-
GCGGTGCCGCGGGAAGCCCCCTCCCTTCCACCCCTACGCGCTCCCCCTCCCG-----------CGGGA------
GGATGGGGGGGCGCCGGTGGGTGGCGCTCTCAGAGGAGTCACCCGCTGCCCGCCGCT--TCCCGG-CCGACC--
CACC--
CTGGGCGTGGGGGAGGTAGCCCCGCGCCCGTCCTCCGGCCGACCGCCCCGGCCCAGCGGCCCCTGAGCGGCCGCC
CCTTCCCTCCGGCCCCCCTCCCCCTTGGATTA---

CTTCCACCAAACTGACCTCACCCGGCACCTTCGGGCCCTCGCCCGGCCTCCCCC---TTCCCGCCCGCG-

AAACCGG---GTCG--
TCCCGCCGTCGCCCCGGACCC--
--------------------------TCCGCGGC-----------------CAGGGGCCTGGGCCGCGCGGGT-
GCGGGGGGGGACGGTGGCCGGCCTCCGAGCGCGCCCGTGTGGGTCGTCGCGGGGGTGAAGGGAAGAGGAGGG-
----
CGGTGTGTGCGTGTGCGGCCGGGAGGTGGGGGGAGCCGTAGGCGGGGTGGGCGGCCGCGCGCTGCGGGCGGCGGG
TCCCCGGTGGCGGCGGCGCGGGGGGGCGCGTGGCCCCTACCGTCCATCCCCCGCGTC--ACGGGCCGGCA-
GC---GCCGCGGTCCGC-GCGTCCCCCGCGGGCGGCCGCGGCC----------------
GGGGATCC---------
GCGTCGGGGG--
CGATGGCCGCGCCCCGCCCTCGCTC-GCC----------ACCCTGT-CCAGGTACCTAG----CGCGTCC-
CGGCGCGGAGGTTTAAAGACCCT-CGGGGG------GTC-GCCCGTCCGCC---GCGGT-TCGTGG----CGG-
CGGGC----CCGC------------GGGGAGCGGCC----
GTGGGGGGGGCCTGGC---

CCTCACCCCGTCTCTCCCCC--------GGCC------TCCGCCCC-----------
CAGGC--------------------------CGGGGGGCTCCGC---------------------
GCCGCGCCCCGCTGCC-GGCCGGGCGGCC-GCCGGGAGGGGGA----------------------
GCCCGGCGGCCG--
GTCGG--
----ACCGCGCGGGCCCGCGCGCGGGCGCCCCGTGTCG----TTGGGGG--TGGGAACCCC------
GGGGCGCCTGTGGGGG------CGTCCAG---GCCCGCTCC---------------TCCG--------------
GGGGCCGGGTGT--GGACGG------------GCCCCT-TGTCC--------GCGAAT----------CCC-
TCCC-GACCTTTC---------CCGAGT------CGTGG-TTC-CGTCGT--------GTCTT----
TGGGCGGGCAGGAGGCGCCCCTC--
----------------------------------GGGGGATGTGTGCCGT-
GTCCAGGGCGGGGTCGCCTCCCTCCCTCCTC---------------
GCTGGCGGGGGGGAGGGCCCCGC---------GAATCCAAACTC--GTACGACTCTTAG

Procavis
GGAAGGATCATTAACGGA------------------------
CGAGAGAGGCGTGCCGGCGGGGGGCCGCAACCCCCACGC-----
GGCCGCCGGGATGAAGAAA----------------------------CACCCGCGCGGCGGCG------CGGGC-
GGGCC-----
GGCGCGGTGCC--
-GGGCCCGTCGCGCTGCC---GGAGAGAGCGAGAG------CGCGCGTCGGAGGG-------
CGGGGGGACGCGGAGTCCCACGGGTTCGGTCGCGGAGCCGGCTGC---------------------
GGCGGCGGCGGTGGCGGC--
GGGAGGACGGAGGAGGTGGAGGACGAGGGGCCGGG--

TACGGGCAGGGGTTCGGGGG---

------------------------------CGGT--------
CCCCCGCCCCGCCCGCCCCCCGCCCG---

CCCCTCTCCCCCCTCAACCCCTTCCCCGCTCCCTCCCCGCCCTCCCACCCACCCACCAACCCACCCACCAACCCT
C-CGCGCGC-------------------------------CA--
GGTGGAGCGGGAGCCGGGCGGGGCCGGGGTAAGCGTGCTGTCCCCGCGGCCCCC-------------------
ACCCTCCGTGTCTCCGACGCCGATGGCTCCCCGCCTCGCTCGCTCAACCCCCGCCCG---
CCCGCCCGCCGGGCGTTGACCGTTCCGGCGGC-GCGTC----
CGGGAAGGTGAGGGGGTCGGCGCGGTGGCCCCCAACC--------------------------------------
CCCCCTTC-----------------------------GGTTCGGGGGCGCCCGTCGCCCAGGAAGCC--
TCCCGG--CGCC---GGCC--CTGGGCGTCGGGAGAAGA--
CCCGCGCCCGCGTCCCGTCCTCCGTCCGCGGGGGCCCCCCGAGACGGGGGCGCCCCTCTCCG-
CGGCCCTCCGCCCCCCTGCGCC---

AGCGGCCCCCGCCGGCCTCCCCGCCCTGCCGACCCGCGCGACCCAG--CGTCG--
TCCCGCCGCCGCGCTTCCCCCACCCCGAG--
----------------------------CCCAGT-----GCGGCGGGGTGGGGGGGGCCTGGGCC--
ACGGGGCGCGCGGGCGCGCGAA---

GGGACGGGGGTGGTCGGTGGCGCGGCGCCGGCCGCGTTCGAGGGCGGAAGCCGACGGTGGGGGGCGCCGCGCCCG
GGCCCCGGGGGCGGCTGTGGCGCGCGGGCTCCCGGGCTCTTCTACTCCGTCGTC--ACGGGCCGGCA-
GCGCCGCCGCGGTCGTTCGC-------
GCGGGCGGCCGCGGCCGGCTGCGCGGCGGGTCGGGGAACGCCGCGTCGGGCGTCGGGGGC-
CGGAGGCCGGGGCGC------------------------------------
GGCCTCCTTTACCCGCCCCGCCCCGCCCTCGCCC-ACC----------ACCCTGT-CCAGGTACCTAG----
CGCGTTC-CGGCGCGGAGGTTTAAAGACCCT-CGGGGGG-----GTC-GCCCGTCCGTC----CGGG-
TCGGGG----CGGGCGGGC----CCGC----------
GGGGGGAGTAGTAGGCCGTGGGCGGGGGAAGGC--
----------CGTGTG---------------------------------------
CTTTCTTTCCCCCCTCCTCCTGCGTCTCTCTCCCCC--------GGAC------TCCGCCGAC-------
CCCCAGGC--------------------------CGGGGG-------------------------------
ACGCCC-
GCCGCG---

---GTCCCCGCGTGCCTGGGCGCCCCCACGTGTCTTGGGGGGG--TGGGAACCCCC------
GGGCGCCTGTGGGGG---GCCGGGCCGG---GCCGGGCCGGGCCTCGCCCCC----TCCT-------------
TGGGGGCGGG-ACCGGACGG--CCGCCGGTTCCCCCCG--GTCC--------GCGAGT----------CCT-
TCC--GACCTCTCC---------GGGGT-------CTGGAT-CCCGTCGTCT------GTCTT--CCTGGCC-
GGCCGGAGGCAAAGGAAACCCCCCCCCCACTTCCCTCCCAGCTC-------------------------------
----GGGAGGGGGCGGAGGGGCGGGGGGTAAGACACTGGGCCGT-GTC-
GGGTTTGGGGGAGGTCCCCCACCGCCT---------------GCGCGTGGGGGGGACCCTCCC--------
GGAAATCGAAAACTC--GTACGACTCTTAG

Pteropus
GGAAGGATCATTAACGAA------------------------GAAGGCAGCGGCGGCGCCTTAGC---
CGG-------CCGGCCGCTCGCCCGAGTGCTCT------------------------------
CCACCGACCCGTGCGGCG------CGGGC-GGGCC-----
GGCGC--

GCCAGCCCGGCCCGCCGGCCGCGA--------CGACGG----
CCA--

GAGGGTG-TGAGGGGCC---
CGGGGTGTGTTGGGGGGCCGCGCCCGGCCCGGCCCCAAACCCCCGCGCCGGCGCCCTCGTTCCGCCCCGGCGCCG
GTCGGCTTCTCCCGC-CACCGTCCGCGGAA------------AAAAAAGGCCTCGGC-GCGTCT---
CGGGGACGCGGCCGGCGCGGGCGGGGAGGGGCGCC--
-CTC---------------------------------GGCTTCCCCCCCCCCGCGCGACGGGTCTCC--
CCCCG--CCGCCC---GCC--CTGGGCG-
GGGTGGTTGGGAGGGAGGGCTTCGGCCTCCTCCCGTCCACCCGGCGGCCGCCTCCGTCCCCGGCGCCCGCCGCCC
TCCCCCGCCCCCCCTCCCCGGCGTCTCCCCGCCTCCCGCGACGCGCCGCC-------------------------
----
ACGGGCCGTCGGTTTCCCCCCGCGAAGCGTCCGTCGTCTCCCGCCCCCGCGCCCCCCGCCCGACTCCCCCCACCC
CGTGCCCCCGGCCCGGGCCCCCGCTGACCGCCGCCGCCCCCCGCC------------------------------
---CTCCGTG--------------
GCGGGGGGCCTGGGAC--GCGGGT-
GTCCGGGGCTCGCGGGGCTGGGAGGGGACTGGGGGCGGGCGGAGGTCGGGGG-----------------------
----
TAGGGGGTCCCGGGCCCGACGCGGGGGGGGGCCTCCGCCCGGCCGGGCGACGTCGTCGCGGGAGGGGCGCCGGGT
GGCGTGGTGTGGTGGTGGTGGGGGGGCGCGGAACCGGTGGCGGCGGTGGCGCGGGGGCCCGCCCCGTC--
ACGGGCGGGCA-GCGC-----CGAGGTTTTTCCGGGCGAATGGTGGCGGGGGGCGCGGTC----
GGCGTCGAGGCGCGGGGCGTCCCGCGGTCGGGTTGGCGG--CCGGAGCCGGCC----
GGACCCC--------------------------------------TTCGACCGTCCGCCCTCC--
ACCCTCC-----CCCCTC-T-CCAGGTACCTAG----CGCGTTC-CGGCGCGGAGGTTTAAAGACCCT-
CGGGGG------GTC-GCCCGTCCGCCC---CGGG-TCGGGGG---CGGACGGGC----CCGC------------
GGGGT-----------
TCGGGGAGGCCC---

GCTCCTCCTCCCCC-------GCGGC------
TCCGCCCCCCACGCCCCCGCC--
----------------------------------GGCG-
GTCGCGGCGC---------------------------CAGGC-------
GCCGCG---
-------------------------TTCCCCGGTGGCGGTGTGGGGGGG--CGGGAACCCCC------
GGGCGCCTGTGGGG-------TGCCCGT---GCGCGGCCCC-------------------------------
GCGGGGCCGACGCCCGGG--------------CGCCCC---GTC---------GTCGAA----------
CCGCTCC--GACCCCA---------CGCGAGT-------CTGTT--CTC-------------
GTCTCTCGCTGGCC-
GGCCGGAGGCGCCCCCCCCCTTCCC--

----------TCTGTGTGTGGGGCGGGGAGGGGGATCGTGCCGT-GCC-AGG-GGTTGGGG-
TATC--------------------TCCCCGCCGAACC--CCCAGGAAAAAAAGAAAAAAAAGAGAAACGCT---
G-ACGACTCTTAG

Myotis
GGAAGGATCATTAACGGA------------------------
GAAGGGGGCGTCCGTCCGTCCGCGTTCGG-------
TCGGTCGGGTCGGTGTCCTCTTTGGCTTTT----------------------------------
GGCGGCG------
CGGGCCGGGTCGGGGCGGCGGC---
GGCCGCCGCCTCGCCCCCCGCCGGTCGCGA----------
GAGAA--

GGGGCGAGCGG-
GGGCGCGGAGGGGGCCTTCGGGGTGCCCCCGTTGCCCACGCCCGCCGCCTC----------------
GTGGTTCTTTGTCGCCCTCACCCCTGGCCGCGAGAATGCAAGGGCAC---------
GCGTTGTGGGCGTTCCCCCCCGCAGGC-GCGTCT---
CGGGAACGGGGCC--
GCGG---TGGCCCCT--
TCCCG--CCGCCT---GCC--
CTGGGCGCGGGTCGTCCCGTCCTTCCCGTCACGAGCACCCCCGTCCCCCCCTCGTCCGCCCCGCGCCTCTCGCCT
GCCGTCCG---

TCGCCCGCGGTGCGGTCGGGGCCGCGCGCGCACACGCCCCGCGCGTTCCGGGTGTCCTACCTCTCCCCTCCCT
CGCACGCCGTGCCCCGTGCCCC-AGCCTGCACCCC-
GCTTCCCGCCGCAGCCCCTTGC---
-----------------------------CCCAACGTG--------------GCGAGGGGCCTGGGCC--
GCGGGT-
GCGGGGACGGACGCGGTGGTGGAGGGAGGAAGGGGGGGCGCGGCCCCCCGGGACGCGTGTC------------
----
GACGTGTGTCGCGCGGGGTTTCCCGGCTTTGGCGTCCGCCGGGCGTCGGAAACGACGGTGGTTGGTCGGCGCGGA
AGGGGCGTTGGGGGGTTTAGGTGCCCGTCGCCGGGTCGGCGGGCGGCCCCGTC--ACGGGCAGGCA-
GCGCC----CGGGGGGCTCTGCGCCCCACGCGTGTGCAGGTTGCGG------------
GAACCGTTGGGGGTTGAGGCGTCGAGGCCGCGGAGGCGGAGGCGCCCGTGGGGAC--------------------
----GTGG----TTCCCACCCCGTCCGCTGTGTCCGCTC-GCTCC-------CCCCTT-T-
CCAGGTACCTAG----CGCGTCC-CGGCGCGGAGGTTTAAAGACCCC-TGGGG-------GTC-
GCCCGTCCGCCT---CGGG-TCGGGGG---CGGTCGGGC----CCGT------------GGGGAGG--------
GTCCGGGGG--

GAT--
CCCCCGCTTTCTCTCCCCT--

-------------------------------ACCCCC------GGGCGCCTGTGGGG-------CGTCCGA---
GCACGCCCC----------------TTG--------------GGGGCCGTTGCCCCGGAC-------------
CGCCCC---GTG---------GTCATA----------CCT-TCCC-GACCCG-----------
CAGAGT-------CTGGT--CTCGTTTGTC--TCGCGCTCGGAC--GGCC--
GCCCGAGGCGCCTCCGCCCCACCGCCAGCGGGA--
-------------CGTGGTGGGGGGAGAAGAGAAGGTTGCCGT-GCC-
GGGGAG---------------------------------------CGAAAACCAAAGTA----------------
ACCAAAACGTT--G-ACGACTCTTAG

Canis
GGAAGGATCATTAACGGAGAA----------------
CGCGGCGGCGGCGGCGGCGGAGGCGCCCCGTCCTTCCGTCCGTCCGCTCGCCCGCGAAGCCTT------------
-----------------------CC-CCCGTGCGGTG------CGGGC-GGGCC-----
GGCGC--
GGTGCCGGCCCGCTGGCCGCGAGGG-------ACGTGAGAGA--GCGGGAGCGCGTGTGTGCGTGTCC--
GTCCGTGGGGCGAGGACGGGGCCGCGTGAGGAGGAGGCAGGGGGAAGGAAGGGGCCGCGCCCGCCGCCGGGAGGC
GCGGTGGAAG-CGCG-
GCGGTGGCGGTGGGCGGCCCTGGCGTCGGTCGGTCGGGCGCCCGCCGGGAGGGGAAGGGAGGGGGTGT----
CGTCGGCGACGACGGCGTGTCGTGGGGGGGGAGACGGGCC-----------------------------------
----
---CCGCG-----
CCCGCCTCCCTTTCTCGGACGTCGCCGTCCCGGCCCCCCC-----------------------------------
----
CCACCGTGTCCCTTCCGGGGGGGGTCCGCCTCCTCCGTCTTGCCCGCTCGCCTGGGGCTGCCGCCGGGTCGTCCA
CTCGCCGCCCCCTCGGCGGCGGCGGCGGCGGCTCGTCCCCCTGCCCCTCC-------------------------
--------------GGGGCCCCCCTCCCTTCCGCGG--------------------CCCGCCTT--
TCGCCTTCCGGCGCCGGTCGTTCCCC--------------------------
GGTCGCC---------------------GCGTCGCCCCGCACGGGGG-TGCCCTCGGC-GCGTCT---
CGGGACGC-GTGCGACGCGG--CGGCGGTGCCCTCCGCGGTGTCGTCGTCGTCCTCCTC-------------
CTGC----------GGGGGGGGGGAGCGTCGGCCCGCGCGGGGGCGCCCGCCGCCCGGCGCGCGCC--
TCCCG--CCGCCC---GCC--CTGGAC-
GGTCCTCCGGTCAGGCGGGCGTCCGCGCCATCTCCGCCCTCCCGCTCGTCGCCTCTCCCTCTCCCACCCCGGCCC
ACCCCCCGCCTCTGGGGCCTCGGGGCC-------------------------
CGGGTCCGGCTCTCCCCTTCTCCGTCGCCGCCCCCGCC-------------------------------------
------------------GCCCCCCTCCCCTGGCCCTGCCC--GCTC-ACGCCCC-
GCTTCCCGCTGCGGGCCCCCCCCCGCCC---
--------------------------------TCCGT------GGCGAGGC-----GGGGCCCGGTC--
GCGGTT-
GTGGGGAGGGCCGGGGGTCGGCCGGCCGGGTGTTTTCTCCTAAGGCGAGGCGAGGTGGTGGCTCCCGGGGGTGTT
CCT---------------------

CGGAAGCGAGGGGATCCGGCCGGCCCCGCCCCCGGTGGCTTTTGGGGGCGGGGGAGAGACCGGGGAGAGAGAGGG
AGGCTGGCTGTCGGGCGGTGGCGGGGCCCGCGTCGCGGCGCCCGCCGGGCCCTGGTCCGTC--ACGGGCCGGCA-
GCGT-----CGGGGGCTCGCGCCCCTCGCTTGGCGGTGTCGGCCGCGGCCGGCC--GGGGGGAGGA-------
TCGGCGTCGGGGCGG--------------------------------------
CGGCGGCGGCGGCCTTTGGCCTCGCTCCCCGCGCCCCGCCTCGCCC-GTCCCCCCCC-ACCCTCTGT-
CCAGGTACCTAG----CGCGTCC-CGGCGCGGAGGTTTAAAGACCCTTTGGGG-------GTC-
GCCCGTCCGCCT---CGGG-TCGGGG----CGGTCGGGC----CCGT------------GGGGAGG--------
GTCAG--

TCCCCTCCTCC--------AGAC------
TCCGCCGCCTCGGTCCCCGCCACCCCGTGGGGTGTG--------------
CGGGGGGGGT---------------------CGCCACGCCACGTGCC--CGGTCCACGCGGCC-
GCCGGGAGGGGG-------------CTCGCCCCCTGCCCGGTGGCA---GTC--GTGCCGCGTCC----
GTGTGCGCG---
TGCGCCCGCCCCCCCCCCAGCGTCCCC--GGTGTG--GCGTGGGGCGGGGCAGG--TGGGAACCCCCT-----
GGGCGCCTGTGGGGT------TGTCCTC---GCTCGCCCCCCGCGCCCGCG------CGTG------CGCGGC-
GGGGGCGGCGGCCGGAC--------------GCCCC---GTCGTTC-----GTCCGA----------
CCCTTCC--GACCTTCT---------CGGAGT-------CTGAT--CTCGTG----------GTCTCT-
GCTGGCC-
GGCCAGAGGCGCCCTCC--
-----------------------------GGGGAT--GTGCCGT-GCC-
AGGGAGGGGGG---------------------------------------CTCTCCCTCC---------------
GCTCGAAACTC--GTACGACTCTTAG

Felis
GGAAGGATCATTA-----------------------------CGAGAGAGCGCAGCGGTCGGGGCCCTCCCCCT-
GTT--CCGCTTGCGTGCCTTG------------------------------------CCACC---
CGTGCGGCG------CGGGC-GGGTC-----
GGTGC--
GGTGCCGGCCCGCTGGTCGCGAGGG-------AC--GAGAGAGCGCGGAGGGCGTT--------------
GAGGGGCCTTTGGGGGGGGGGCGGTCGGAGGGGG---

TGGGGAAGA---AGGGCGGAGGCCTCCGCCAGGAGGCGC-
GCGGGAGCCG---

---------------------ATG--------
CGGCGTCCCCC--

GCCGTCCCTGGCGTGCCTTCGCCCTCCTCTCCCCGCCCCTCGCTGGCGCGCTTCCCCCCCCTCTGCGCCTCGCCT
CGCCTTCCCCCGC-----------------------------------GGAGG-
TGGGTCTGAGGGTTGGGGGGGTTCGAGTGCGTTCCCCCCGCCTTCTTCGCCTT-
TGGCGCCGGTCGT--------------------------AACCCGGTCGCC---------------------
GCGCCGCCCGCGCTGGCGCCCTGGCCGCGTCGCGTCT---CGGGAT---GCGCGGCTTGG--
CGGTGGGTCCCCGTGG----------CGTC-TCCCCGGTCGGGTT--------CTCG-
CCTGTCCCGGGGGGCCTTGGAGCCTCGGCTCACGCGGGGCGCCCCGCCGTCC----GCCGCC--TCCCG--
CCGCCC---GCC--CTGGAT-GGCTCTCCGCT-

CCGTCGGGGGGGGGTCCGTCCCCCCCGGGCCCGTCTTCCTGCCTTGCGCGCCTCTCTTTTTTGCCCTACCCCGCA
GACCTTTTACCCCCCTTCCTTCCGCCCTCCCTTCAGGCCCCGGGTCCGGTCCGGGTCCGGCTTTCCGTCTCCT--
----
TCCCCGCCTCCTCCCCGAACCGCCACCCCCACGCCCTTGCCC--GCTC-GTGCCCC-
GCTTCCCGCCGCAGCCCCCCTCCC---
--GCGGGG---GGGGGGCCTGGGCC--
GCGGGTGCGGGGGAGGGGAC--
GGTGAGGGTTGGTCGGTGCCACGGGGAAGGTGTGCTCGGAGGTGAGGGCGGGG----------------------
----
CCGGGGCCGGTCGGCCCCGGCGGCCCTGGATGGGGGGAGAAGAAGGGTCGTGTGGGGGTCGCGGGGGGGTGGGGA
AGCGAGGGGCGTCGGGCGGCGGCGGTCTGTGGGG-GCGGTTAGCCCCGTCGGAGCCTCCATC--
ACGGGCCGGCA-GCGT-----CGGGG-CTCTGGGTTGCCCTTTGTGGGT-CCGGCCGCGGCCCC----
GGGGGGGG--------GTCGGCGTCGAGGTGG-------------
TTGGCGGTGTTGCGGG---------------------CGCTCC--CTCG-TCCCCC--GCCC-ACCTCGCC--
GCCTCC-------ACCCTGT-CCAGGTACCTAG----CGCGTCC-CGGCGCGGAGGTTTAAAGACCCT-
TGGGGG------GTC-GCCCGTCCGCCT---CGGG-TCGGGG----CGGTCGGGC----CCGT------------
GGGGAGCC-------
GTGGAGGCTGG--

TCTTCCCGTGTCTCCTCC--------AGAC------TCCGCTGCCC-
GCCCCCCGCCGCGCC-----------------------CCGGGCCGGGG------
TGCCGCCGCGCTTGCGCCCACGCCACG-GCCC------TCCCCGGTT-
GCCGGGAGGGGG--------------------CTTGCCCGGCGGCT---GCCGGGTGGTGCGTGAGC--
GTGTGCG--
TGTGCGTGCGCCCCGTGTCCCTTGGGTGTGTGTGGGGGGAGGGGACAGG--CGGGAACCCCCT-----
GGGCGCCTGTGGGGT----TTGTTCCGA---GCTCGCCCCGCGCG----------TCCG--------
CGTGGCTGGGGCGGGCTGCCGGAC------------TTGCCCT---GTCGT--------TCCAA----------
CCCTTCC--GACCTCTT----------GGAGT-------CTGGT-CTTTGTT----------
GTCTCTTGCTGGCC-
GGCCGGAGGCACCCTTC--
-----------------------------GGGGAT--GTGCTGT-GCC-
AGGTTGGGGGGGCGCCCCTTTT------------------------CGGGGTTGGCCCTCCCGGC-------
GATCACAAAAACTC--GTACGACTCTTAG

Equus
GGAAGGATCATTACC-----------------------------
GGAGCGGCTCGCCGC-------------------------
CGGCGGCCGAGCCTTTTC--------------------------------ACCC-CCGCGCGGCG------
CGGGC-GGGCC-----
GGCGC--
GGTGCCGGCCCGCTGGTCGCGAGAG-------GTTCGAGAGAGGGAGGAGGGGGCGCGCGGGAGGCGC----
GGGCGCCCGGCC-------------------GGAGGCGCGGGAGGCG------------
CGGGCGCGCCTCGG---

TCCCGGGTCGGCGGTGGTCCGGAGGGAGTGGGCTCG---------------------------------------
----

---ATGTC--
GGCGAGCCCACGAGCCCCT---

TCCGGCCTCCGTCCGCCCGGCGTTCCGAGGCCGCCGCGTCCTCCGCGCCGCCGCCCCGGCGCGTCCGTCCCCGC
GCC-----------------CTCCGGTCTCCGG------GAGG--CGGG-
CCGGAGTTTGTCGCGCCGCGCCTCC---
----------------GTCTCCGGCGCCGTCCGTCCCCGCTCTC----------
GCGGGCGGCGGCGCCCTCGGC-GCGTCT---CGGGAA-GG--GCGGCGTGG--
TGGCGCGAGCCCCCGCGGAGTCCCCGGGGTCAGGTCCCCC---------------------------
GGGGGGCCTCGAGCCTCGGCTCACGCGGGG-TGCCCGCCGGCCGCCCGCCGCC--TCCCG--TCGCCC---
GCC--
CTGGGCCGTTCCCCGGCCGTCGGCGTCGTCGTCCGTCCGACGGTGCCTCCGCGTCTCTGCCCGCCCTCGGCGCCC
CGGGCGCCCGCGTCCGGCCGGCCACGTCCGCCCA--

--------CCCCCCTCGCCCTGCCC--GCTC-GCTCCCC-GCTTCCCGCCGCAGCCCC--
TCCC--
-----------CTCCGT------GGCGAGA------GGGGCCTGGGAC--GCGGGT-
GCGGGGAGGGTCCCC---

GGGGGGAAAGTCGGGTCGGGTCTGGTGGCGTGCGTCGGACGCGAGGGGGCCCGCCCGGCGTCGAGGGGGCCGTGG
GCGCAGTGGGGTCGTCGGTCGGCGGCCCCCGGCGGCGGGGTTCGGTC--ACGGGCGGGCA-GCGC-----
CGAGGCC--TGCGTCCGTCCCC-----------------GGGTGGCCGCGGGCGGCGC----
GGGCGGCGTCGAGGCGGTGGCCGCGCGGCACTACCC-------------------------------
GCTCCGCCTCG----------TCCGTTTCTTT--GGCTTC-------CCCCCCAT-CCAGGTACCTAG----
CGCGTCC-CGGCGCGGAGGTTTAAAGACCCC-TGGGGGGC---CTCG-GCCCGTCCGCCTT---GGG-
TCGGGG----CGGTCGGGC----CCGC------------GGGGA----------
GTCGGGGAGGCCT---

GGCCCTTCTCCCC--------AGAC------TCCGCCTTCG--------
CCGGGC--------------------------CGAGAG-----------------------------
GCGCCGGC-GCCCC--GTCGCGGCGGCC-GTCGGGAGGGGGCG--------------------
TCCCCGGCGGCC---
GTCGTGTCAT--
-----------CGCGTGCGCGTG-CGCGCCCCGCGTCTT-CGGGCGAGG--CGGGAGCCCC-------
GGGCGCCTGTGGGG-------TGTCCGA---GCACGGCCCC--------------------------------
GCGGGCCCGTGCCGGAC--------------GCCCC---GTC---------GTCCAA----------CCT-
TCC--GACCTC----------ACGGAGT-------CTGGT--CTCGTTGT--------GTCTTCC--TGGCC-
GGCCGGAGGCACCCTCC---
-----------------------------GGGGAT--GTGCCGT-GCC-AGGGGCGGG-
CTCTCCCCC----------------------------------GCCCTGCGGGGGGACCCCGCC-
GTTCAAACTC--GTACGACTCTTAG

Sus
GGAAGGATCATTAACGTT----------------------
GGCGAGAGCGTGGCAAGCGGCGGCGGCCCGCGTCTGCCTGCCCGCCCGCCCTGCTCGCTCTC-

TCGCCTCC--------------------TCCACC---TGCGCGGCG------
CGGGACGCGA---
--------------------------------------GGG-
CGGGAGGGAAGGCGCCTGGAGGTGCGTGTTGCG---
CGGCGGGGGGGAAGGGGGGGACTCACCTGGCCGGGGGTTGGTGCCCTTGTCGGGTCACCC---------------
------------------------------------GGG--GGTGGGAGGAA-
AGCGGAGAGAGAGGCTGCTCTGGGGGAGTG-----TGGATGGCATCCGAGGGACCGGGGT---CAGG--
CGGC---

-------------TTG----
GCCGTTCCTTTCCCCGGCCTGCTCCCCCCACC---

ACGCGCCTTCTCCTCCTCCTCCCCCCCGGCGATCCCGGCTGCCACCGCCCCCGTCCCACCCCCCCCCCGACGCCC
CGTGTAGTGCTTGCACGCATTCCGG----
CGTTTCTCTTCCGCCTGACCTCCCCGCCACGT----------------CGCCGA-------------GAGG--
CGGGTCCGAGGGTTGAT------CCGGGGCTTTTTGGGGG-
GGGCCGGGCCGTGCCCTGCCCCCCCAACCCCCGCGC---------
GTCTCTGTCTCCGGTCGCGC---------------------GGCCTTGGCTGCCCCGTGGTGTTTTTGGC-
GCGTCT---CGGGAT-
GGGC--
TTT---GCCC-TCCCG--
CCGCC---GGCT--
CTGGGCCGGGTTTCCTCCCGGCCGTTGGTCCTCCATGCCTCC---------------------------------
----

-------------GCTCCACTGCTCCGATCCTGCCGCC--GCTTG-
GCCCGGGC---
---------------------CCTCTCTCCCTG-----------------
TCCTCGGCGGCGGCGGCGGCGG---

CGGCGCTGCTTGCGGTGGGGGCGGTGGTCGGCCGGCGGCCGAGTGGCTCCCGCGTC--ACGGGCCGTCA-
GTGCCAC-GTAAGACCC--
GCGTTCCGAGGGCGGGCCGCGCGACGGACGGAGTTCGTTACCCG-------------
TCGTCAGGGCCGGCGGT--------------------------
CGTTGGGGGCGCCTGGGTGCGGCGCTTTCCCCCCCTCCCTGCCCCGCCTCGCCC-GCCTCC-------CTC-
TCT-CCAGGTACCTAG----CGCGTCC-CGGCGCGGAGGTTTAAAGACCCCTAGGGGGG-----GTC-
GCCCGTCCGCCTT--GGGG-TCGGGG----CGGTCGGGC----CGGT------------
GGGGAG------------
GAGGAG---

TCCCTTCTCCCCC--------AGGC------TCCGCTT----CCCCCCA-
TGGGG--------------------------CGGGGCCCC---------------CGTGCCTCGTCGCGGTCCA-
GCCCCTCTC-ACCGTGGCC-GTCGGGAGGGGGG-------------------CTTATTCGGTGGCC---
ATCGCCGGCCAGTCGGTCC-GTG-GCCGTGTGCGAG------------------------------
CGA--------GCGCGTGTGTGC--------------CCCGTGCCTGTGTTGGGGGGG--

TGGGAACCCCC------GGGCGCCTGTGGGGT------TGTCCGT---GCCCCACCCACTCAC--------
CCGTGTGT-----GTGTGTGTGTGTGGG-CGCCGGAC--------------GCCCC---GTCGT-------
GTGAAG----------CCT-TCC--GACCC------------ACCGGT-------CTGCTTT---
GTTTCCC-------TCT-GACTCGGCC-
GGCGAGAGGCGACCCCCCCCGCCCCCCACGCTCTCCACCCAGCTGCCCCACGT----------------------
GGGCGGGTGGGTCGTGGGGAGGAGGAGGGGGGGCAGTGTGCCGT-GCC-
GCCATTCC---
AAACCAAGCAC----GTACGACTCTTAG

Tursiops
GGAAGGATCATTAACGTGGTC--------------------CCGAGGGCGCGGCGGCCC--------
ACCCGTCGCCCGCTCGCGG----ACGAGTCTTCC-------------------------------CCACC---
CGTGCGGCG------
CGGGG--

CGGGGAAAGGAAGGGGGGAGGGGAGGCGACC-----GGGAGTCGGCACCCGGCGCGCGCGCGCGTG-
CGTGTGCCGTGCGCGC--------------------GGGGGGCGCGGGCGGCCCGTCGGGTCGG--
TCGGTCGGTGGTCCTCCGCGGTGGGGAGGAGAGCGAGAAGGGGGGTGG-------------------
CCCGTAAAGGCGGGGGGGTCGGGTCGGCAGGGGCGGC--------------------------------------
----
--TCCAC------
GCCTCCCTCGCCGCGCCCGCCCGTCTGCCCCCCTCGCCACGCG--------------------------------
----
CCTCCCGCCACGGGGCGACGCCGTCCCGACCTCCCGGGGTGGCCGCCCGACGCCGGCCCCCGCCACGCCGCGTAC
CCGCGAAAGTCGGGGGGCTATCCCGGCGCGTCTCTTTCCCGCCCGACCTCCCCGCCACGT---------------
-CGTCCCTC-----------GAGGTCTGGGTCCGAGGAGGAGGAGGGGTCCGGGGTTT----------
GGCCGGGCCCGGCCTCCCACGCGC--------------
GCCTCCGTCCCCGGTGCCGGTCACGCCCAACCCC----
GTTCGCGACCGCCCCACACCCCGCGCGGAGCCTCCGGT-GCGTCT---CGGGGTTGGGT--GGCGCGG--
CGGTGGCGTGACGGTGGCGGCTCC---------------------------
CCCTG-------------------------------GGGGGCCGCCCGCCCGCCCGCCCGCCCGTCGCCT-
TCCCG--CCGCC---GGCC--CTGGGCCGGTTCCCC---
GCCGGTCGGTCGTGGGTCCTCCGCTCCGTGCCCTACGCCCCCTCGGCGGGCGCCTCTTTTACCTGTGTCCCGAGC
CCCGGGACCTCGCCTCCCTCCGTCGTCCGTCCGGCTCTCTCTC--------------------------------
----
GCTCTCTCTCTCTCTTTCGCGCCCTCCCTCCCCGTTGCGCTCCC-TGCCGGCGCTC-GCCACCC-
GCTTCCCGTCGAAGCTCCCCTTACGCCC---
--------------------------------TCGG---------------------
TGGCGACAAGGGGTGCCCCGGGAACGCGGGCGCGGGCCGGGCTAGGGCCGCGGGGGCCGGGCGTTTGGGGTTCAG
G-------------------------------------
AGCAGAGAGGGCCGCGTCCGGGCGCGAGCGTGGGGAAGGTCTCCGCCCGGTTTCGGGGACACGGGTGTGGGGGCG
TCGTCGGGCGGTGGCCGTGGTGTCTCGTGTGGCGGTCGGCCCGGCCCCCGGCCGGGGTCCCGCGTC--
ACGGGCCGGTA-GCGC-----CGAGGCCCCCGCGTGTT-GCGGGCGGCCGC-----
GGGCGGAGAAGCGTGTCG----------GTCGGTGTCGGGG-
CGGCGGT---------------------------------
GAGCGTGGGGGCGCCCCGCGCCCAGCCCCACCCCCCACGCCTCGCCC-GCCTC-------CCCCCTCT-

CCAGGTACCTAG----CGCGTCC-CGGCGCGGAGGTTTAAAGACCCA-TGGGGG------GTC-
GCCCGTCCGCCTT--GGGG-TCGGGG----CGGTCGGGC----CCGC------------GGGGA----------
GTCGGGAGGCCCC--

TCCCTTCTCCCCC--------AGGC------TCCGCTTCACCCCCCCCACCACCACCGGCACC--
TCACCACACACGGGGCCGGGGCGCCGCGCC--------GCCGCGGCGC-CGCCGGTCCACCCC-------
GCCGCGGCC-GTCGGGAGGGG----------------------CTACCCGGCGGCC---
GTGGTGCGGGCCGGGGCC--GTGCGCCGCGCCTGCGC----------------------------GCCCCGC--
GTCCCCTGTGTGTGCGTGCGCGCG--CACGGTCGGGTTGGGGGGGGCAGG--TGGGAACCCCCC-----
GGGCGCCTGTGGGG-------TGTCCGA---GCCCGCCCCGC--------------CCG--------------
CGGGGTCGG-TGCCGGGC--------------GCCCC---GTG---------GTGAAA----------CCC-
TCC--GGCCCCC----------TCCGGT-------CTGCTT----GTTTT------CTGTCT-GACTTGGCC-
GGCCAGAGGCAGCCCCTCC--
-----------------------------GGGGGAAGGTGCCGT-GCC-
ACCGGCGGGGACCTC-----------------------------------CCCCGCCGG--AAAAACCC-
AGAGAAACCAAAACTC--GTACGACTCTTAG

Bos
GGAAGGATCATTAACGCGGTC----------------GGTTCCGAGAGCGGCGCGGGGG------
CCTCGCCCCGCCCCGCGCCCGGCTCGCGAGCCTCTCCT--------------------------------
CCGCCCGTGCGGCG------
CGGGATGGGA---
---CCGGGAGGAGGGGAGAGGGGGAGAG---
CGAG-----
ACGCGTCCGGAGGGCGCCCCGCCCCCCACGGGCGGCCGGGGGCCGGTCGGGGGCTACTCCCCGGTGTGTCTGGGG
GGCGAGGAGC-CGCG--
CGAGAGAGGTGGGGGGGCGGCGTGGCAGAGTTGTGG---------------------------------------
----
GGATATCGGGTGGGAAGAGGCGGG---

--------------------------------TTCTCCT-----
CCGCCTGCTTGCCCGCTCGCGCCCCTCCTCCCGCCGCC-------------------------------------
----
GGCCCCTCCGCGCCTCCGTTCCCCCCCACCCGGCCGGGGGCGCCCCGCCACCCTGCCCCGTCGCCCGGTGGCGTC
GCCCTCCGGCGTGCGTCCCCTCTTCCCGATAGCCCGG----------------
CC---
NNNNNCCCACCCCACCCCACGCGCCTT--------------
TCGCCCCGTGGCGCCGGCCACGACTGCCTCCCCCCACAAAGGCCGCGGATCCGCGCGGCGCGGATCCTCCGGTCG
CGTCTGG-CGGGCTGTGCGGCGTGGCGGG-
CGGCGGCTCC---
CCGTC----------------------------------GCGGGGCCGCTTGCCTTGCCCCGCCGCC--
TCCCG--CCGCC---GGCC--
CTGGGCCGGTCTGCCTCGGCCGGTCGCCGGCCGGCCGTCCGTCTGCCGCTCTGGGCCTGCCGCGCCGGTCGCCAG
GCGCCTCGCCTCCCCACGCCTTCCGAGCCTCGGGGCTTTCTCCCGCCCCGTCCCCCACCCCCCACGCCTCCCGCG

GCCGTCTGTCCGGTTCTCCCGCCCTGTTTTCGCCTCTCCCGTACCTCTGACGCGTGTCCCCTGCCCGCTTGGCGC
CCA---GCTCCCCGTCGGAGCCC--
CTTCCC---
------TCCGCCCTCGG---------TGGTGGTGTGTGGGGGGGGGGAGGGTGCC-
CGGGAACGCGGGTTCGGGTAGGGGTCCCCCAGCGGATGGGCTGGGGCGAGAGGACGGGGTCCGGCGGGGCCGGC
CGCA-------------CCGGGTGTGTGAGGGTTGGAGGGGCGGGGGCGGGCTCTGCCCCGCGTCTC-
GGGGGGGGGACGGCGGGGGGGTGGTCGTCTGGCGTCGGCGGGGCCCTCCGGGGCCGGCGGTCGGCCGGCGCGCGG
CGGGGCACCCCCTGCGGTC--ACGGGCCGGCG-GCGC-----CGAGGCCC--GCGTG--------
CGGCCGCGGGCCCGGTGGGGGGAC---
GCGGCGGTTGGGGGTCGACGTCGGGG---
------CGTTGGCCCGTGGGGCGTGCCTGCCCCTCGCCC-GCCTC--------ACCCTTT-
CCAGGTACCTAG----CGCGTCC-CGGCGCGGAGGTTTAAAGACCCC-TGGGGGGGGGGTGTC-
GCCCGTCCGCCTT--GGGGTTCGGGG----CGGTCGGGC----CCGC------------GGGGAGTA-------
GTCGGGAGCT---

GCCTCCCGTCTCCCCCC-------AGAC------CCCGCCGTCCC-------
CCGAGGGGA-----------------------CGGGGTGGCGCGCGGC---------
GTGGCGCGCCACGGTCACTGCC-------GCCGCGGCC-GTCGGGAGGGGG---------------------
CTACCCGACGGCC---GTCGTGTGGGCT--------
GTG---
GCCGTGTGCGGCGGCGCGTGCGCGCG--CTGCCCCGCGTCCC-TGGGGGAGGGTTGGGGGCCCCC-----
GGGCGCCTGTGGGGT------TGTCCGA---GCCCGCCCTGG--------------------------------
CGGCTTGG-CGCCGGAC--------------GCCCC---GTCGT-------GTGGAA----------
CCTTTCCC-GACCCC-----------TCCGTT-------CTGCT-----GTTTT------CTGTCT-
GACTTGGCC-
GGCCAGAGGCAACCCCCTGCCCCAACTCCCACCTCC---------------------------------------
--------GGGGAGGGAGGCGGGGGTGGGTGGGAT--GTGCCGC-GCC-
AGGGGCGGGTGTCCC---------------------------------------
GCCGAAACGAAACCCTT------GAAAACCTC--GTACGACTCTTAG

Bubalus
GGAAGGATCATTAACGCGGTC----------------GGTTCCGAGAGCGGCGCGGGGGGG----
CCTCGCCCCGCCCCGC--CCGGCTCGCGAGCCTCTCCT-----------------------------CCACCCG-
TGCGCGGCG------
CGGGATGGGA---

AGGGAGCAGGGGAGAGGGGGAGAAGGACGA------
ACGCGTCCGGAGGGGCTGCCGC-----------------------------------
TCCCCGGTGTCTCTGGGGG-CGAGGAGC-
CGCGCGCGAGAGAGGTGGGGGGGCGGCGTGGCAGAG---------------------------------------
----
TGTGGGGGTCGGGTGGGAAGAGGCGGG--

-----------------------------------TTCTCCC-----
CCGCCTGCTTGCCCGCCCGCGCCCCTCCCCCGCCGCC--------------------------------------

----
GGCCCCTCCGCGCGTCCGTTCCCCGGCGTGTTGTCCTGTCCCCTCTTCCCGAA--
GCCCGTCCCTCCACGCCACGTCCCTCGNNNNNNNNNN--------------------------------------
----

-----------------NNACCTAG----CGCGTCC-CGGCGCGGAGGTTTAAAGACCCC-TGGGGGGG--
TTGTC-GCCCGTCCGCCTT--GGGG-TCGGGG----CGGTCGGGC----CCGC----------
GGGGGGAGTA-------
GTCGGGAGGT---

CCCTCCCGTCTCCCCGC-------AGAC------TCCGCCGTCCC-------
CCGAGGGGA-----------------------CGGGGTGGCGCGCGGC---------
GTGGCGCGCCACGGTCGCTGCC-------GCCGCGGCC-GTCGGGAGGGGG---------------------
CTACCCGACGGCC---GTCGTGTGGGCC--------
GTG---
GCCGTGTGCGGCGGTGCGTGCGCGCG--CTCCCCCGCGTCCC-CGGGGGAGGG-TGGGAACCCCCC-----
GGGCGCCTGTGGGGT-----TTGTCCGA---GCACGCCCTGG--------------------------------
CGGGTCGG-TGCCGGAC--------------GCCCC---GTCGT-------GTGAAA----------
CCTTTCCC-GACCCC-----------TCCGGT-------CTGCT-----GTTTTGT------GTCC-
GACTTGGCC-
GGCCAGAGGCAACCCCCTCCCCCAGCTCCCCCCTCC---------------------------------------
--------GGGGAGGGAGGCGGGGGTGGGTGGGAT--GTGCCGT-GCC-
AGAGGCGGGTCTCCC---------------------------------------
GCCGAAACGAAACCCTT------GAACACCTC--GTACGACTCTTAG

Sorex
GGAAGGATCATTAACGGAT----------------------CCCCGCCCGGCGCGTCG-CCGGACGGG-
ACGC----------TCTTCTCGCGAGC-GAGCGAGGAGAA------------------------ACC---
CCGTGCGGCGCGGGGGTCCCCCCCGGGAG--GAGGGCGGAGGAGGAGGAGGCGTCGGGGTCCG--
CCCCCTCCCTCCTCCCCACCCCTCCGTCCCTCCTCGCGGGAGCCCCCGCCGTTCGC--AAT---
GGGGGAGGCGCGCAGGGGGCGCCGAGGCGAGCTGG-----

```
AACGAAGGTCGGCAGAGAGTGGTCCCGGTCGGTCGGCGGTCGGGGCTTGCCGTGCGGCGGCGGCGGCGGCGGCGG
CCGCGGTGTCGGGGGAGGGGGCGGCGTCTCGACGTGGGATCTCGTCGGGGTTTTCGGCGCGGCCCGGGGTCGGGC
CTCGGGGCCGTCCCGGGGGAGGGTCGAGCGGGTGCGAACGGTCGGCGTGGCGC----------------------
---------------------------------------------------------------------------
-----------------------------------------------------------TTCTCCGCG-
TGTGCCGCTGGCCGCCGCTCCCCTCCTCCCCGGGGGCCCCTCCGTCCCCGGTCGGCGCCGGCTCCCTCGGGCCCC
GTCGCGATGCT----------------
TTTCCCCGTCCTCCGGCTTCCGGCCGCTCCGTCCGCCCGCCCGCCCGCGGCGGCCCTGACCCTCTTCCGCTTTGC
CTCTCCCGGCCCCGGCCCGTCCGGCGTCTTCCCTCGCCTCCTCC---------------
GCC--------------------GGGGGCGC----
GTCGCGGTC------------------------------------------------------------------
---------------------------------------------------------------------------
---------------------------------------------------------------------------
---------------------------------------------------------------------------
---------------------------------------------------------------------------
---------------------------------------------------------------------------
---------------------------------------------------------------------------
---------------------------------------------------------------------------
---------------------------------------------------------------------------
--------------------
CTCG-----------------------------------------------------------------------
---------------------------------------------------------------------------
---------------------------------------------------------------------------
---------------------------------------------------------------------------
---------------------------------------------------------------------------
---------------------------------------------------------------------------
-------CGGTCGCGGTCGCGCCCCC-------ACCCTGCCCCAGGTACCTAG----CGCGTTC-
CGGCGCGGAGGTTTAAAGACCCTTCGGGGG------CCT-GCCCGTCCGCCTC--GGGG-TCGGGGG---
CGGATGGGC----CCGT-----------
CGGGGAGCC------------------------------------------------------------------
---------------------------------------------------------------------------
----CCGGCTCCTCC--------AGAC------
CCCGCCCCGGCGTGTCTCTCTCTCTGTCTCTCTCTCTCCCCGTTTCCCCGGTCCCCGGTGTCCGTCTCGTCCCCC
GGTCCGCCGCCGCCCCGCGTCCCTCCCTCGGGGGGGCC-GTCGGGCCGGG---------------------
CTTCGGCCCGTCCCGGTGCGCCTTCCCGCCCGAGAGGGGGGGCCCGCGCTTTCCAGGGCGGTCGGTTTT-
GGGGGGCGGGCCGCGCCGGGGGCCGGCGGGCGAGGAGGAGGAGGAGGGAGGAGGAGAGGAGGCCGTCGGGGGCGG
GGAGCCCCCGC----GGGCGCCTGTGGGGG----TTGACCCCGC--GCCTCGCC---GTGCCCGC-----
GCCG--------CGTGCA------CGG-CGTCGGAC---GTCCGGGCTCAGCCCCCTTG--------
GGGGTGAAA----------CCTCTCCC-GACCCC----------ACCGAGT-------CTGGT--
CTCGTTTCT----CTCGT------
CTGGCCCGGCCCGAGGCACGGGCTCTCTC----------------------------------------------
-----------------------------TCGGGGGAGCCCGGTGTGCCGACGCC-AGCA-ACC-----
TCCG-------------------------------TGGCGACACGGA--------------AAAAACAAAAA---
GTACGACTCTTAG
```

Erinaceus
GGAAGGATCATTAACGCTGTGAATGTTGATGGTGATGATGATGATGGTGAGAGGAGAGCGAGCGGCTCCCGGTGG
TTTGCGCGTTGTGGGGCTGCTCTCCGCTCTCGCTTTGGTTCCGTGGGGGGACCGGTGTGGGGG-
CCGCGCGTCGCGTCGGCGGGGCTCGAGGTGCGAGGGGTTG------------GTCGGGTTCCC-
GGACCCGGCCTCTCTCTCTCTCTCTCTCTCCCGCCCCGTCCCGTGCGCGCGCGT---TCCCCC-
TCCCCCTC---------------------------------
TCGGGGGGGGACCGAGAAGGTGTGCGAGAGGGAAGAGGCCCTCGAGAGGGGGAAGGTCGTCGCGGAGAGGGTTGT
GGGGGGCGCGAGCGGTGTCGGACGGGGGAT------------------------------------
GGTGCCGGTGGTGGGAGGCGGCCAGGGTCCCCGCGGCCGCGGCCCTCCCCGTCG---------------------
----

GAGA-------
CGGCGGTGGTGGTGTCGCGTCTGCGCCCCTGACCCGCC-------------------------------------
CTTCCCTCCCCGGCCCTCTTTCCGTGCCCCTCGTGTTTTCTCCCCGCCTCGCCTCGCCGCTCCCCTCCGCCGCTC
GCTGGCGCTCGCTCGCTCGCTGGCCGGATCGGGTTCCCCGCCCCCCGAATGGCGGGCGGGGAGGA----------
--------------------------------CGGGCGGGC--GGTCGCTGGGG----TTTGTGC-----
TCCCCGCCGCCGCTTCCTCGGCTTTCCCGGGCGGG--

TGTCTCCGTCGTCGCCGCCCCCGCCGCCTTTTCCACACTCTCCAACCTCTCCCCCGGCCTCCCGCGG--------
----

GCGA---

CCGCGCGGCGGCGGCGGGCGGCGGTTGGGTGTGTGTGTTGGGGGGGGGTCGGCGT-----
CGGAGGGCATCCCTCCCCGGTGGCCGGTGTGGCGGTGGGCGGGCCCTGCCCCACGCCGCCCCGTCTCGCTCCGAC
CC-GCCT-GTCCC--------GCCTCGCTCCAGGTACCTAG----CGCGTTC-
CGGCGCGGAGGTTTAAAGACCCCTCGGGGGTG----GTT-GCCCGTCCGCCCT---GGG-TCGGGGG---
CGGTCGGGC----
CCGCAGTCGGGTTCGTTGGGTCTCGCGGCGGCGGTGGTGGCGGTGGCGTCGTCGTCGTGGGGGGGGCGCGCGCAG
GGTGTGGGGCGGGAGGCCACCC--
GGTCTCCCTCTCCCCCGCTCTGCCCGCCCCCCCCACCCCGCGCCTGCCCCCCCACCGCCCCGCTCGCCCCCCCGC
TCCCGCCTGCC--GGGAC------TCCGGCTCTTCTCCGTTCCCTCGCCTGACCGTGCGGTTCC----------
CGGGGTCGGCGGCGTG---------------GCCGCGCCCGGCGCCCC----TCGGGGCGGCT-
GTCACCAGGTGGGGGCG------------CACCTT----
GGTGGCCTTTCCGGTCTCCGGTGGGGTCGCGGTGCTCCGCGCC--------------------------------
---------GACCCTACCCCCGCGTTTCCGCGCGGCGGCGTCGGGT--GACGGGGAAGGG-
CGGGAACCCCCTC----GGGCGCCTGTGGGG--------TTCCGAA--ACCGGGCACGGG--------------
CGTGT---CCCGTGTCTGGGGGAGGG----------------------GCCCC-GTGCC---------

GTGAAA----------CTTCTCCCCGACCCTC---------TTTGAGT-------CTCGTTA--
TGTATATTTTTCTCGTCT----
CT--
---GTC-GGAG-
ACCCGCCGTCCG--------------------------------ACACACGG------
AAAAAACAAAAAACAAAACTC--GTACGACTCTTAG

Monodelphus
 AGAGAGAGCCCGCCGTGCTCTCAGACGCAGCGGGGCCCGTCCCGTCG--TCAGGCCGCGTCCGGTGGAG----
TTTCCCTCCCGTGGGGGGGGGGGCCCGCCGGGC-GGTTTTCTGCGACGGC-
CGCGGGAGAGTCGCGGCGGCGTGTGTGTGCGCCCGC-----GACACC---CGCGGCCGGTTCCT-
TGACCAGCGGGGGTCGGGGGGTGG-TCGTT-GCGGTGTGTCGAGGCGGAGGGGGAAGGTCTCCTTCCCCCGCC-
ACCTCGCGCGCC-TCCGCTTCCCCC-TGCCCTGCGCC-CGATTGCGGCTCGTCCGCCCGCGCCGCCTCCC-
GCGTCGGGGGG-TTGGCTTC--GGCCACG-TGAGGGTGCGGGAGCC-GCCCGGAGGGAAACCAGGCT-
CTAACGC-GAGCGAGGGAGAGCTGCGGC-TTGGGAAGGG-CTCGGCGAGCGGCAGCC-
GGCCCCCCTGCAACCCTGCCTTAGCCCCTCTCC-GGAAGAGACTCC-AGCGGGGCGTCGG-TTTCGGTCCA-
GTGTTTGCGCGCGCCCCTGG-GTCTCGGGCGCCGAGGGCGGCG-CCGCCGGGTCGCGTGCCTCGTGCCGCGG---
CCCCGGGGCACCCG-TCGGGTCCCTTTCCCGTCTTTCCTGCTTCCCGTGCCCCC--GGAGGA-
GTCCCGCCGGGAGGCGAGAGTCCGCCGGCTCACCCCGCCCACACACACGGGTCCCCAGAGCGAGAGGGGCGCCAG
GGGGGTGTGGCTGCCCC-GACCGTGAGGGGTGGCCGGCGG-TGGCCTCCCCGGGCTC-
GACGGGAGCGGGGCTGGGTGTCGGG-
AGGGGGCCGTCCACGGCGGGAGCCTTAGGGCGGTGTCGTTCCCCGGTCCCGGGCTCGAGGCGTCGTGGCGGCTG
CGGCCGACCGGGCGGCGTTCGGTGGGTTCCCTCCGGCGCCGCTCGGCCCCGCCGGTACCCCGGCGGCGTCCCGGA
CGCCGTCGGCGGCCCGTGCGCATGCCGAGTCCCCCCGCGGCGGGGGGTTTTCGAGCGAGAGAGGAATGCTGCTCG
AAGT-
GCTTGACCACGATCCCGCCGCCCCGGCGCGGTCGTGCCCCCGCCGCTGCATGTGCGGGCTTCGGGGGGAGGTGGC
GAAAAGGGGGAGAAGACCCGGCGTCGTCGCGGGCCCC-
ACCCCTCTCCGTGTGGGCGGTTGGGGCCCCGGGTGCCCGGTTCCCCGCCTTCGGTCGTCAAGGTCCCCGCTTTCC
ATGGGGGAGGAAAGGG--
TCGGTGATTCCCTCGGCCGGAGCGGCGGGGCGGGAGCTTAAGGCGGCGGTGCCTCTCCTCCCCTTTGCCCCCTTC
CTCCGCTCCGCCTCAGCCC-GCGGTCGGGT--GTCTTTTAG-GTTCTCCGG-
GTCCGTGCTCGCCGACGCGGCCGAACCGCCTC-GTCCCCACCGGTGGGGCGGGG--
TCGGCGGTGCCGAGGCCCCTGCCCC-GGCGGCTCGAGAGGGGTCC-
CCTCGACGTGTGGGCCAGCGAGGGCCCCGGCGGTCAGGCGCCGGGGTCGGTCCCGCTCGCTCGAGTGAGCGTCTT
C-
CCTCCGAGATGGGGAATTGGGGTTGGCGTGACCGGCCGGGTGTGCCTTGGTCGCTCGTCCCGACCGCCCCGAGGG
GCTGGGGCGAAAACTCCCCCGCTCCTCTCCGCTGTGCGGTCCTAGCCCGCCGTTGGGGCGTGGGAGGCCTTTCCG
CCCCCATCCTGTCCCGGGTACCTAGCGCGTTCTGGCGC--
GGAGGTTTAAAGATTCGGGGGTTGCCCTCCGCTCTCCAGCGGGGTC-----
GGTTCTGTTTTCCCCCCTTCCCCGTCCGCCCTCT---
TCCGACGGTCTCGGGCCCCTCCTCTTGCCGCGTGCCCCGTCCGCGGGTCTCGCGGCTAGAGGTC---
GGGGGTTTGGTGCCCCC---GAGACGGGAAGGGCGGGTCTGGGTGGGGGGGTCT-----
TGGGACGGCCGCCCCCTGGGTTTGCCGGACGGGCCAGTGGGAA-------
GAGGAGATGCCTGGCTCCGACTTCTCCCGTTCTCCGGTGTGGTGCTGGGTTCGGGTCCCGGTCGTGGGGGACCGC
CCGCCCGCCCTCGGTGAGGTGTGCTCCGCGTGGAGGCCGCGTCTCCCCGGTCTTTCGTGCTCCCCTGGC------
----

```
----------------------------------------------------------------------
-
CTTCTGGGGGAGGAAAGCGATCCCGCCCGCCGCGTCGCTCCGTGTCTGGTGGCGTGGGGGGGGAAAGCCTCGTAC
GGCCTGCGGGCCGCGACGAGCTCGGCCGTGGGGTCGAGGGCAGTAAGTAGTCCGGTCATCGGGGGAGGGGGGAGG
ATAGAGCGGAGTGCTCTTTTCCCCCTCTGTGTGCGTGCGTGGGGTGACCGCGGTTCGCGCTGTCGTCGCCCGGA-
TTCGTGTCGGGGGGGGTGCGGTGGCTCGGTGGGGTCGGTGCC---AGGG-
TCCGATCCCCTCCCGCCCCCTGGGAAGGTTGGGGTCCCACTCCCCGGTCTTCTCGGCGCTCGCCCTGGGCCTCGC
CGAACCCCCGTGCGTTTCC--GGTGCG-
GCGCCGTCCGTCCTTGGTCGCGGCCGCCTCGCTCCTCGCTGCACGTGGA-GTGCG-
GGCGAGGACTCTCTGTTTCGA--AACCTCCCCCTGATCCCCGGTGGGCCG--
TCGCGCTGCTTGCTCTCTCTGCCCTCGCTGTCTCGCCGCGTCC-GC-
TTCCGACGGGCTTCCCTGCGCCGTCGCTGGG--GGATGCCCTCCCGGGG--
GCTCCCTCTCGTCGGCCTTCCCTGCGCCGAGGGCCGGCGCCGCGACGAGGCTGGGTCGTCGCTCCTCCCCCTCCG
GTCGGGTTGCCGGTGGCCGGGTTGCCCGCGTGCCCCCCTCCGCGGGGTCGCCGCCGTTGGGTTTGTCGGTGGGTG
CCGTCCCCCGCGTCGGCCCGTCCCGCTTGCCGCCCC-
GGGGACCCCCGGGCGCCTGTGGAGTTTGGCCGCGAGGCCGGCCGTGGCTTCG-CGGGAGCGG-
TTCCCGCGCTGCCTCCCCGGGGACGCCCGGGGAAACGGTCGTTCCTC-
CGAGCCCCCCCACTGCCAAATCGTGTCGTGTCTCGTTTTTTACCTTCCGTTTACGCGTTGTGGGGGTTGCTGTGG
GGTGTTGGGGG--GGGCCACGCG-CCTCCCCCCCC-
TCCCTCCCTCCCGCGTATCCCAAAAAAAACAAAAAATTCG
;
end;
```
