# Supplementary material for: Analysis of Mammalian rDNA Internal Transcribed Spacers
Source: PLoS One. 2013 Nov 19;8(11):e79122. doi: 10.1371/journal.pone.0079122 (PMC3834078; doi:10.1371/journal.pone.0079122)
Supplement: Text S2 — ITS2 Nexus alignment file in TextEdit. (PDF) [file pone.0079122.s002.pdf]

```
#NEXUS
begin data;
        dimensions ntax=36 nchar=3169;
                format
                        gap=-
                        datatype=DNA;
        matrix

Pongo
TCAAT--CGCCCC----CGGGGG-----
TCCCTCT--------------------------------------------------------------------
---------------------------------------------------GGGCTC---TTTGGGGT----
GCA-TGGCT-TGGGGTCC--
CTTGGCAGGGCCCTCC-----------------------------------------------------------
---------------------------------------------
GGGGCCCTCCATCCCCCCAAGTGCAGACC-------AGTGACGTCCGCCCTCCT-CTTCT-GCTATACCC--
GCCCCT-TCCCCC-TCTCCC--
GCAGGCCCTGTGT--------------------------------------------------------------
---------------------------------------------------------------------------
---------------------------------------------------------------------------
---------------------------------------------------------------------------
------------------------------------------AGTC-ACGCGTTGGGTATCGTGGG-
GAAGGGAG---TCTCCCGGCTGT--------GAGGAAGAGAAAGAAGGCAGCACT-----------------
GCCAAAGGTGAAAACAAA-GAGGGAAGACA---------------------GCCGTCTGGGG---
CCGAGT-----TCCC---
GTGGCCAC-------------------------------------------------------------------
---------------------------------------------------------------------------
-----------------------------CACC-ATGGTCCGGGTTCCTCCCTCGG--AGGC---
TCCCTCATGCCTCA----CA----CAG---CTCGTGGTGTGGGGTTC-GTTGGTCCT----GGT-----
GGGGTGGAA-------GGT--CCCATGCC------ATCGTCATT-------GTTGTGCGTCATCAAT---
GGTGGTGGG-GGCTTGTTG------------------GGTGGGGAGGAAAG-CAAGT----------
AAGAATGG-----TCCACCGGG-C-AGAAGGG-----TGGGGCAGCGCGTCCCAGTTGGTGCC-GGTTT-
GCACCCG--------------CCCCCTGG-TGG--------
GAACAGT---------------------------------------------
GACCAGTCGATGTCAATCCAGGCCACCCTTGCTCGTAATAGCGT----CAAGGCAC-----
GTCCTTCTTAATCCAGCC-TGACCT-----------GCCCACCCAAACCGC-----------TCG-
TTCACGGCGC------GTCCGGGCC--------------GGAAGCCC---GCCCC--GCGGCCCG------
CCCGGCCGCGCTCGCGGCCGCGGTCCC------GGGTT----CGCGT-GCCCCCGGCGG-TGAC-CCGC-
GG-----GACGCC--GCGGTGTC-GTCCGCCGTC---------GCGCGC---------------
CCGCCTCCGGCTC--------------GCGGACGCG-CCGTGCCG-TGCCGGG-G-----
CCCCGTCCCGGGCTTCCG-CGT----
CGGGGCGGGTGT---------------------------------------------------------------
---------------------------------------------------------------------------
---------------------------------------------------------------------------
---------------------------------------------------------------------------
---------------------------------------------------------------------------
```

---GCGGCGTCCGTCCGTCCGTCCGTCCCGCCCC------CGGCCCGTGCCCC--TCC-CTCCC----GTC-
GTCCCGCCCGC--

-------------TCCGGCGGGGCGGGGCGG--CGGGGGTGCCGTCGGCCC-GCG-----------
GCTCCCTCT-TC-TCGCCTCTCCCT------------------TCGC---CGGGC-----------
CCGTCTCCGGAC-GGGGCGTC-GGG---------------
CGGGCCGGCGC--

--GTTCCGTCC----------GGCCGCCCCCGCCCGTTC--GCGC----GCCTTCCCC-------
TCCGAGAC---GC-GACCTCAGATCAG

Gorilla
TCAAT--CGCCCC----CGGGGG-----
TGCCTCC--
---GGGCTC---CTCGGGGT----
GCG-CGGCT-GGGGGTTC--
CCTCGCAGGGCCCGCC---

GGGGCCCTCCGTCCCCCTAAGCGCAGACC------CGGCGACGTCCGCCCTCCT-CTTCGTGCCGCGCCC--
GCCCCT-TCCCCC-
TCCCCCCCGCGGGCCCTGCGC--

--GGTC-ACGCGTCGGGTGGCGCGGG-
GAAGGGGGGGGGCGCCCGGCTGT--GAGAGAGAGAGAGAGGGAGGGCGGCGCCG-----------
CCGCCCGCAAAGACGGAGAGGGC----AAGAGAGAG--------------------GCCGGCTCGGG---
CGGAGA-----TCCC---
GTGGCCGC---

-----------------------------CACC-GCGGTCCGGGTTCCTCCCTCGG--GGGC---
TCCCTCGCGCCGCA----CG----CGG---CTCGGGGTTCGGGGTTC-GTCGGCCCC----GGC-----
CGGGTGGAA-------CGT--CCCGTGCCC-----GTCGTCGTC-------GTCGCGCGTCGTC------
GGCGGTGGGGGGCGTGTTGCCTGTGGT-----GGTCGGCGGGGGAGGAAGG-CGGGTCC-GGA--
GGGAAGGGTT------CCGGCGGGGAGAGAGGGCG----TGGGGGAGCGCGTCCCGGTCGCCGC--
GGTTCGCCGCCCG--------------CCCC-TGG-TGGC--GGCCC--
GGCGTTC---------------------------------------GGCCGACCG-CCGCTCCCCCGCC---
CCTCCTCCTCCCCGCCGCCCCTCCTCCGAGGCCCC----GT-
CTCCCCCTCGCCCTACCGCACGCACGCACGCCCGCCCGCCCGCCCGGC-----------
TCGCCTCGTGGCGCGTG---GTCCGGGGCC-------------GGAAGCCC---GCCCC--GCGGCCCG------
CCCGGCCGCGCCCGTGGCCGCGGTCCC-----GGGGTT----CGCGT-GTCCCCGGCGG-CGAC-CCGC-
GG-----GACGCC--GCGGTGTC-GTCCGCCGTC---------GCGCGC---------------
CCGCCTCCGGCTC--------------GCGGCCGCG-CCGCGCCG-TGCCGGG-A-----

CCCCGTCCCGTGCTTCCG-CGT----CGGGGCGGGG---CGGCTCC-GCCACCGCC------------
GCCGCGTCC-TCGGACCCGTCCCCCGCGGC-
CTCC---

------------------------------GCGGGGGAAGGGTCGG----------------
GGTGT-------------GCGGTGCC------------CGTCCCGCCCC------CGGCCCGTGCCCC--TCC-
CTCCG----GTC-
GTCCCGC--

-------------TCCGGCGGGGCGGCGC-----GGGGGTGCCGTCGGCC--GCG-----------
GCTCTCTCTCCCGCCGCCTCTCCCC------------------TCGC---CGGGC-----------
CCGTCTCCCGAC-GGAGCGTC-GGGCG--GGCGGGCCGGT-GGG----
CGCGATTC---

--CGTCCG-
TCCGTCC--GCCCGCCCGTC---------------------GACCCCGTTCCCC-------TCCGAGAC---GC-
GACCTCAGATCAG

Homo
TCAAT--CGCCCC----CGGGGG-----
TGCCTCC--
---GGGCTC---CTCGGGGT----
GCG-CGGCT-GGGGGTTC--
CCTCGCAGGGCCCGCC---

GGGGGCCCTCCGTCCCCCTAAGCGCAGACC------CGGCGGCGTCCGCCCTCCT-CTTGCCGCCGCGCCC--
GCCCCT-TCCCCC-TCCCC-
CCGCGGGCCCTGCGT--

--GGTC-
ACGCGTCGGGTGGCGGGGGGGAGAGGGGGGCGCGCCCGGCTGAGA----------
GAGACGGGGAGGGCGGCGCCGCCG--------CCGCCCGCGAAGACGGAGAGGGA----
AAGAGAGA---------------------GCCGGCTCGGG---CCGAGT-----TCCC---
GTGGCCGC---

-----------------------------CGCCTGCGGTCCGGGTTCCTCCCTCGG-GGGGC---
TCCCTCGCGCCGCG----CG----CGG---CTCGGGGTTCGGGGTTC-GTCGGCCCC----GGC-----
CGGGTGGAA-------GGT--CCCGTGCCC-----GTCGTCGTCGTCGTC-GTCGCGCGTCGTC------
GGCGGTGGG-GGCGTGTTGCGTGCGGTGTGGTGGTGGGGGAGG-AGGAAGG-CGGGTCC-
GGAAGGGGAAGGGTG------CCGGCGGGG--AGAGAGGG---TCGGGGGAGCGCGTCCCGGTCGCCGC--
GGTTCGCCGCCCG--------------CCCC-CGG-TGGC--GGCCC--

GGCGTCC---------------------------------------GGCCGACCG-CCGCTCCCGCGCC---
CCTCCTCCTCCCCGCCGCCCCTCCTCCGAGGCCCCGCCCGTCCTCC---TCGCCCTCCCCG-CGC--
GTACGCGCGCCCGCCCGCCCGGC-----------TCGCCTCGCGGCGCGTC---
GGCCGGGGCC-------------GGGAGCCC---GCCCC--GCGGCCCG------
CCCGGCCGCGCCCGTGGCCGCGGCGCC-----GGGGTT----CGCGT-GTCCCCGGCGG-CGAC-CCGC-
GG-----GACGCC--GCGGTGTC-GTCCGCCGTC---------GCGCGC---------------
CCGCCTCCGGCTC--------------GCGGCCGCG-CCGCGCCG-CGCCGGG-G-----
CCCCGTCCCGAGCTTCCG-CGT----CGGGGCGGGG---CGGCTCC-GCC------------------
GCCGCGTCC-TCGGACCCGTCCCCCC-GAC-
CTCC---

---------------------------GC-GGGGGAGACGGGTCGG----------------
GGCGT-------------GCGGCGCC------------CGTCCCGCCCC------CGGCCCGTGCCCC--TCC-
CTCCG----GTC-
GTCCCGC--

-------------TCCGGCGGGGCGGCGC-----GGGGGTGCCGCCGGCC--GCGC----------
GCTCTCTCTCCCGTCGCCTCTCCCCC-----------------TCGC---CGGGC-----------
CCGTCTCCCGAC-GGAGCGTC-GGG------CGGGC-
GGTCGGGCCGGCGCGA---

-TTCCG-TCCGTCC--GTCCGCC-GAGC--------------------GGCCC-GT-CCCCC------
TCCGAGAC---GC-GACCTCAGATCAG

PanA

TCAAT--CGCCCCCC--CGGGGG-----
TGCCTCC--
---GGGCTC---CTCGGGGT----
GCG-CGGCT-GGGGGTTC--
CCTCGCAGGGCCCGCC---

GGGGCCCTCCGTCCCCCTAAGCGCAGACC------CGGCGACGTCCGCCCTCCT-CTTCCCGCCGCGCCC--
GCCCCT-CCCCCC-TCCCC-
CCGCGGGCCCTGCGT--

--GGTC-
ACGCGTCGGGTGGAGGGGGGAGGGGGG----
GCGCCCGGTCGAGAGCGAGAGAGAGAGAGAGGGAGGGCGGCGCCG-----------
CCGCCCGCGAAGACGGAGAGGGG----AAGAGAGA---------------------GCCGGCTCGGG---
CCGAGT-----TCCC---

GTGGCCGC---

-----------------------------CACCTGCGGTCCGGGTTCCTCCCTCGG--GGGC---
TCCCTCGCGCCGCA----CG----CAG---CTCGGGGTCCGGGTTC-GTCGGCCCC----GGC-----
CGGGTGGAA-------GGT--CCCGTGCCC-----GTCGCCGTC-------GTC-------GTC------
GGCGGTGGG-GGCGTGTTGCGTG-----TGGTGGCGGGGGTGGGAGGAAGG-CGGGTCC-GGA--
GGGAAGGGGGT-----CTGGCGGGGAGAGAGAGGG----TGGGGGAGCGCGTCCCGGTCGCCGC--
GGTTCGCCGCCCG--------------CCCC-TGG-TGGC--GGCCC--
GGCGTCC---------------------------------------GACCGAACAA-
CGCTCCCGCGCCCCCCCTCCTCCTCCACGCCGCCCCTCCTCCGGGGCCCC----GTCCTCC---
TCGCCCTCCC--------GCACGCGCGCCCGCCCGCCCGGC-----------TCGCCTCGCGGCGTC---
GTCCGGGGCC-------------GGAAGCCC---GCCCC--GCGGCCCG------
CCCGGCCGCGCCCGTGGCCGCGGTCCC-----GGGGTT----CGCGT-GTCCCCGGCGG-CGAC-CCGC-
GG-----GACGCC--GCGGTGTC-GTCCGCCGTC---------GCGCGC---------------
CCGCCCCCGGCTC--------------GCGGCCGCG-CCGCGCTG-TGCCGGG-G-----
CCCCGTCCCGAGCTTCCG-CGT----CGGGGCGGGG---CGGCTCC-GCC------------------
GCCGCGTCC-TCGGACCCGTCCCC--GGAC-
CTCC---

------------------------------GCGGGGGACGGGTCGG----------------
GGTGTGT-----------GCGGTGCC------------CGTCCCGCCCC------CGGCCCGTGCCCC--TCC-
CTCCG----GTC-
GTCCCGC--

-------------TCCGGCGGGGCGGCGC-----GGGGGTGCCGTCGGCC--GCG-----------
GCTCTCTCTCCCGTCGCCTCTCCCC------------------TCGC---CGGGC-----------
CCGTCTCTCGAC-GGGGCGGC-GGG------CGGTC-
GGTCGGGCCGGCGCGA---

-TGCCG-TCCGTCCGCGTCCGCC-GAGCGGCCCGTCCCCGCCC--GCCGGCCCCGTTCCCCC------
TCCGAGAC---GC-GACCTCAGATCAG

PanB
TCAAT--CGCCCC----CGGGGA-----
TGCTTCC--
---AGGATC---CATGGGAT----
GCA-TGGCT-GGTGGTTC--
CCTCGCAGGGCCCGCC---

AGGGCCCTCTGTCCCCTCAAGTGCAGACC------CAGTGACGTCTGCCCTCCC-CTTCT-GTCATGCCC--
GCCCCT-TCCGCC-TGCCCCT-
GTGGGCCCTGCAT--

--GGTC-ACCTGTTGGGTGGCAGGGG-
GAAGGGG------GCCCGGCTGG----------GAGAGAGACAGCGGGTGGCGCTG-----------
CCGCCTGCGAAGATGGAGA-GGG----AAGAGA-----------------------GCCGGCTCTGG---
CTGAGT-----TCCT---
GTGGCT---

---------------------------GCCAC-TGCAGTCTGGGTTCCTCC--GAAGGGGGC---
TCCCTTGTGCCGCA----CC----TGGC--TT-GGGGTTTGGGGTTT-GTCGGCCCT----GGC-----
CGGGTGGAA-------GGT--CCCGTGCCC-----ATCGTTGTTGTG-------------CGTT---------
GGTGGG-GGCGTGTTGCAT--------------GTGGGGGGAGGAAGG-CAGGCCC-GGAA--
GGAAGGGAT----------CGGG---------------TGGGGGAGTGTGTCCCGGTTGCTGC--
AGCTTGCCACCTG--------------CCCC-TGG-TGGC--GGCCT--GGCT---
GG---------------------------------CATCCGGTGACTG-CCGCTCCCATGCC---
CCTCCTCCTCCTCGCCACCCCTAATCCGAGGCCCC----GTCCTCC---
TCGCCCT--------------------CCCACCTGCCCAGC-----------TCGCTTTGTGGTGCGTT---
TTCTGGGGCT-------------GGAAGCCC---ACCCC--ATGGCCCG------CCTGTTTG-
GCTCATGGCTGTTATCCC-----GGGGTT----TGCCTC-TCCCCGGCGG-TGAC-CTGC-GG-----
GATGCC--GCGGTGTT-GTCCGCTGTC---------
GCG--TGGCCGCG-CTGTGCCT-TGCCGGG-
G-----CCCCGTCCTGAGCTTCTG-CGT----TGGGGTGGGG---TAGCTCC-GCCACC---------------
TCTGCGTCC-TTGGACCTGTCCCCC---ACA-
TCTGTGTG---

--------------------------------GGGGACGGTCGG-----------------
GGTGT-------------GCCGTGTC------------TGTCCCACCC-------------CGTGCCCC--TCC-
CTCCC----GTC-
TTCCTGC--

-------------TCTGGCGGGGTGGC-------GGGGGTGCCATCGGCTGTGAT-----------
GCTCTCTCTCTT---GCCTCTCCCC------------------TTGC---TGGGC------------
CTTCTCCTGAC-GGAGCACC-GGG------TGGTCGGGT-GGGCTGGC-
TGGTGCGTTCC--

---G-
TCCATCC--GCC-----GACCAGCCCACCCCCGCCC-TGCCCTGCCCGTTTGCCTCCTTCCCCTGAGAC---TT-
GACCTCAGATCAG

Macaw
TCAAT--CGCCCC----CGGGGG-----
TGCCTCC--

---GGGCTC---CTCGGGGT----
GCG-CGGCT-GGGGGTTG--
CCTCGCAGGGCCCGCC---

GGGGCCCTCCGTCCCCCCAAGCGCAGACC------CGGCGACGTCCGCCCTCCC-CTTCC-GCCGCGCCC--
GCACCTTTCCCCC-TGCCC-
CCGC---

--GGTC-ACGCGTTGGGTGG--
GGGGGGAAGGGGGG----GCCCGGCT------GGGAGACCGGAGAAGGGAGGGCGGCGCCG-----------
CCGCCCGCGAAGAGGGAGAGGG-----AAGAGAGA---------------------GCCGTCTCGGT---
CCGCGT-----TCCC---
GCGGCCGCGGCC---

------------------------GCCGCCGCC-GCGGCCCGGGTTCCTCCCTCGGGGGGGC---
TCCCTCGCGCCGCA----CG----CGG---CTCGGGGTGCGGGGTTC-GTTGGCCCG----GGC-----
CGGGTGGAA-------GGT--CCCGTGCC------GCCGTC----------GTCGCGC---
GTCGGCGGCGGCGGTGGG-GGCGTGTGTCGT--------GGGGGTGGGGGGGAAGGAAGGGCGAGGTC-
GGA------GGGGTTGC-------GCGGGGAG--AGGTCC------GGGGAGCGCGTCCCGGTCGCCGC--
GGTTCGCCGCCCG--------------CCCC-TGG-TGGC--GGCCC--
GGCGTCC---------------------------------------GGCCGACCG-CCGCTCCCGCGCCG--
CCTCCTCCCCGCCGCCGCCGC---TCCGCACCGCCACC-GTCCTCCT----
GTCCTCCCC-------------------GCCCGCCCGGC-----------TCGCTCC---GCGC------
GTCAGGGGCC-------------GGAAGCCC---GCCCC--GCGGCCCG------
CCCGGCCGCGCTCGTGGCCGCGTTCCC-----GGGGTT----TGCGT-GCCCCCGGCGG-TGAC-CAGC-
GG-----GACGCC--GCGGCGTC-GTCCGCCGTC---------GCGCGC---------------
CCGCCTCCGGTCC--------------GCGGCCGCG-TGGTGCCG-CGCCGGG-G-----
CCCCGTCCCGAGCTTCCG-CGT----CGGGGC-GGGTCG-GGCGCC-
GCCGCCCGCTGGCCGCCGCCCGCCGCCTCC-TCGGGCTCGTCCCCC---AC-
CTCCAC---

GGGGGGGGGGGGACGGTCGGGGGGGTCGGTGGGCGGGGGTGTGTGGCGGTGGTGCGCGGCGCC------------
CGTCCCGTCCC------CGGTCCGTGCCCC--TCC-CTCCC----GTC-
GTCC---

--------------CCGGCGGGGCGGCG------GGGGGCGCCGTCGGCC--GCG-----------
GCTCTCTCTCTCTC-GTCTTCTCCCC-----------------TCGC---CGGGC-----------
CCGTCTCCCGAC-GGAGCGTCCGGGCGC-GGCGGGACGGC-
GGGCCGGCGCGG---

CGTTCCG-CC--GCC-----GACCCGCCCACCCCGCCCGTGC------GCCTCCCGCCC----TCCGAGAC---
GC-GACCTCAGATCAG

Nomascus
TCAAT--CGCCCCC---CGGGGGG----
TGCCTCC---
--GGGCCC---CTCGGG-----
CGCG-CGGCT-GGGGGTTC--
CCTCGCAGGGCCCGCC--

GGGGTCCTCCGTCCCCCAAGCGCAGACC------CGGCGACGTCCGTCCTCCC-GTTCC-GCCGCGCC--
GGCCCCT-TCCCCC-TCCCC-
CCGCGGGCCCTGCGC---

GGTCGACGCGTGGGGTGGCGGGGGGGAGGGGGG----GCCCGGCC---------
GGGAGAAGGGAGGGAGGGCGGCGCCG----------CCGCCCGCGAAGGCGGAGAGGGG---
GAAGAGAGAGAA-----------------GCCGGCTCGGG---CCGGG------TCCCC--
GTGGCCGC--

----------------------------CGAC-GCGGTCCGGGTTCCCCCTCGG--GGGC---
TCCCTCGCGCCGCA----CG----CGG---CTCGGGGCTCGGGGTTC-GTTGGCCCT----GGCCGGC-
CGGGTGGAA-------GGT--CCCGCGCC------GT-GCCGTC-------GTCGCGCGTCGTC------
GGCGGCGGG-GGC------------------------------GGGAAGG-CGAGCCCCGGAA------
GGGT-------CTGGCGGGGAAGAGGGTCCGGGGTAGGGGAGCGCGTCGCGGTCGCCGC--
GATTTGCCGCCCG-------------CCCC-TGG-TGGC--GGCTC--
GGCGGCC---------------------------------------GGCCGACCG-CCACTCCCGTGCC---
CCTCCTGCTCCCGGCCACCCC---TCCGAGGCAC-ACC-
GTCCTCCCC-------------------------------GCCTGCCCGGC----------
TCGCTTCGCGGTGC------GTCTGGGCC-------------GGAAGCCTGCCGCCCC--ACGGCCTG------
CCCAGCCACACTCGCGGCCGCAGTCCC-----GGGGTT----CGCGT-GTCCCCAGCGG-CGAC-CCGT-
GG-----GACGCC--ACGGTGTC-GTCCGACGTC--------GCGTGC---------------
CCGCCTCCGGCTC-------------GCGGCTGCG-CCGTGCTG-TGCCGGG-G-----
CCCCGTCCCGAGCTTCCA-CAT----CGGGGTGGGG---CAGCGCC-GCCACCGCC------------
GCAGTGTCC-TCGGGCCCATCCCCGCCCGC-
CTCC--

--------------------------GCGCGTGGGGACGGTCGG-----------------
GGCGTGCGC-------GTGCGGCGTC-----------CACCCCGCCCC------CGGCCCGTGCCCC--TCC-
CTCCC----GTC-
GTCCCGC---

-------------TCTGGTGGGGCGGA-------GGGGGTGCCGTTGGCCCCGCG-----------
GCTCTCTCCCTCT-GGCCTCTCCCC------------------TCGC---CGGGC-----------
CCGTCTCTCGACGGGAGCGTCCGGGCAGGGGCGGG--GGC-
AGGCCGGCGCGTT--

CGTTCCGTCT--GCCGCT--GGCCCGCCCGGCCCTCTGCCTGTCGGCCCCATTCCCC-------TCCGAGAC---
GC-GACCTCAGATCAG

Microcebus
TCAAT--CGCCCCC---CGGGG-
TGTGTTGCCTCC---

GGGCTCCACCCCGGGGT---CGCG-CGGCT-GGGGGTATC-
TCTCGCAGGGCCCCGCTCC--

GTGGGCCCTCCGTCCCCTTAAGTGCAGACCT-----TGGCGGGGCGCGCCCCACCTCTTCC-GCCCGACC---
GCCCC---------------
ACGTAGGCCCGGCG---

TTGGCGCGTGGGGCGGGGGG------------------------------------
TGCGGTGAGGTCGCGACCCGCC--------------TCCCGCGATGAGAGAAGAGAGGAC---
CGGGAGG--------------------GTC-GCTCGCG---CCGAGCTTGC-
TCCCCTCGTGGCC--

--------------------------------------GCCACGGTGAC-GCTCCT--CTCGG--GAGC--
CTGCCTCGCGC--TG----TG---CCGG----CCGGGCCT--------TCCCCCC------CTCTC---------
TGGGGGGGGTCGTCCGG-----------------------GAAGGGCCCGACGGGGTTCCCGCGCCC----
GTCGCGCGCCGGCGAGCCTCGGCCGGTGGGCGCCGGGGGGCCGGGGGCAAGGGGGGGCGT----
CCGGGGGGTCTGGCGCG-----------
CGTGAGGGGTTGGTCGCGCCCGGGTCGCC--------------------------------GTCG-------
TCCGCCGTCCGGCGGGCCCG---
--------GCGGGCCGGCCCGGCTTTTTACCCCCC---
ACGCCGCCTCCCTCTCTCGTCTCCCCCCCACCTCCGTCTTCCCC---C--------GGCG----------
TCG---CCGACCGAGCGCTCCGCCGGGCGC-----------GCGGAGGGG-------
CCGGGTGGCCGCCCCCGTCGCGTTGCCCGT-TCGGGGCCGCTCCCC---GGGGGC-----TGCGCCGCCAC-
GGCGG-CGAC-CCGC-GG-----GACGCC--GCGGCGTC-GTCCGCCGAC---------
GCGCGC---------------CCGCACCGGGGTCCCC-----------GCGGCC----CGCCGCCG----CGTA-
G------CGCGTCCCGAGCCCGGTTG------CGGGGCCGGGT--CGGCGCGGTTA------------------
GCCGCGTC--
TCGGCCGTCTCCCCCCGCCCCGGGGGGTGGGCCGCCGCGTCGACG------------------------------

----
CCCGGGGAGGGGACGGGGAGGAAGAG-GGAGCG----
TTC--

CGCTCCCTCCCCTCCTCCGCGCCTTCCCTCCCCGGACGCGTTCGCGGTGCCGCCCCCCCCCGGGGGTC-
GCGGTGCGGTCGAGGGGC---GGTTCT---CTCGCTGCCGCCGG----------CCCCGGGTCC-GCGCC--
TCGCCGGTCCGTGC-----TCC-CTCCC----GTC-
GTCCCCGCGGCCTCCGACGGCGCGGGCGCGCCCTCCTTCCCTCCTCCCCGGCCCGCCCCCGCG------------
----
GTCGT----------------------------------
CGCGGGTGACGCGGCGCCGGGGAGAGCGGTGTCGGGAGGGGGCGCCTCGCGTCGGAGGGGGCCGGCCTGGGGGC
GGCGGTCGACCGTGGCCGGCG--------TCCCTCCGTCGCGCGCGAGCGGGCC-----GCTCTCCC-------
GCCTCTCCTCCCTCGCCTTG--------TCGC---CGAGCGCC------------------CGGGCGCGC--
GCCGCGCG---

--------------------CGTT---------------GCGCCCGC----------------------
GCCTCCCC-------TCCGAGAC---GC-GACCTCAGATCAG

Otolemur
TCAAT--
CGCCCCTC---

--------CGGGGT----GCG-CGGCT-GGGGGTTGC-
CCTCGCAGGGCCCCGAGA---

CGGGTCCTCCGTCCCCCTAAGTGCAGACCTC----TGGCG--GTGCCCTCCGCCGCTTCC-GTCCC------
GGCCC---------TCCC----
GTGAGCCC---

--------------------------------AGT-------GGTGGACC------
GTGGGGGG------------------------------------TGGGGCGTGGCGGGGCGGCGCTG-
CC----------GCCCGCGAGCGGGGGCAGGA-------GAGAG----------------------------
ACGGG-----GGGCTT---TCCC---
GCGG---

----------------------------TCTCTCC-GGTCC--GTTT----CTCGG--GAGC---
CGCCTCGTGCCGCG----CG----CGG----ACGGGGCCGCACG-CCGCCACCCTCCGC-TAAC--GCGGGG-
GTGGGGGTTTGGCCTT---T---GCCGCCTGGGG---------CGGGCCCGGCGGGT--
CCCGCGTCCGTCCGTCGCGTCGCGTCGCGTCGCGCGGTGAGGCGGGGGTGGGGTCGTGGCCGGCGTCGGCGTCGT
CGTCGGGGGTCTTTGA--
GAGTCGTGAGGTTGGGGGAGGCGCGGGGGGGCCGGGTCGCGGCGGTTTCGCCGCCAGCCCC------------
CGG-TGG-CGGCTG-------GGGC--
GACCCGGCCGGTC-------------------------------

CTCCCTCCCCTGCTCCCCTCTCTCCCCGTCGCGC----------GTCTTCC-----GTCGCGCCGC-
ACCCCGCCCCCCC----TCGGCCGCCGTGCGCCGTCGGGGC------------GCGGGAGACAACCC--
GCC------------------GTCGCCCGCGT-CGGGGCCGCGCCCCC--GGGGC-----CGCGT-GTCCC-
GGCGG-CGA-CCCGC-GG-----GACGCC--GCGGCGTC-GTCCGCCGTC---------
GCGCGC---------------CAGCCCTGGTGTC--------------GCGGGCGCG-CCGGGCCTC-
TCCGGG------------CCCCGACCTCCCCGGGC---
GCGGGGGCCGGAGAGTTTGGCGGTGTGAGCGGCCCGGG-----
GCCGGGCCGGGCCGGGGATGGCGGCGCGCGTGTTCG---------------------------------------
----
----------------------------CGGG---------------
GCCCGGGTGTCGGG---
TCCGTCGCGGCGGTGGTCGGGGGCCGCCGGCGGTCGCGGGGGCTGACCGCCGGCTCAAGTTCCCCCTCTGCCGTC
CGCGGCCCCACCCTCCCCGTCGACGCCGCGCGGTCCCCCCTCCGTGGGCCCGGGCCCGGGCGCGGGCGCTCCGCT
CTCGCTCTCC-GGCTC-GCG----CCGCCGGCTCGTGCTCC--GTCGCTGCC----GTCTCC-
GCGACCCCCTCGGTTGCCTCTCCGCCCGTCTCCGGC-----CGGCCGCGCGCACGC-
TCTCCGC---
GTCGC------------------------GCGCGTGTTGTGCGGGG-
CGGCGTCGGCGGCGGCGGCGT----------------------
TTGCCGGGGCCGCGGGGTCGGCGGT------------------GTTCGACTGCGGTGCAGAAAG-----
GCG-----------GCCCCTTCTCTC--GGTTCCCCGTTTC----------------TCGCTC-TGGGC-
C--------------------CGGGGC-----GGGCGGGC-------GGGCGGGCGTGT--GGGCGG-
GGCGGTC-
GAGGCTCC---

--GGCTGTCGCCGCCC---
CGCCCCACCCTCCCCTCAACCGGCCCCGTGGTCTCCCC-------TTCGAGAC---GC-GACCTCAGATCAG

Tupaia
TCAAT--
CGCCCCCG---

-------TGGGGGT----GCG-CGGCT-GGGGGTTC--
CCTCGCAGGGCCCGCC---

TCGGGCCCTTCGTCCACACAAGTGCAGAACT-----TGGCGCCTTCCGGCCCC--CCCTCC-GCCC-
ACCATCCGCACCCCTCC---TGCCCTC--
CGGTCTGTCCACCC---

--------------------------------GT--GGGCAGCCGTGTTGGTGGGGG--------
GTGGGGCAG-----GCCGGTGGC--------GTGGGGGGTTGGTCGGATTGGCGCGCGTC---------
CCCGCGCGAGAGAGGAGGCGAGGGAGAGCCGGCCGTCC--CCGTCGGGGAA----GGGGGCTCGCG---
CCGAGC-----TCCC---
GTTG---

--------------------------ACCGTGCCACGGTC-------TCCCTTCGG--GGGC---
GCCCTCGTGCCGCA----CA----CGGTCCCTTGGGC-------CGGGTCCCACCCC---CGCTCGGGCAT-
GGGTGGGGCGCGCC--GT--CCCGTGAATTC--------------------GCGCGTCGCGGT----
GGCGGGGTCGCGCGGTTGTGGTGTGAGCGGTGTGTGGGGGAGGAGCCG--CGGACGGCCGGGA------
TCGGGGCGCTTCCCGTGGAGTCGCCTCCGCGCCCCCGG-------------------------------------
---------------CGGC----------
GGCCCTCGGCCGTCCCGTGTCCC------------------------------
GGTCGACCTTCTTCCCCCCCGCCTTTCCTCCTC-------
CCACCCGC--GTCTCCCCC---
GCTCCGCTTC----GGCGTTCGCGGGCACC-GGCGCGTCCCTCGCCCGGGCG-----
GCCGCGCTCCCGAGGCTGGCTCGTGCCGTGGCCCG---------GTCGC--GCGC--GCGGCCGTGCCC----
GGAGCG---ACGCGT-GCCCC-GGCGG-CGGC-CCGC-GG-----GAAGCC--GCGGTGTC-
GTCCGCCGAC---------GCGCGC---------------CGTCCCCCGGCGGCCTT------------
GGCCGCGTTGGCGCCG-TGC---------------GCCCCGAGCCCCCGGCGC--
GGTGGGGGGCCTCTCGGCGGGTTGGCG--------CGGGGGCCTTTCCGTGTCCGGCGGCCGC-
TCGCCCCC----
CTCCC--

CGGGGG---

TGTGCCGTCGGTCGGCCGGTCGGCCCCTCGTTTCCCGTCGAGGTGCTCGCTCCCCCCC--GC-CCGCGG---
CCGCCGGCCCGTGCCCCGCGTCGGTCC-----
GCTCCCTCCTCCGTCCCCCCCCACCTTTCGCCCCCGCCGTCTCTCTCTCTCCCCACCGTGTCTCTCCGCAC----
---CGGTCGC-
GTGCGGGTGGTGGGGCGGTGCGGACGCGCGTACCGCGGGCGTTTCGGTGTCGGGGGGGAGGTCACGGAGAGCGGG
GCGGCGGTGGAGCC--------GTGCGAGCCGGCGCGTCGGGCCCCCTGCCTCTCTACC--CCGTGAGGGT--
GTGCGCC------------G-TCCTCCCTCCCT---CTCTCCCTCGCC--------------TCGCC--
CGGGC--CCGGTTCGGGCGTCCGGGAACGGAGGTGTC-
GCCGTGTGGCCGACGGAAGGTCCGTCCGCGAGGGGTCCGCGTCCCGGCGTGTCTTGTCGTGTTGCGTTCCCGGGC
TCGGCTTCGGGGGCCGGTGTGTGTGGGGCCGGCTCGGTCGGGGCCGCGGGGGGTTTCCC-GTC-
GGGTCCCCCCGCCTCTTCCCCCGCCCGCGCCGCCTTTCCTCCCTCCGTGTCCCCCGAGCCGCCTGTCCCGTTGGC
GCGTGCACGCGC-TCCTGGCTCGCGCCCGCCCCTCCGCGCCCGTCCGTCC-
GGCCGCCGCCGGCGCCGCCCGTCCCGGCGC-TCCCCCC--CTCTCCGAGAC---GC-GACCTCAGATCAG

Rat
TCAAT--CGCGCCCCCCTT----
GCGGTTTCGCC--

GCCGCGGGGGGGATCGCGT---CGCG-CGGCT-GGGAGTCT--
GCTCGCAGGGCCCGCTCCC--

CGGGGCCCTCCGTCTCCCGAAGTTCCGACGT-----GGGCGGCGTCCGGCGTTCGCC-CGC-
GCCCCGTGGGTCCGCGCGGGCTCGTCCTCCCTGCCCGCCGTCGTC------
GTGCGGCCGGGCGGCCCTCCT-------CGCGGCTCCTTCCCCGCGCTCCGCC-
GCTCCCGCTCCCGT--------------------CCTCGC-------------------

```
CCGCTC-------------------TCCCCCG--
CCGTGGGAC------------------------------------------------------------------
----------------------------------------
GCGTCGGCGGGGCCGGTCGGGTGCTGGGCGCGGAGAGGGGGGGGAAAGTCGCGTCGCCGGGGTCGTTCGCCGCGC
CTCCGGTGGGGTGGGGGGC-CGGGA--CCCCGCCCGCGGGGCCGGCGC-GTCCGCCGCCGCCGCGCGCCC--
GCCCGGTGCGTGCGGAGAGAGAGAGAGAGAGAGAGAGACCCGCGAGGGTCGTTCG--------
GGGGGGCCGTA----------------------------------------------------------------
---------------------------------------------------------------------------
---------------------------------------------------------------------------
---------------------------------------------------------------------------
---------------------------------------------------------------------------
---------------------------------------------------------------------------
---------------------------------------------------------------------------
---------------------------------------------------------------------------
---------------------------------------------------------------------------
---------------------------------------------------------------------------
------------------------------CCGCGCCCGCGGGGCC---GGTC---TCGCGTCCCTCTCCT-
CCCCCGC--TCCCTCTACC-----GGGGC-----CGACC-GCTCC-GGCGC-CGGC-CCGC-GG-------
CGCC--GCGGCGTCCGTGCGCCGAC---------GCGACTT----------------
GCCCCCGGGATGTGGGGCCGGCCGGCGAAGGAGAGG-TCGCGGGCT--------------------
CCGGCCTGGC-----------------------------------------------------------------
---------------------------------------------------------------------------
---------------------------------------------------------------------------
---------------------------------------------------------------------------
---------------------------------------------------------------------------
---------------------------------------------------------------------------
---------------------------------------------------------------------------
---------------------------------------------------------------------------
---------------------------------------------------------------------------
---------------------------------------------------------------------------
------------------------TCCCCCGGCGCCCTCGCCC----------CGTCGTCCCCG------
TTCTC------GCTCTCGCTCTC-----CTCTCCTCTCCTCTCCTTCCG-----TCGCC-
CGCGCGCGCCC----------------------------------------------------------------
---------------------------------------------------------------------------
---------------------------------------------------------------------------
---------------------------------------------------------------------------
---------------------------ACCTCTCCTCCTTCTCCTCCTCTGGACC---GC-GACCTCAGATCAG
```

Mouse

```
TCAAT--CGCGTC---ACCC---GC---
TGC------------------------------------------------------------------------
-------------------------------------------------------------GGTGGGT-
GCTGCG-CGGCT-GGGAGTTT--
GCTCGCAGGGCCAACCCCCC-------------------------------------------------------
------------------------------------
AACCCGGGTCGGGCCCTCCGTCTCCCGAAGTTCAGACGT---GTGGGCGGTTGTCGGTGTGGCGCGCGC-
```

GCCC-------
GCGCGCGAGCCTGGTCTCCCCGCGGCACCCGCGC----------------------------------
CGCGGCTTCTTCCC-----GCTCCGCCCGTTCCCGCCC-
TCGCCCGTGCGCCCCGGTCCTGGCCTCGCGTCGGC---
GCCTCCCGGACCGCTGCCTCACCAGTCTTTCTCGGTCCCGTGCCCCGTGGGAACCCACCGGCGCCCCCGTGCGCC
CGGGGGTGGGCGCGTCCGCATCTGCTCTGGTCGAGGTTGGCGGTTGAGGGTGTGCGTGCGCCGAGGTGGTGGTCG
GTCCGCGCTGCGGCCGCGGGGTTGTCGGGGTGGCGGTCGACGAGGGCCGGTCGGTCGCCCTGCGGTGGTTGTCTG
TGTGTGTTTGGGTCTTGCGCTGGGGGAGGCGGGGTCGACCGCTCGCGGGGTTGGCGCGGTCGCCCGGCGCCGCGC
ACCC---TCCGGCTTGTGTGGAGGGAGAGCGAGGGCGAGAACGGAGAGAGGTGGTATCCCCGGTGGCGTT-
GCGAGGGAGGGTTTGG-
CGTC---

--------------------------------------CCGCGTCCGTCCGTCCCTCCCTCCCTCGG-
TGGGC---GCCTTCGCGCCGCA----CG----CGG-
CCGCTA---

GGGGCGGTCGGGGCCCGTGGCCCCCGTGGCTCTTCTTCGTCTCC--GCTTCTCCTTCACCC----GGGCG----
GTACCC-GCTCC-GGCGC-CGGC-CCGC-GG-----GACGCC--GCGGCGTTCGTGCGCCGAT---------
GCGAGTC----------------
ACCCCCGGGTGTTGCGAGTTCGGGGAGGGAGAGGGCCTCGCTGACCCGTT-----------
GCGTCCCGGCTTCCCTGGGGGGG-
GTCCCGGCGTCTGTGGGCTGTGCGTCCCGGGGGTTGCGTGTGAG-------------------------------
----

TAAGA--

TCCTCCACCCCCGCCGCCCTCCCCTCCCGCCGGCCTCTCGGGGACCCCCT---------GAG----
ACGGTTCGCCGGCTCGTCCTCCC--

GTGCCGCCGGGTGCCGTCTCTTTCCCGCCCGCCTCCTC------
GCTCTCTTCTTCCCGCGGC-------------------------------
TGGGC--

---------------------------------GCGTGTCCCCCCTTTCTGACC---GC-GACCTCAGATCAG

Hamster

TCAAT--CGCCTCCCCACCCCCCGCGG-
TGCGCCG--
--TTGGTGGGGGGGGT---
CGCG-CGGCT-GGGGGTTA--
TCTCGCAGGGCCCTCCCCTC---

TTGGGCCCTCCGTCTCCCTAAGTTCAGACGCGTGTTGGGTT-GCTTGCTGTTGGC--------------GGT--
GGTCGGTGGTCGTCGTCGTCGTCTCCCGT-GTGCGCCCCGACCGATGTCCGCACCCCCGCCGTTCCC-
TCGGCTTCGTCCCGAT--GCTCC----
GCTCCCGCCCGTCGCCCGCC---
---------TCCCGTG----
TGCTTCGAC--

--GGGCCGGTCGGT-
TCCCTGGTGGCAGCCGTGGGTTATCGGTGGTGTGGGGCGTGTCTCGGAAGGGGTCCGCGGGGCGCCCGCGGCCCT
CGTCGCCGCCGCAGCCCG----CCGGTGTGTGTGGACGAGAGAGAGGCAG---------------------
CGGA---------
CGGTGGGGAGGTGGGTCCCGTCTCGGGGCTGGGGGCGTCGTGTGTGTGTGTGGGGGGGTGTCGCGGAGGTTGGAT
GCGGTGTGGCCCGGTTCT--
AAGGGGAATCGGCCCCTATGCCCGCCGCTTCTTCCTCGGTGATCCCCCTTCCGGCTCCCGTCCCCACCCCGGGGC
GTCGTCTCTCCTTTCCCCCCTCCGG--GAGC---GTCGTCGCGCCGCA----CG----
CGCCC--

--------------------GCGAGGGTTGGCCGGGCCCGCGCCGCGGTCGCGCCTCGGTTCGTCGCG-----
AGCGTCGCCGGTCGTCCTCGCGTCTCTCTTCCTCCGTCTCCCTCGCCC----GGGG-----GTGCCC-GCTCC-
GGCGT-CGGC-CCGC-GG-----GACGCC--GCGGTGTCCGTTCGCCGAT---------
GCGAGC----------------GCTCCCTGGGGGTCGAGAGGCGTGTGGAAAGG-------
CGCGGGAATC--------------GGCCCCGGCGCCGCGCCCACCCCCGCGTACCC--
CGGTGTCCCGGCTTCGACGGGCCGCGCGCGCGAGCGTCGGTGTGGTGTGGGTGTGGGTCAGGTCAGGT-------
----

TCGGGG---
TTA--

CGGCCCCCTTCCTTTCCCTTCCCTCCCTCCCTCCAGACCCGCTCCGCGGCGACCCGTCGTCCCTCTAACAACGGG
TT-
TCTGCCGGCTCGTGTTCC---

CGGGACGCCGCCGCTGCCC--
---GTGGCTGGGAAAAAGGA------------

ATCTCTCGCCTCTCCCCTTGCAAGCAAGCAAGCAAGCAAT-----------------------------------
----

CCATTCCTATTTCTCTCTC-----GCTTCTC---GC-GACCTCAGATCAG

Cavia
TCAAT--CGCGACC---
CGCGGGTTGT---

CCCCCGGGGT---CGCG-CGGCT-GGGGGCTT--
CCTCGTAGGGCCCGCTTT---

GGCGTGCCCTCCGTCCCCCTAAGTGCAGACC------CGGTCGGCGC-
GCGCCTGCCTCGCCTCGCCGGCCGCCGCCCCCCCCGAGCTCAGCGCCCCCCCCCAGCCCCCGTCGCC--------
----

CGTC---

GGCGTGCGCGTTGGGGGGGTGGTGGTGGCCCGGAGGGGGGAGCCGCGCGTCGCGTGGTGCGGTGCCGCCGTCGGG
CG-----CAGAGAGGGAGCGGTGTTCGGAAGAGAGAGGGGCGAGCGAGA-----GGGC-
GTGCCCGAGGTGTCGGTGTCGGGGAGGGCGGCGGTCCGGGGTCCGGGGTTGCGGGGTCGCGGCTCGCGG------
----
TGAGC--
CCGCGGGCCCCCCCCCCCGCGCCGTCCCGTGT--CCGCCGCCC----
CCTGGCCGCGGTCCCTCCCGGTGGAGGAGGGGTCCCTCGCGCCGCA----CG----
CGGGTCCCGG---

AGAAATGTGTGCCCCCCCCCGCCGGGGTCGGCCTCGCGT-CGAGGCC------CGTGGC-----GCGTGT---
GAGGCCGCGCCCCCC-GGGGTCC--GCGTT--GCCC-CGGCGG-CGG-CCCGC-GG-----GACGCC--
TCGGCGTC-GTTCG-CCGTC--------GCGCG-CTGCGCCC--------------CGGGG-CCGCGCGT---
GGTCTCGCCGCGCTCCC-CCGTCAGTGGGTTGGGTCCCGAGGCCCCGCCTCCCCTCGGCGGGA-----
GGCGGACGGTGT-GGGGGGTCGAGGCGGCCGTGGTCGGTCGCCGGCCGGCCGGCTGCGCTGAGGGGTCGGGT-
GGCGTCTCGGTCGTCGCCGCCC---

GTGCCGC--
GCCGCGCCGCGCGTGCCGTGCCCGTCGTCTCCCCCCCAC------------------------------------
----
--------------------GTTGCCGCTCGGCGGGGTGGTGCGGTCGGTTCGAGCCCGGTGTCCCTCC-

GGCTCC-
GTGCCCTCCTGGCTCGCCCGGTCTCCGGCTCCACCGCGTCGCCCTTGTCTACCCCTCGTCTCCGTCCGCTTCCCT
CCCCGCCCCCCTCCGCCGCCACCGCTTCGG-
GGCGCGTGGCGACGTGTGGCGGTGGTGGTGGGGTGGGTCGCGTTCGGGACGGTGGGGGAAGGCGGGCCCGCGGTG
CCCGGGACCGCGCGGGCGGGC---------------
GTCATCGGGTGTGCCGTGGCCTCTCCCGCGCCGCCCCTCCACCTCGGGGTTCGCGCGGCGCACCGTGCTGCGCCG
CGCCGCGTCCTTCCCCC------
GCTCCGCCTCTCTCTCTCTCTCCTCCGGCCGCCGGCCTCCTCCTCTCCCCGCTG----
TGGGGTTTTCCGGCATTCACTCCCGGGTGAGACGACCGACGGCGAC-----------------------------
----

GCGAGACTCTCGCGTCGCGTCTCGGTCTCGTCTCCCCCT-CCC-TCCGAGAC---GC--------------

Diplo
TCAAT--CGCCCCC---CGGGGGT----
TGCGT--
---TGCCCCGCGGGGT---
GGCG-CGGCT-
GGGGGTGTCCCTTCGCAGGGCCCCCTTTCC---

GGGGCCCTCCGTCCCCCTAAGTTCAGACT-----
TTGGCGGTC--

---------------------------CCGTC-------
TGGCTTTTAGAGCGAGCGAGCGAGCGGTCATCCCGCGGGGTTGGTATCCGCGCAGGAACGAGCCCGCGGCCGGGT
CTTGTCTGTCCCGCCCCTCCCCCACCGTG--

TTC--
--CGCGGTGCCCGAACCGGGGGATGACGTTT-
TCGCGCCGGG----
CGCCC--

GAGTCGGGTCAGGGCGGCCCCGTGCCGCGGCCG--------CCGGCC----GCGAGCGAG---
CGCGTCGCGGGCTCCCGTGCCCCGC-GGACTC---GCAC---GCCG-AGGCGGACGGTCCCGC-GG-----
GACGCC--GCGG--TC-GT--G-CCGTCCGCCAGACGCGCGCGTCTTCCG--------------
CGGGGAGGGTTCCCC------

GCGCGCCGCCCGCGTTGCCGTTGCCGGCCCCGGGTCCCGCCCGCGTCCGTCCCCTCTC-----------------
----

-----------------------------------GCGCC-GGCTC---
GTGTCCCACCCCCGCCGTCT---

CCCGGACGG---TCGTCGGGCCGCGGGGCC---
TTCCCGTGCCGCGGGGGCGT---------GGCTCTCCCCCTTTGCCGCCGCC-G----
CCGCCGCCGCCGCCCTTCTGC--

--CTTCCTCCCTCCCTCCCTCCG---
TCCGAGAC---GC--------------

Sperm
TCAAT--CGCGCCCC--
CGGGGGTTTGGCTC---

TTCGGCCACGGCCCCCGCGGGGT---CGCG-CGGCT-GGGGGTTTT--
CTCGCAGGGCCCGC---

CTCGGGACCTACGTCCCCCTAAGCGCAGACC-----
CTGGCGGTCGTTTGGCGGTGTGCCGCTTCG---

TCTAT--

-------------------CGGATGTGGCCGCCGTCGCCGCCCGCCGGCCCGCCCGCCGCC-------------
ACCT-GCGAGTGGAGGCCAGAGAGGAGGAGAAAGGA-----------------TGCGCGCTTGCTTCCGG--
TCGGTCCTCGTCCTGCCCGGTGACGG---

GTCG---
---CCGTCGG-
CGCGCGGTCGGTGCCGCCCGC-----GGC----GAGGGCGTTCGGTGTG-TCGTG-
AGGGAGGGCGCTGGTGC----GCGCCGGTC--
GGCGTCCGGGTCGCCGCCCGCCCCCGGCGGCGGCCCGCCCGTGGCGGAGG---
CCTCCCG--

CCGCCGCCGCGCGGCACGGGCTGGCTCGGACATCCCCGGTCACCGGTCACAGTGCCCGCGCCCGCCCCGCCGGGG
TGGTGCACGCGCCTGCGGAGGAA----GGCGCGAGTGTGGCCGCGCCCC---GGGGGAT--GCGT---GCCC-
CGGCGG-CGAG-CCGC-GG-----GACGCA--GCGGTGTC-GTCCG-CCGTC--------
GCGCGTTTTCCCTCC-------------CGGGTCGCGGAC-----------
GCGCCGCGCCGCTTCCCCCCCGCCGTGTCCCTCCCGCGCCGGCTCCTCCGCACGG---
AGCCGGCGAGCGGCGGCGGGGGAAGGAGGTCGGAGCGAGCTCGC-------------------------------
----

GAGGGCGTCTCCACAGGTCTTGCCGCGCGCGCGGTTGGAGGTTTGCCGCCGCCGGCATGGCGTGTGTGTCGGTGT
GCTTGGCCCTCTTTCGGCGAGGGATTTGAGTCGGGAGCCAGCGGCCCGGCGGTCGAGGGGCGACCGTCGAGTTCC
CGCCGTGCCCCGCG---CCCCCCCCCGCCGTGTGAGCCTCCCGTCCGTCGCC-
GGCTCCCGCGTCGCCGTCCGCC---

CCGACGCCGTGCCGGCCGCCCTCGCCCTCGTTCCTCTTCTCCTCTTCCGCC-
CCCGGTGGGGCGCCTCCT-----------CCCGGAGGCCCTCC------------------
GTCGGCCCTCCTGTCGTCTCTGTGGCCTCGGGCGGGCCCCGTGTGTCTCGGGGGAAGGAACCCGAGCGCGGGAG
CCACG--------
GCTCTCCGC--

----GCCTCCTCCGCCCCTCTCTCGATTC---GC--------------

Ocho
TCAAT--CGCCCT----CGGGG--
TGGGGTTGGGCTC--
--TCGTCCTCCCCTCCCCGGGT---
CGCG-CGGCT-
GGGGGTGTGTGCTCGCAGGGTCCCCTTGCCCCTC---

GGGGAAGGGCCCTCCGTCCCCCTAAGTGCAGACC-------
GGCGGTGGGGCTCCGGCTGCCCGG---

CTC--

---TCCGGGCTCCGCGGTGCCGCC---------
GCCTGCCC-ACGAGAGAGGCGTCGTTGAGTGTGCCGAGGTGGGAAGAGAGAGGCGTGTGCGCGCG--
CGCGAGGAGGAAGGGGCTCGCCGGGCGTGTCGAGGAGAGGAAGGCAGGGAAGGGGACTCGTGGGGTCGGTTGGGG
GGAAGTGAGCCTCGCGGCCG---
GTGACGGCGCGCGGGGTCCCCCACCCCGGCCACGGTCTCGTGCCTTGCCGCCCTGTTCCCCGTGCGTCTTCCCGA

GTCCCGCCCCCGTCTCGCCGCGTCCCGTCCTTCTCTTTCCCCCCCGACCGCCCTTCGGGAGG---
TCGTTCGCGCCGCA----
CGCGTGTGGTCGGTCGTCGCGGGGGTCGAGGCTTGGGGGCGAGGGTGAGAGGCAGCGC-
GCGCCGGGAGCGGCGCCGGGT---
CTCC---

GACCCTCTCCGCTTCCCGGTTCGGCTCGCCCGATCCTCCTCCGCCCGAGACCCCCTCGCCG--
CGAGGCCGGTCTGACCTGGCGCGTGTGTCTTGCGCGTTCGCGCCC----GGGGAC---GCGT---GCCC-
CGGCGG-CGA-CCCGC-GG-----GACGCC--GCGGTGTC-GTCCG-CCGTC--------
GCCCGTCTCTGCCC--------------CGGGGGGTG-T-------------
GCGCGCTGG----------------CCCGCGCC-
GCGCCGGCTGGCCGCCCCGCCGCAGCCGCCGCCGCCACCCCACCGCC----------------------------
----

TGCC---

GGCGGAGGCGCTGGCTGGCTGGCGGGCGGGCGGGCGGGCGGGCGCGGCCCCGCGCGCTCCTGCTGCGGCCGTCCC
TCCCGGCCTCCGTC-----GCTCTCTCCGTGG----------------------------------
CGCTCGG--

-------------------------TTGTCTTCCTTTCCC--TCCCTCCCTCCGAGAC---
GC--------------

Orycht
TCAAT--CGCCCC----CGGG---
TGGGTGCCT--
---CGCGCCTCCCC--
GGGTT---GCG-CGGCT-
GGGGGTTTCTGCTCGCAGGGTCTGTA---

TTCTTTGCCCTCCGTCCCCCTAAGTGCAGAT--------
GGTGGCCCACTGGCCCCCGACTCCACCCTCCCTCTGCTCGTCTCGCGACTGTCCCGCGCGCCACCGGCCCCTTCC
CCCGTGCCGCCTCCCTCCTTCCCCGTCGCCCGCCCGCCCGCCCTCCCGTCCGGCAGGGGGCCTCCCCACGCGCC-
----
CTAGT--

GGCGCGGAGGTGGTTGTGCCCACCCCCGGCGGGAGATGGCGTCTGGGTGGCGGGGCGGGGAAGTGGAGGTGGGTG
TGGGGGGCGGGCAAGGCCGACGTGGGGGGCATGGTGCGAGGGGAGTGGGGGGCGTGTCTGCGGGCTGTGGTGCCG
GCTGCC----------------AGCGAGAGGTGGTGA-GCGC---GGC-----------GTGTGCGT----
TGGGGG--
GACCGAGGCCGCGGAATCGGTAAGGGAAGCGCCCCACGCCGTCT----------------------------
----
CGGC--
--------------------GTGGGTCCCCCGGCCGCGGTCCCGTCCTCCATCGGGAG-
AGCTCGTTCGTGCCGCG----CGC----GGTC-----
GCGGCGGGGGGAGAAGGGGGTGGTGGCGGCGCGGCGA------
GCGCCGGGAGCGGGGTGGCAGGTGGCTGTCTCCTCCCCCGCCGGTGTGCGC------------
CAG---

GTGCGCCTCGGCGGGGGTCAT---
GGGGAGGCCCCGGGCCTCTCCGCCCCCGCGCGTCTCGGCGCGCCGGCCCATGTATCCATCCGTCCTTCGCTCCC
GCGGCGCCGGGGTCGGC-TCGTG--CCGAGGCCGGGTC-------GGCGCGCGTGT-----GGTCGCGCCTC---
GGGGAT---GCGT---GCCC-CGGCGG-CGG-CCCGC-GG-----GACGCC--GCGGCGTC-
GTCCGTCCGTC--------GCCCGTCTACCCT---------------CGGGGGGTGCTCCC---
GCCGCTCCGCCGG--------------------------
CCCCGAGGAGACCCCGGCCTT---

---GGCCGTTGGCGT-
GGCTCGTGT-----
CTCCCGC---

TGCCCGCTCCTCTGCCTCTCCCTGCCTGCC--

-TTCCTTCCTTCGTCCC-TCCCTCCCTCCGAGAC---GC------------

Dasypus
TCAAT--CGCTCCCC--CGGGG------
TGTGCCGC--
--GCGAGCGCGCCTCCCCGGGGGTT--

GGCG-CGGCT-
GGGGGCTCTCCCTCGCAGGGCTTTTTTTTTTCCCCCCCCCCC---------------------------------
----
GGGGAAAAAGGCCCTCCGTCCCCCTAAGTGCGGAACC-----CGGGGACCG---
CGTCCGGGCCTCCCCGCGC--

GACGCGGGGCGGGTCCGGGCGCGGCGCCC-------------
GCTCGCGAGCGCCGTTGACAGAGCGCGTGTGAGAGAAGGGAGGG---------CGCGCGCCCGTGG---
CGGGAGT----
GCCGCGGACC---

------------------------------------CCCGCGGC-----
CTTTCCCCCTCGGGGGAGCAGCGCCCTCGCGCCGCA----CG----CGGCCT----------GGG-----
GCCGCCCT-----GGCGCGCGTGCGCGCCCCGGCGCGGGGCCGGTGCCGCGCGGCG-
CTGCCGGCGCGCGCGGGGGGTTGGGGGGCCGCTGCCCGGCTGCCGTGAGCCGTCCCC------------------
----

GCGGT--

GGGGCGGCCCGGCGTCCGGCCGGCCCTTCCCTCCCCCGGCGCCGCCGCCGCGCGCGCGCGCGCCCGCGTCCCCGC
CC--GCCGCGGGCGCCGC-CGCGCCTTGTCGCGT-GCTGCCGGTCCTC----GGGC--------
CCGAGGCCC-------------GCGGC-GCGGGGTTCTGCGCGCCCC---AGGG-----ACGCGT-GCCTC-
GGCGG-CGAC-CCGC-GG-----GACGCC--GCGGGGTC-GTCCGTCGCT---------
GCGCGCTCT--------------CCCCCTGGGCCGCC-----------GTCGCCGCG-
CCGCGCCGCGCTCTCGGG-----------CCCGAGGGGGGCCGGAGGCGGCGTGGGGGGG-
TGGTGTGGGAGCCGGGGAAAGCCGGCCGCGGGGTCCGCAGGGGGGGCGGAGGGGGGGCGGCGGCGTGCCGC----
----

GGGGCGCTCAG--

GCGCTCCGGCCGCCGCCGCCGCCGCCGCCGCCGCCGCCCTCGGCCCCGCCGCCGACCCCGCCTCCCCCCCCCCAT
CGCAGGGCCCC--GCTCCGTG----CCGCCGGCTCGTGGCGTTCCCGTCCGGGCG--
GTCGCTCCGGTGACCGCGGG---

GCGTC--
----------------------TCCGCGCTTCCCGGCGCCCGCCGCC----ACCCC--TCC----
CCGCGACGGCGCGCG-CGCCCCGAC-------GCTCCCCTCTCTCGCCGCCTCCCGTCGC---------------
TCG----
CGGGCTCCC--

--------------------GGTCCCCGCCGGCCGGCCCGGCCCCCTCCGAGAC---GC-GACCTCAGATCAG

Choleops
TCAAT--CGCCTTCCG-
TGGGGGGGGGTTGTG--

AACGCTCCCCCGGAT---GCG-CGGCT-
GGGGGTTTGCCCTCGCAGGGCCCCCCCCGCTTT--

GGCGGTGGGGCCCTCCGTCCCCCTAAGTGCAGACC------CGCTG-
CCGCCCCGTCCGAGACTCCCCCGCCG---

CCGCGTC--

-----GTGCGGGGGCCGGGGTGTGGCGCC--------------
GCTCGCGAGAGCCGAGA-----------------------------GAGA------------GTG---
CCGGGG--
GCTGGTCGTGGACC---

--GCCGCGGCCCC----------TTCGG--GAGC---
GCCCTCGTGCCGCA----CG----CGGTCT----------GGG-----GTCGCCCT-----GGCAC-
GCCGGCGCTGCGCGGTCCCCCGTGTCCCGCGTCGGTTCCCGTGGTGGGGAGCAAGG---
GTCCGGGTCGGCCGTGAGCCGTCACCCG---

CTC--

CGGGTGGCGGCCCGGTGTTCCCCGTCCGGGCCCCCCACCCCCACCCCGGCCCGCCGCGTGTGTCCGGGGCCCGCC
CGTGCTCGCCGC-CGCGCCTTGTCGCGT---TGTGGAGGCTTC---GGGAC-------
CCGAGGCCGA--------GCGCGTGC----CGGAGA---TGCGCCCC---AGGG-----ACGCGT-GCCCC-
GGCGG-CGAC-CCGC-GG-----GACGCC--GCGGTGTC-GTCTGCCGCT---------
GCGCGCTTT---------------CCCCTGTGCCGCC-----------GTCCCCGCG-CCGCGCCGGCC-----
CGTGT------GGCCTGAGGT---GACGT---
TTGGGGCCGTGGGGACGGCGTCGTTCGCGGCGCGGGCGAGAGCGGAGGTCCGGTGGGCGGCCGCC--------
----

CCGGGT--
CCCG---

ACCCTGGCGCGTCGCGGCGGCCCTCCGTGCACGAACGCGTGCCGGACGGTGTCCCCGGCCCCGGC--
CCCAGCGCAGGG--TCCCGCTC-GTG----CCGCCGGCTCGTGGCTCCC-------------
GCCCTCCCGCCCTCACCCCGCGCCGCCGTTCGCAACCCC------------------------------------

----
GCC---
GGGGGATCGGTGGAGCGCGGGGTTGGGGGGGGTGGTCCC--
CCACACGGGCATGGGCCGCGGCGGTCTCGGCCACCCAGCGCCCGCCTCCC-----GCGCGGTCGGTCGG---CG-
TGTCTCCC--GCTCTCCGTCCGCGTCTCTCTTCTCGCCTCCTC----------TCG----CGGGC-
CCTTCGGTCCGCGTGGC-
GGGCCGCTCGACGGCGGCGTCGGCGGGTTTCGCGGGGAAGGAGG-------------------------------
----

CTG--
-----------------------GCCCTCTCTCCCCCGCGGCCTCGCCCCGCCGCCTCCGGCGCCC-
TCACGCGTCTCCCCTCCCGAGAC---GC-GACCTCAGATCAG

Echinops
TCGAT--CGCTCCC--TTGGGG-----
TTGCCC---
--GCCCTGGGGGTT---
GCG-CGGCT-
GGGGGCCTTGACTCGAAGGGCTCT---

GCCCTCCGTCCCCCTAAGGACAGACCAG----
CGGTGCCCCGCGGGCGGTCGCTTCTCGCTGCCGGCCCTCCCCCGTGCCTCGGCTCTCTTTCCCCGTGCCCCTTGC
CGCTGGC--

TTT--

GGCTTGTGGCGGGCGTCCCGGGGGTTGGGTTGCTTGTGGCTTTGGGGGGCTGGGCTGGGCGGCGGGGGGGTTTGG
CCTTGGCGGTTTGGCACCG------------
CGCCCGCGAGAAAAAAAGAGAGCGGAGCG-----------------CGAGAAGCA----GCTCGCG---
TTGAGGTTC--

---GCGG--------------CCT--
GGGAGACC-GTCCTCGTGCCGCA----CG----CGGATCC-------TCGGG-----GTCGCCCT-----
GGCAC-CGTGT----AGGGGGGACCG-
GGCGCTCACGCTGTGGTGTGGTGGTGGTTGCGGAGGGGAGAAGGGCGTGTCTTCC-------------
CGGAAAGGTGGTGCTGAGGAGGAGAGGTGGGGGGCGTGCCCCCGCGTCCGCGGCCCTCGTCGCCCCGACGCCA--
----
GCCCCC---
-TGGCGGGCGGTTCGCGGCGAGTTGCCCGGCTCGGTCTCCCTGCCCTCCTCGAAGTCCCTTTCCGGTCCGCC-
GTCTTGCTCCTCTCTCCTCGCCCCCCTCCGCTCGCGCGCCCGCCGCCGCCTCCTCGTGCGCGCCTTGTCGGGGCC
----GCT---------------------CCGGGCCTCTGCCGC---GCGCGCGT-------CGGG-
CTGTGCCCC---GGGTT----ACGGGT-GCCCC-GGCGGGCGGC-CCGC-GG-----GACGCC--GCGGTGCC-
GCCTTCCGAT---------GCGCGCCTCT--------------CCCTCGGTCGC-------------
GTCCCCGCG-TCGT----------------------GCCGGCGGGGCTCGT---------------

CCCGGCCGGGGGTGCTCGGCCGCTCGCCCGGTTGCCGCGGCCGGGCTCGGACGGTGTCGGCGGGAGGAGCGGGTG
TCGGTGCGTGGCGGGGGCGGGTTCGTT--

GTCGCGGCCG--
GGG--

GTGGCCGCGACCCGTTCCCGTGGCTCCGTCCGCCCCCGCCTCAACCGTCCCCCGCACGTCCGGGCCCGCGTCGCC
GGTAACCGCCGGGGCGGCC-GCCGGCTCTCCC-TGCAGGGCCTCC-GCTC--GG----
CCGCCGGCTCGTGGTCCCC-------------
GTCGCCGCCCTTCCACCGTCCGTCCTCCCGCCCCG--

CTCGT--
-------CGTGGGCGGCGTGGGCGCCGGGGTTGGGCGGTG---
GCTGGGCCGCGGCCTCTCGGGGTACGCGTGT------------------------
GCTCTCCGCGCCCGCTCTC------------------------TCGC---TGGGCTC-
CGTCGCGTTGGCGATCGGGCGTGTCGGGTGTTTTGGGGTCGCGCGCGTGGAGTTTCTCCGGCCGTGCGTGTAGTT
TGGGTGGCGGTTGGGGGTCG---

TTCC---

CGGCCCCCTCCCTCCCTCCCGGCTACCTCCGCCGCCGCTCCCCGCGACCCGTCCCCGTCGCCCGCCTCGCCCCTC
GCTCCCTCCGAGAC---GC-GACCTCAGATCAG

Loxo
TCAAT--CGCCCCCCC-CGGGGT----
TTGCCGCCGGCCAGCGT--
--CTGGCCGGCCCTCCCTCCCCGGGGGT----
GCG-CGGCT-GGGGGCTT--
CCTCGCAGGGGCTGGCCGGCCGGCCCGCCGCACGCCCGCGCCCAAG-----------------------------
----
CCTGGGCGGCGGGAGGCGCGCGGGCGGCGGCCGGGTTCCCTACGACCCCCTAAGGACAGACC------
CGGTGGTGCCCCCGTCCGCCCCCCTTCCCTCCGCGGCG-------------------------------------
----

GCGGAGGAGGAGGAGGGGGGCCGGGGGCCGCCG------------
CGCCCGCGAGAGAGAAGAGAGAGAAGCGAGG---------------CGAGA--------GCTCGCG---
CCGGGGTC---

---GTGG--------------
CTCGGGGGCCC---TCCCTCGCGCCGCA----CG----CGGCCT---------CGGG-----GTCACCCT-----
GGCGACGGGGGGGGGTGTGTG-CTCGCC-GGGGG-
AAGGCGGTCCCGCGCCGCGCGGCGGGGGCCGGGGAGGGGCGA------------------
CGGGGAGGGGAAGCGGCGTCCGCGTCCGGGGCGCCCCCGTCGGGCCGCCGCC-----------------------

----
TGCCCCC---
-------GGCGGTGGCCG-
CGGGGGACGTCCCGGCCGGCCTCCGTCTCCTCCGAGCCGCTCTTCCCCACGGTCC--
GCCGCGTGTCCGGGGCCCGCCGGCC---TCCCGGCGGCCCCTTCCAACCCCCC----
GTGCCTTGTCGGGAC-----GCC-TC-CG---------------CCGGGC--------------
GCGTGCGGGGGG-CGGGGCTCCGCCCC---AGGCC----ACGGGT-GCCCC-GGCGGGCGGC-CCGC-GG-----
GACGCC--GCGGTGTC-GCCCGCCGAT---------GCGCGCCTCC--------------
CCCCTGGGTCGTT-------CCCCCCCTCCATG-CCGCG----------------------CCGGCGAGGCCC-
GGCCCGG---------------
CGGGGGCGCCGCCGGGGCGCC---

ACCG--

GGCGCGCCGGCGCCCCCT-GCAGGACCCC--GC---GGG----CCGCCGGCTCGTGCCCC-TCCCT-----
CCCCGCCCCGCTTCCCGTCCCCGTCCGCCCGCCCCGCCCGGCCC--------------------------------
----
GCGT---
GGCGCCGGGGCGGGGCGGCGGGCGGGCGGGCCGGGCGTGCGTGGGCGGCGCGTGCTGC-
CCGCGGCCCTCCGGC--ACGCGTCCCTCTCCGTGTCGCGC---------
GCTCTCTTCTCTTCC------------------------------GC--GCGGGCCCCTGGCGTT-
GACGATCGC--GTGGCCGGCCTCCCGTGAGCGCGGGCGCTCGC-
GCGGGCGGCGGCGGCGTTGGCGAGCGAGAAGACCCTCGTCGGGCTCCCGGTAGGGGGGTGGGCGCGGGCGCGCGG
CGGCGCCCGGCCGCGCCCGGTGTGTCGTCGGCCGCGGGTGCGGGGGCGCCCCGCGGCCCCTCCCCGAGC------
--------GCGCCGCC-----
GGCGCCGTGTGCGCCGCCTTTCCCCCCCCTCCGGTCCTCCGCCCGTCGCCGTTCCGCCTCGCGCGGCGCCGCCCG
GGAGCCGGGCCCCCACGCCTTCCCGGCTCTCCGC-TCCCTCCGAGAC---GC-GACCTCAGATCAG

Procavis
TCAAT--CGCCGCCCT-CGGGG------TGGC-
CCGGCCG---
--AGGGCTGGCCTCCCGCCGGGGGTGT--GCG-
CGGCT-GGGGGTTC--
CCTCGCAGGGCCCGCCCGGTCTC---

GCGGCCGAGGCGGAGCCCTCCGTCCCCCTAAGGGCAGATC------
CGGCGCCTCCGTCGGCCCCACCTCGCGCGTCGCCTCGCTCACCGCCCCCGCCGCCCCGCCCCGCGGCGCGTCGC
CCCCC---

CCGGCGGCGCGGTGTGCGGCGGCGGTGGCGGCGGGGCGGAGGCGTCGGTGCGCGGGTGGGGCGGGCGTGGGGCCG
TCG-----------TGCCCCGCGCGGTCCGGAGGAGCGCGGCG-----------------CGGAGA--------

GCTCGCG---
CTGGGGCCGCTGCC---

GCGGCCGAGGGGTGTAGGGCTTAGTTAGCCCGCCTCTCGCGCCGCAA---CG----CGGCCC---------
CGGG-----GTCGCCCT-----GGCGTTGTGTCCGGGAGGGGGCGGGC-GGCGCGTGGTCGGGG-
GTCTGTGCC----
GCGGGGGGGGGTGCGTCCGGGCGTTCACGGGTCGCGCGGCGGGGACCGGGAGGGGCCGGGAGGGGCGGAGGCGAG
GCNNNNNNNNNNNN---

NNNNNNNNNNGTCGGTCGCTCCACCGCCGCGCGTCCGGGGACCG-----CCGGCCTCCCCCCGGGGCCTC-
TCTCCCGGCCCGCTTTCCCCCGCCTTCCTCCCCGCCTTGTCGGGGA----CGGC-TC-CGCCG------------
CCGAGA--------------GCGTGACGG-GAGGGGTGCCGCGCCCC---AGGTCT---ACGGGT-GCCCC-
GGCGGGCGGA-CCGC-GG-----GACGCC--GCGGTGTC-GCCGGCCGAC---------
GCGCGCCTGGCTCCGACCAACCCCCCCGTGGGT--------------
CGCGGCCCAACCCCCCCCGCGCCGCCACGCG-------CCGGCGAGGCTC-GACCCGG---------------
CGGGGGCGTCG--

CCG--

CGGCGCCCCC-CGCAGGACCCC-------GGGAA--CCGCCGGCTCGTGGCCCCTCCCCCT----
GCCGCTCGCCGCGACCCCGCCCCTGTCCCCGGCCGCCCCGACCCGGCCGCCC-
TTCCCCGCCCGCCCGCCCGCCGC---
CCCGT--------------------
GCGGCGGGCGGGAGGGCGCGTGGGGGGGCGGGCAAGAGGGGTCGGGGAGGGCCCCGGGGCCGGTCGCGGGGCGGC
GGCGGCGGCGGCGGCGCCGCCGGCCGCGGCC--CCGGCGCG-TCCCCTCTCGGCGTCCGC-------------
GCTCTCCTCTTCCCTCGGCCC----------------------TCG----CGGGGCT-CTGGC-CGTC-----
CGGGCGTTGGCGATCGCGTGGCTCGGGCCCTCTCCC---------------------------------------
----

GTCCCGTCGACGGGGCCCGCCGCCCCCCCAAACGCCTTCCCCGCTC-TTCCCCCTCCGAGAC---GC-
GACCTCAGATCAG

Pteropus
TCAAT--
CGCACCCAC--

----------GGGT----GCG-CGGTT-GGGGGTTG--
TCTCGCAGGGGCCCGCGCCGTCGCCGG--

CCGGGGCCCTCCGTCCCCCTAAGTGCAGATC------
CGGCGGTTCCCGCCGCGGCGC--

CCC---

CGTC---GCGCCGCGGGCGGGGGCCGCGCC-------------GCCCGCGAGAG-
CGGGAGAGGCCGAGAGACG------------CCGAGG------------------
GCGAGGTCCCG---

---GGCGCCACGGTC-----
CCCGCGGGGGCC---CCCCCTCGAGCCGCA----CG----CGGCCC-------TGGGGGG---
GTCGCC--------
CGGGCCTG--

GACCCG--

GACCCGCGCTCGTCCCCGCCGGGGC-----GGC-TCGCGCC-GAGG--------
CCGAGTC--------------------GCGCGCGGGGCCGCGCCCC---GGGG-----ATGCGT-GCCCC-
GTCGG-CGGC-CCGC-GG-----GACGCC--GCGGCGCC-GTCCGCCGCC---------
GCGCGCTTCC---------------CCCCGGGTT-------------GCGGCCGCG-CCGCGTCGCGCGCC-
CCGAGCC------CGCGGAGGGGCC-GGTGTCGCGTGCGCGC----GTGGGGCGTGGCGCCGCCC---
GTCGCCCGGCGGCGGCGGCGGC-GGTGCCCGTCGCGGCGGGCGCG---GGTGGTTGT-
AGGGTACGCGGCGTGCGGGACCCGGGAGAGGGCGGGCGAG----------------------------------
---------------------------------------CGAGCGGGCGAGCG--AGCGAG-----
CGAGCGCGAGGCNNN---
CCGCCCGTCCCCGCCTCGCCTCCCTCCCTCCCCGCGCCGCCGTCCCCGCTCTCATCGCGCGGCCCCGTGGCGGG
CTCCCCGCCGCCTCCGGTGGCGTGGCGGCGTTCGTCGCTCCCGCCCTCGCCCCCGCCGCCACCTCTCCTCCCCGC
GCGGGGCGGGCCGGGACCCCTCCCCCCAGCCCCGGTG----CCGCCGGCTCGTGCCCCC-------------
GTCCGCCCGCCCGCCTTCCCGTCCGC--

TCC---
GCGGTCGGGTGGCGACGGGCGTCGACCGTGGCCCGCTTCCCCCCGCGCCGCCGGCACGGCCGGCGCGGGCGGTG
GGGGCCTCCCGCGGCCGCGCCCCCCCCGC------------------
GTCTCTCCTCTCCCCG-------------
CCACCGCCGCCGCCGCGTCTC---

CCGCGGCGGCCCCTCCGTCC---GC-GACCTCAGATCAG

Myotis

TCAAT--CGCGTCCCC-CGGGG------
TGCC---

GTGCGCCCCGGGGTGTGCGCG-CGGCT-GGGGGTTT--
CCTCGCAGGGCCCGTT---

CCCGGGCCCTCCGTCCCCTAAGTGTAGACGA-----
CGGTGT---

------------------------CTGTGCC--------------
CCCCGCGAGAGACGGGAGAGGC--------------------
CCCGAGGCC--

-------ACGGTT-TTCCCTCTC--GGGAGC---TTCCTCGCGCCGCA----CG----
CGGCCAACGGGAAAAAGGGTTGGGGTCG----------
CGGGGCCCGTC--

GTTG---

---------------------------------GCGACGGGGTCCCCTTCCCCGCCCCGGCCCTGCCGGGG-
CTGT-GGC-TCGCGCC-GAGG--------CCGAGTC---------------------
GTGCGCGGGGTCGCGCCCC---GGGG-----ATGCGT-GCCCC-GGCGG-TGGC-CCGC-GG-----GACGCC--
GCGGTGTCC-TCCGCCGCC---------GCGCGCTCCT-------------
ACCCCCGGGCA--------------GTGGACGCG-CCGCGTCGCGCGCTGCCGTGCC------CGC-GGG----
CGGGTGTGTGTGTGTGTGTGTGTGTGTGTGTGTGTGTGGTGGGTTCGTGTGCCGTGTGGGGCGGCGGCGGCGGCG--
----

GCG--

-------------TCGTCGTCGTCGTGGCCCCCGCGTCCACCCCCCCACCCGCAGACCACCACCAAACTCAC-
CCCC-----GCTCCCCCCGGCAAAAGGCCGCCCGTTCTCCGCCGGCTCGTGCTCCT--------------
GTCCGTCCGCCCGCCTCCTCGCCTCGCCCCG--

TTCCTC---

CGGGTAAGGCAAGGCAGAGAGAGAGGCGGGACGTGTCGGCCGTGGTGTCCCGCGTGCGTGCGGGTAAGG------
--------------GTTCCTCTCCC----------------------------GGCCTCGC---
CGGGGG------AAAGCCTC-TCTCTCC---

ATGGCGCG---

CGTGTGCGCGCGCCCCTTCCGAGAC---GC-GACCTCAGATCAG

Canis
TCAAT--CGCCGCCCC-
TGG---

TGGC---GGCG-CGGCT-GGGGGTTC--
CCTCGCAGGGCCCGGTC---

TGCCGGGCCCTCCGTCCCCCTAAGAGCAGACG------
CGGTGGCCGCCTCCGCGTGCCTCCCTCCTTCCCTCCCTCCCTCCCTCCCCCGCTCCCCTCCCGTGCCCTTCCGCC
GCGCCCGGTCCCGGCCGCCCGCGCCCCCCGCCCCC--GCACC----------------
CGTGC---

GGTGTGGTGCGGTGCGTGGGGGCCGGGGACCGGGCCGGCGGCGGGGCCGTG---CGTGGGGCG------------
GGGGTGGGGCGGGCGGTGGGGCGGGGGTGTGGGGGTGACGTGAGAGGC-GGTGCCC-------------
GCCCGCGAGAAAAGGAGAGAGG-----------------------CGAGA--------GCTCGCG---
CCGAGGAGGGC-
CGCGGCGGCTGCCGC---

------------------------------------CGCCGCCG-CGGTC---CCCTCTTGGG-GAGAT---
CCCTCGCGCCGCA----CG----CGGCCAC---------GGG-----GTCGCC--------CGGGTCGTGC----
GTCTGGGGCGGAGGAGCGGGGGTGTGTGGTT---

GCG---

GTCC--

CGCGCCGCCCCTCCGCTCCCCTCCCCTCGCCCTTCCCCGCTGGCG-CCCCC--GGCC-----
TTCGCCCTGTCGGGGC-------C-
TCGCGCC---GGGCCGCGCCCC---
GGGGC-----TGCGT-GCCCC-GGCGG-CGGC-CCGC-GG-----GACGCC--GCGGCGCC-
GCCCGCCGCT---------GCGCGCTTCCG---------CCCCCCCCCGGGT-------------
CGCGGCCGCG-CCGCGCCGTTGCTCC-------------CCCGGAGCCCGCGGTTGGGTCGCGTCGGGGT----
CGAGGCG-----GGC---------GGAGGGTCGCGGCCGT--
GGGAGGGACGTCGCGCGCGAGGAAGAGGGAGGCGTGGGGGCCGGGTCGTCGCGCGGGGCGGC-------------
----
GGGCGAGGGCGTCGC----GGGCCGGCGGTCG-GGGG-
CGACCGCCGCGACCCGA---

CGACGTCCCGCCGCCCGCCCGCCCGCCGGCCGGCGCCCGCGCCCCCCCTCTCCCCTCCCACGCGGCGCCCGCGCC

CCGGCTGCCCCCTCCGTCTGCGCGGCTCCCCGCCTCC-GGTGGCC--GGGCAGGGCCCCTTGC--CGCCG---
CCGCCGGCTCGTGCTCCCTC------------
GCCC--

----------------------------------GGGGTCCCCCCCGGGGGGCCCC-----------
GTCGCCTCTCGTCCTTGGCTCTCTCTCC--CGGCC--------TCG----CGGCG--
ACTCCCGCGC--

------------------GCGCGCGCGCGCGCGCGCCCTCCGAGAC---GC-GACCTCAGATCAG

Felis
TCAAT--CGCCTCCCC-CGGG-------
TGGGTG--
---TCTCTCCCGGGGGTA-
AGGCG-TGGCT-GGGGGTTC--
CCTCGCAGGGCCCGCCCCGCCGGCGTCCGCCCCGCCCGAGGGCGCCCCGCCACGCCCGCCTGCGCGGCGGTACGG
TGTGCGGGGTCGTCTGCGGGCCGGGGAGAGGGTCGTCCTGGCCGCTGGGCCCTCCGTCCCCCTAAGTGCAGACGA

CGGTGGCCGCCGCTGCCGCCCTCCCTCCTTCCTCCTCCTCCCCCTCCCCGCCCCCTCCCCGGCCCCGTGTG-
---
TCCGGTCCCCGCCGCCTCCCTCAGCCCCCCCCCCCCGCCCCCGCCTCGCCCCGCCGGGTCCGCT-----------
----

CGGCGGTGGCGTGTGTGTGGGACGGGGGGAGGGTGTGGGACGGGGGGGGGCTGGGGGCCGGCGCCCGTGGGGCGG
TGGTGTGGGGGGGTGGTTGGGGGGGGAAAGGTGTTGGGGGGGGAGAGGCTGAGGCAGGCCGGTCGCC--------
-----GCCCGCGAGAAAAGGGAAGGGC--------------------GAGGAGA--------GCTCGCG--
CCTGAGG---GC-
CGCGGCC---

-----------------------------------GCCGCCG-CGGTC----CCTCT-GGGGGAGAT----
CCTCGTGCCGCA----CG----CGGTCTT--------TGGGG----ATCGCCACC-----CGGGTCT----
CGGGA-GGGCTTTGCGGTGCCCC--GTGCCGCGCGGTCCCGTGGGCCGTC---
GTGGGGGGTGGAGGTTGTGGATCCGGAGCGCCGTTGGTCTC-------
GCCGCCT---

TGCCCC---
GGGCGGCGGCCGCGG------------
CGGTCTGGCCGTGGCCCCCTCCTCTCCCGGTCCTGTGGCCGTGCGTCCGGGGCCCGA---------
GCCGCGGCCCCGCCCCCGCTCCCCGCTGGCGA-TCCC--GGCCCCCCTTTTGCCCTGTCGGGGC-----GGC-
TCGCGCC-GAGG--------CCGAGTC-------------------GCGC--GCGGGGCTGCGCCCC---
GGGG-----ATGCGT-GCCCC-GGTGG-CGGC-CCGC-GG-----GACGCC--GCGGCGTC-
GCCCGCCGCT---------GCGCGCTTCCC---------------CCCGGGT--------------

TGCGGCCGCG-CCGCACTG--------CGTG--------CCCCGAGCCCGC-GTTG--
TCGCGGGGGGGTGTGGTTCTCCGCGGGGTTTTCGTTGGTTGGAGGCGTGCCGCGCCGTCCGCCGGGCCCGGCC
CGGGGCCGCGGCGGGAG-----
CGCGGGGCACGTGGACGTGTGGGGGGGAAGGCCGGGTGTGTCGC--------------------------------
------------------------GTGGGGGGAAA---------AGGGCCGGCGGTCG-GGGG-
CGACCGCCG---
CGCTTGGACCCCCCCACGCCCGCGCCCGCTTCTCCCCTCGCTCCCCCGCCCCGCGCCGCTCGCCGCGGTTCCCC
CTCCGGGGGTCGTCGCGAGGCGGCGCGTCCCGCGCCCCGGCCCTGCCCAAACCCCGTTCCTCCGCCCCCTCCGC
-CTTCCAACCCCGGCAGGGCTCCC-GCC-TGTG----CCGCCGGCCCGTGCTCCCGCCT----------
GCCCGCCCGCTGTTCCGCTGGCCGTCCCGCGTGCGCACCCTTACCTTCCCCGTCCCCCCTCCCCCTCCCGGCTCT
CTCCCCTCCCGCGCGCTCTCGCTTCCCTCTCCCTCCCTCCCTCGCGGAGGGTTTGGAGGAGGGGGGGTTGTTGGT
GCGTTGCGGGGAGGGGGGGGTCGTCGGTTGTGGGGGGGTGGCGGCTCGGGCGTTGTGTTTGGGGTTTTGCGTCGT
TGGGTGTCGGCCGGAGAACCGGGCGGGTTGCGGAGCGCGGCCTCTTCCCGGGGCCGGC------------
GTTCCGGTCCTCTCGTCGCCCCTCCGCCCTG---------GCCTCG----CGGG-G--
CTCCTCCTGGCGCGCG--

---------------------------CGCGCGT-CCCTCCGAGAC---GC-GACCTCAGATCAG

Equus
TCAAT--CGCCCC----CGGGG------
TGGGTGTCG---
--CCTGCCCCGGGGT----
GCG-CGGCT-GGGGGTC---
TCTCGCAGGGCCCGCC--

CCGGGCCCTCCGTCCCCCTAAGTGCAGACG------CGGTGCCCCTCCTCC----
GCCCCGCGCGCCCGCCCCTCCTCCCACGCGCCCC--

GGCGCCCGGTCGTCGGAGGCGCGTGGGGGTCGGTGGCGGCGGCGCCGCGGGGTCCCGGGGTGGGGGCCGCC----
---------GCCCGCGAGTCGAGGGAGAGGCAG---ACG---------------CGAGA--------
GCTCGCG---
CCGAGGTGCCCGTGGCCGCC--

---ACGGTG-----
CCTTCGGG--GGC---TCCCTCGCGCCGCA----CG----CGG-CCT--------CGGGGT--------------
GGCCGGGGC------GGGGACGAGACGGGTCCCGCGGCGCGTGGTCGGGGCCGCCGTGT--------
TCGGGGGGCCCGCGTCCGGGCCGCCTG--TCCGGTC------------
GCCGC---

TCGCCCC---

GTCGGCGGTCCGTCCCGGTGCGTCCGGCCGGCCCCTCGCCGGTCCCCGGCGCGCCC-GGGTCCCGGACC---
GTGACCCCTCCCCGGCC---------TCGCC------------CCGTCGGGG----T-GGC-TCGCGCC-
GAGG--------CCGAGTC-------------------GCGTGCGCGGGGCCGCGCCCC---GGGG-----
ACGCGT-GCCCC-GGCGG-TGAC-CCGC-GG-----GACGCC--GCGGCGTC-GTCCGCCACC---------
GCGCGCCCTCC---------------CCCGGGT--------------CGCGGCCGCG-CCGCGCTG--------
CGTG--------CCCCGAGCC-CC--
GGGCGGGCGGGCGGGCGCCGCGTCCGCCGCCCGACGGGCCGTGGCGGCTCGCGGCGGAGGGGCGGGTGTCGGGAG
GCGGTGGGACGCGCGCGGGAGGTC---

--GGCGGTCG-GGGG-
CGAGCGCCGG---

CCCCGCCGCGCCCGCCGCCGCCCCTCCGCGCTCTCCTCCCCCGCGTCGCCGCGACGCGTCCGCCGTGCGCGTGGC
GGCGTCCCGCTCCGCCCCCCTCCT--------CCCCGGCGGGGCCCCTCGC-CCGTG----
CCGCCGGCTCGTGCCCCCGTCC----------
GCCCGCCCGCGTCCCTCCGCCCGCCCGCCCGCCCGCCCTC-----------------------------------
----
CTTC--
GGGGGTCGGTCGTGGCGGGGGGCGGGGCGGAAGGCCGGGCGGACG--TCGGCCGTGGCCTC--GCGCGCC---
CGGGGTCC--TCCTCCGCGGC-------GTTCGTCTCTCCGTCGCTCCC-CCGCC------------
TCGCTCG----CGGGCTTCCC---------
GCGCGCGGCGG--

----CCGCCGCCGCCGCCGCCGCGCCCTCCGAGAC---GC-GACCTCAGATCAG

Sus
TCAAT--CGCCACCCC-CGGGG------
TGT--
---TTCCCTCTCC-GGGGGT---
GGCG-CGGCT-GGGG--TGT-
CCTCGCAGGGCCGCCGT--

GTGCCCTTCGTCCCCCTAAGTGCAGACAC-----TGGCGCGCCCCCTCT--
GCCTCCTGCCGTATCTGCCCCGCACCCCACCCCCCCCCCCACGCCCTGCCCCCGCCGCCCGCTCGC---------
----

TTGACGGAGCGCGTGTGGGACGGTGGGGTGGGTTGGCTGGGGGCGGGCGGCGGCGGCTGCGAGGCCTTGGGGGGG
CGCGCGCC-------------GCCCGTGAGAGGGAAGAGACGGACC--------------------
AGAGG--------G-TCGCG---CCGAGGCCGT--
TGGCCTTGG--

-----------------------------------CCGCCGCCGTC--------CCTTT-GGG--AGC---
CTCCTCGCGCCGCA----AG----CGGTCTTT-------TGGGCAT-GGTGTGTTTC---GCCGGGGG-----
TGGGGTGGGGGCGTCGGGCCGAGTCCCC-GCGCCG-CGCGGCGGGAGCCCGCG-
CGATATCGGTCGGGGGTCGAGTCCGGGCCGCCGCCGCCTCCCGTTTGCCGCG-----------------------
----

CGCCCCCAA---
CGCGGCGGCGGT----------------------GGCGGCGGT-----------------
GTCCGGCCGGCTCCCTGCCGCGGTCCGCCGCGCCGCCTGGGGTTCC-
TTCCGCGCCCCGCGCCCCCCCCAAACAA-CG-CCTC---GCCCTGTCGGGGCGGGC-GGC-TCGCCCC-
GAGG--------CCGTGTC------------------GCGCGCGTGG---CCGCGCCCC---GGGG-----
ATGCGT-GCCCC-GGTGG-TGGC-CCGC-GG-----GACGCC--GCGGTGTCCTTCCGCCGCC---------
GCGCGCTTCCC---------------CCCGGGC--------------TGTGGCCGCG-------CGGTGCCT--
CGTG-------CCCCCGAGCCC--------
GCGGGGTTTTGGTGTCGCTCCAGGGGGTTGGGCGCCCGGCTTCCAGCCACCCGTCGGTCGTGGGGCGTCTTGGCC
GCACACGCGCGTGAGGTTGCGT---

GGAGGGAGAGAGGGGGAGGCAGGGGGGGCCCCTGGCGGTCG-
GGGGGCGACCGCCGGGTTTTT--

CTTCTCGTTGCTCCCTCGCCTGCCGCCCTCCTCCTCCTGCGCCTGTCCTCCGCGCCGCGGCGCGTGCGTTCCCCC
CGGCCGGTGGGGTCTCTCGGCCTGGTCGTCGTTGTGCGCCTTTCCCTGCCGCGCCCCGCCCC-
GCCCGCCCGTCC-----CCGTGC---
CCGCCGGCTCGTGCTTCCCCGTGCCGCCGCCCGTCTGTGTTCCCTTCTGCCTCCCACCCCCGGCGCCTCCCCCCC
GC---

TTGA---
-------------GCGGGTGGGGGCGGGGTGGGGTGGGG-TGGGGGAGCCAGGCGGGTGGAGGCT-
GGACCGGGGGTCGGTCCTCGCGCGTG------GCTCTCGTACCGTGTCCC------CTTCCCACC----------
TGG----CGGGT-GCCCTGTTCCCC-
GCGCGTGGGCGAGCGAGCGAGCGAGCGGACGAGCGAGGGGGAGCCGATCGGGC----------------------
----

--------------------------------------TG-
TCCGCGCCCGTGTCCCATCTGTCCCGTCCCGCCCGCCCGCCCGCTTCGCCCTCCGC--CCCTCCGAGAC---GC-
GACCTCAGATCAG

Tursiops
TCAAT--CGCCCCCC--CGGGG------
TGTGTGTGCGC--
---GCGCGCGCGCCTTCCC-GGGGGT---
GGCG-CGGCT-GGGGGTTT--
GCTCGCAGGGCCGCCG---

GGGCCCTCCGTCCCCCTAAGTGCAGACG------

CGGCGCCCCCCGTCC--

-------------------ACGGGGGCGCT---------------GCCCGCGAGCGGGAAGAGAAGGGGGG-
AGAGAGAGA----------GAGA------------GTGA---CGAGGCCC---
TGGCCGCGTGG--

-----------------------------------CCTCCGCGGTCGGTC--CCCCT-TCGGGA-AGCG---
CCCTCGCGCCGCA----AG----CGGTCTC--------TGGGC----GCTACCCC------
CGGGGTGTCGGTGGGC-GGGGACGGTCCC------GCGCCCTCGCGGCCGGGGCC---GGCGGCGGT---
GGTGGGGCCGCGTCCGTTCC--------GCCGTC-GT---
ACGCCGCC---

CGCCCCC--

GGGGCGTCCCGGCGAGGTCCGGCCCGCCTCCTCCGCGGGGCCCCGACCCGCGCGTCCGGGGTCCGTGCCCGCCCC
CGCCGCCT---CG-CCTC---GCCGTGTCGGGGCGGGC-GTC-TCGGGCC-GAGG--------
CCGAGTC----------------GTGCGTGCGCGG---CCGCGCCCC---GGGG-----ATGCGT-GCCCC-
GGCGG-CGA-CCCGC-GG-----GACGCC--GCGGCGTC-GCCTGCCGCC---------
GCGCGCTTTCC---------------CCCGGGC--------------TGCGGCCGCG-CCGCT-CGGCGCTT--
CGTGCAC--GCTCCCCGAGCC---GGCCGGCGGTGGGCGCCTGT-
CGGTGGCGGGCGGGGGCGCACGGCGTCGTCCGTCCGTCGCTCGTGGTGGCGGGGCCGTCTCGCGGCTGCGTGGAG
GCTGTGTGGCGTCGGCGGCGTGAGGGGTGGAGAGGCGGAGAGCGGAGACGGGGGGAGAGAGAGGGGAGAGCGAGA
GAGGGTTGGGAGTCGGTCGGTTCGGTCGGTCGCGGAAGGAGGCGCGGGAGGAGGGCCCGGCGGTCG-
GGGGGCGACCGCCGGGTTTTCTCCCCACCGCCT-
TGCCTTCCGCGCCGCCCGCCGCCCTCTCCCGTCCGCTGCTCCCGCTCCTCCTCCTCCTCGTCCTCCCCGTCGTCC
TCTCCCGTCCCCCCGCCCCCACCCCCGTCGTCTGCCGTCCTCCCCTCCGTGACGGCGCCGGCTCTGCGGCGCGCT
CGGGTGTCGTGCGTCTCCCGGCCGCCACCCCCGGCCTCCCCGCCCC-GTCCGTCCGTCC--GTCCCGTGG---
CCGCCGGCTCGTGCTCCC--------------
GTCCCGCCCCTCCCTCCCTCCCGCGCGTCCCTCTCTCCGCCCCCC------------------------------
----
CCGCTCAC---
---------------------GGGGGGCGGGTTGAGGGGGAGCCGGTGTGGGGGGGGTGGGGCCGGTC--
GACCGCGGCTCGGCCGCGGG-----------GTCTCTCTCTCCGTCCCTCCTTCGCCCGCTCGCCCC---
GCTCTCG-CGCGC-GCCTCCGAGG---------
CGCGCG---

---------------------CCCTCCGAGAC---GC-GACCTCAGATCAG

Bos
TCAAT--CGCCCCTC--CGGGG------

TGCGCGTGTCC--
--TGTGCGCGCCTCCCCGGGGGGC----
GCG-TGGCT-GGGGGCTGT-
CCTCGCAGGGCCTCT--

GGGCCCTCCGTCCCCCTAAGTGCAGACA------
CGGCGTCCCCCCCCCCCGCCTCT--

--------------GTGGC-CGGGGGGACGTC-------------
GCCCGCGAGAGGGGAGAAAAGGGGA--------------------CGTGA--------G-TCGCG---
CCGAGGCCCC--
TGGCCTGCCGGGG--

-----------------------------------CCTGTGGGGTCGGTC--TCCCCTTCGAG--AGC---
TTCCTCGCGCCGCA----AG----CGGCCTT--------CGGGT----GTCACCGCCCC---
TGGGGTGTCGGGGGGCTGGGGACGGTCTC------GCGCCACC-
CGGCGGGGGCCCGCGGTGGTGGCGGGGGTGGGGTCGCGCCC-----TCCGGTCCGCCGTC-GTT---
CGCCGTCCCGCC---

CTT---
GGCGGTGGTGTTTGCCGGCGCCCGGCCGGCGCGGCCCTCCGCCCCCCGCCTTCCCCCTGCGGTG-
CCTTTCCCGCCGTGTGCCCGCGGCCCGTGCCCGCTTCC-------------CGCCCCCACGCGG-------
CTC-GCGCCCCGTCGGGACGGGC-GGC-TCGCGCCCGAGG--------
CCGAGTCC--------------------GCGTGCGCGTCCGCGCCCC---GGGG-----ACGCGT-GCCCC-
GGCGG-CGAC-CCGC-GG-----GACGCC--GCGGCGTCTGCCCGCCGCT---------
GCGCGCTCTCC--------------CCCCGGGC--------------TGCGGCCGTG-CCG---CGGT-----
TCGCG-------CCCCGGAGCTCCC-GCCGC---GAGGGCGGTGG-CGGTGGCGGTAGCGTGGTGA------
GGGGGGGGG-GGT------GGTAGGGGGC-----
GCGCGGTGCGTCCGTCGCCC---

GTTC---

----------GGGCTTCGGCGCCCGTGCGTGCCCCGACCCGCCCCCTCCC--CAACCC----
GGGCCGCCTCTCCGCCGGTCCCCGCCCC---GTCC--GTCCCGTGA---
CCGCCGGCTCGTGCTCCCC-------------GTCCCGCCCCGCCTCGGTGTCTCCCTCTCTGTCCGC-
CGCCCGCGGCCGCCGCCGCC---
-TCCTCC---TCCC------------
GGAGGGGGCGACGGGTGG--
TAGGCGGTGGTCGGTAGGGGTGCTGTGTTGGTGCGCGGGCAAGGGGGGGC---GGCC-CGGG-GCGG-
TCGGCCGCGG------------GTCTCTCTGTCCCC-TTCTCGGGACCCTC-------------CTCG----
CGGGT-

GCCCCCGCTGCGCGCGCGCGCGC--

----------------------------------GCGCCCTCCGAGAC---GC-GACCTCAGATCAG

Bubalus
TCAAT--CGCCCCTC--CGGGG------
TGCGCGTGTCC--
--CGTGCGCGCCTCCCCGGGGGGC----
GCG-TGGCT-GGGGGCTGT-
CCTCGCAGGGCCTC---

GGGGCCCTCCGTCCCCCTAAGTGCAGACA------CGGCGTCCCCCCCCC-
GCCTCT---

GTGGCCCGGGGGGACGTC-------------GCCCGCGAGAGGGGAGAA--
GGGGA--------------------CGAGA--------GCTCCGG---CTGAGGCCCC--
TGGCCTGCCGGG---

----------------------------------GCCTCCGGGGTCGGTCTCTCCCCTTTGAG--AGCG---
TCCTCGCGCCGCA----AG----CGGCCTT--------CGGGT----GTCACTGCCCC---TGGGGTGTCGGGG-
GCTGGGGATGGTCTC------GCGCCACG-CGGCGGGGGCCCGCGGTGGTGGCGG--
GTGGGGTGGGGTCGCGTCCTCCGGTCCGCCGTC-GTT---
CGCCGTCCCGCC---

CTC--
GGCGGCGGTGTTTCCCGGCGCCCGGCCGGCGCGGCCC--------------------------------------
----
--NNNNNNNNNNNC-
GCGCCCC---GGGG-----ACGCGT-GCCCC-GGCGG-CGAC-CCGC-GG-----GACGCC--
GCGGCGTCTGCCCGCCGCT---------GCGCGCTCTCC--------------CCCCGGGC--------------
TGCGGCCGTG-CCG---CGGT-----TCGCG-------CCCCGGCGCTCTCGCCGC----GAGGGC-
GTGGTCGGTGGCGGCGGTGGCGGA-----GCGGAGGGGAT-GTC------GGGAGGGGGC-----
GCGCGTTGCGTCCGTCGCCC---

GTTC---

----------GGGCTTCGGCGCCCGTGCGTGCCCCGACCCTTCTTCCCCCCCCGCCCCGCGGCCACCTCG---
CCGCCGGCCCCCGCCCC---GTCC--GTCCCGTGG---CCGCCGGCTCGTGCTCCCC-------------
GCCCCGCCCCGCCTCGGCGTCTCCCTCTCTGTCCGC-

CGCCCGCGGCCGCTGCCGCC---
--GCCGCCGCCGCCGCCTCCTCCTCCCGGAGGG-
GCGACGGG--
TAGGCGGTGGTCGGTAG--GGTGCTGTGTGGGTGCGTGGGCAAGGGGGGGCCC--GGGGGGG--GCGG-
TCGGCCGCGG------------GTCTCTCCGTCCCCCTTCTCGGGACCCTC-------------CTCG----
CGGGC-
GCCCTCGCTGCGCGTGCGCGTGC--

--------------------------------GCGCGCCCTCCGAGAC---GC-GACCTCAGATCAG

Sorex
TCAAT--CGCCCCCCCGCGGGG------
TGCCC--
---GCACTCCGCGGGGGC----
GCG-CGGCT-
GGGGGTTTCCTCTCGCAGGGCCCGCGGCC--

TTCCCGGTCGCGGTGTCCCTCCGTCCCCCTAAGTGCAGACGAGAGA-
CG---

-----------------------------GCCAT-----------
CCCGCCGAGAGCGGGGGTGGAGAGCA-------------------CGGAGC-------G-
TCGCGCCGCCGAGGTGCC--
CGCGGCCGCC---

-------------------------------ACGGTGCGTCC---CGGTC-----CCTTCGGGGGTCGA---
TCCTCGCGCCGCA----CG----CGGCCGTCC----GGCGGGGC-GGGTCGGGGGCTTCCGCGGGTGTTTCTC--
GCCGGGGACGGACCGCGTCCGGCCCCCCGTGCCCGCCTGCGCGCGCGGTCACCCTCCCTCCCCTCCCC-------
----

TCCGC--

GGGGAGAAGGGAGGGGGGGCCCGCGCGCGCGAG-TGCCCCCGTTCCCC-----------
GCCTGCGGTCGGCTC--GCCGGCCCCCGGCTCGTCGGCCCGGAGAGGA----
CCCGAGTCAGCC--------------GCGCGTGCGGCTCTCACGCTTCCC-GGGGGAGGGACGCGT-
GCCACCGACGGGCGAC-CCGC-GG-----GACGCC--GCGGTGCCCGCCCGCCGTC---------
GCGCGCTCCTC---------CCCCTTCCCGGGT--------------CGCGGTGATC-CCGC-----------
CGCG-----------CGGAGCGCGGGTCTCCTGACCCCTCGGGTTCGCGGCGGCCGGGGTGTTCCGG-CC-
CGGCCGC--
GCCT---

---CCGCCTC--ACGCGGCGGT--
CCGCCGGCCCGTGCTCCCC-GTGC--------
GTCCGCCCTCGGGC--

TCCG--
----------GCCCGCGGGCGGGCGTCGTCGGACCGGCCCGTGGCCTCGAGTT-CGCCGC---
CCCCGGGGGGCGGGTCGGCCTCGC------------GCCGACCCCGATTCCCCCCGGGG--
CCT----------------------CGGG--CGGTCCGTCGCTCCGTTCCTCGCCTCTCCTCTCC---
ACCTCCCGCCTCCCGGCG--ACGTTTTCTCTCTCTCTCTCTCTCC-
CGAGCCTCGGGT--

-----------------CTCCGGGCTCGGCTTTCCGAAGC---GC-GACCTCAGATCAG

Erinaceus
TCAAT--CGCACCCC--TGGGGT-----
TGCC--
---GGGGGT---
TGCG-CGGCT-
GGGGTTTCCCTGTCGCAGGGCCCGTG--

CTGGGGGCCCTCCGTCCCCCTAAGTGCAGACGCC----TGGCGGGGTGCTGGGG-
GCCTTT--

GGTGCTCCTCCCCCCCGCC-------------GCCCGCGAGAAGCGGG-
GTCCGTGAGGAC---------------
GCGAGAG---

------------------------------------CTCGCGCCGC-----------CGGTGCTCGAGAC------
CACGCCGCGGGGC-
GCGGTCGGGTTTTCCTCTGGGCGGTTCCCTTGCGGGGTCCGCCCGGCCCTCGGCGTGTGGGGGTCGTGTGGCTGT
CGCCTGTGTCGTGTGATCGCGCGTCACGCGCTGGGCGTGAGACCGGTTCTCTCCCCCGCGGCTGGGGGGGGCCTG
CGGCTGCGGCCCGGGCGCTCGTGCGTGCGTGTGGTCCTACCCGCTCCGGCGGAGGGTTGTGTGTGTGTGTGTGGG
GAGAGAGAGTTGGCCGCCCG----------GTC-
GCACCGGGGGGTTTTGCCCGCCCCGGCGGCCTCCTGGCCGTCCGGTCGTCCGGATCCCTCCCTCCCTCCCTCCCC
GCCTGCCCGCCGTTCGGGTCCCAGCCGCCGCCGCTGCGCGCGGCCGCCCCGCGTCCCTCTCTGCCGCGCGCG
CTGGTTTGCCGGTCTTACGCCCCGCGTCTCTGTCGCCGTCGCCGGCAGGCGACCTCCCTCTGTTCCCCCGCCGCC

GCCG----CCGGGCCGAGGGTCCGA---CTCGCGCGCGAGCGC-GGCCGTGCCCC---GGGG-----
ATGCGTCGCCCC-GGCGGGTGGC-CCGC-GG-----GACGCC--TCGGCGTC-GCTCGCCGCC---------
GCGCGCCTGC---------------CCCCGGGC-------------CTCGAGCCGCG-
CCGCGTCGGTGCCTCTCCTT--------
CCCCGTCGTGCGG--

------------------------------TCGGCCTC---------
GCTCCGCGCGGTGTGTGTGGCGC--------------
GCTCGCCTCTCTCGCCTGTGGTGTGTGTG----------------TGTGGCGC-
CTGTCTCCTCGCGCGCGCGTGCGTGCC--

---------------------------------------TCTCCGAGAC---GC-GACCTCAGATCAG

Macropus
TCAAT--CGCCCCGT--CGCGGTTTCC-
CGTTC--
---GCCGGGGACGCGGTCGG---
GGCG-CGGCT-GGGGGCG----
CTCGCAGGGTCCCGCCGCGCCTT--

GGAGGTCGTCGGCCCGGCCCTTCGTCCCCCGAAGCGCAGACG--------
GT---

GGCCGGAGGAGAAGGAAGGAAGGAAGGGAGGGGGGTNNNN----------------
CTCGCCCC---

GTCTCCCCTCCGCCTCCTCCGCCTCCCCCCGTCTCCCCTCCGCCGTCCCTCCGGCGGCGGCGGCGTCCGCCCGCC
CGCCCGCCCTCGGGG--------
ACCTCGCGCCCTC--

----------------------------------GCTC---TCCTTCCCCCCCCC------------------
CCCCCCCGCCTCCCCGCGGTCCGG---------------------------CCGGAGCGTCAGCC----------
GGGGCCCC--GGGG--CCCAC-GGTGGTTGCCT--GCGGGTTCGCTGGGC---ACGGCGCT--
TCCGCCGCGA-----------------TG-TGGGTCT------TCCCGGGTCGG------
GACACCCCGGCCGTTCCGGGCGATCCGGATCGCGCGGGGTCGGGCGGGGGGGG----------------------
----

---CACGTTCG-----------
TCCCCGCCCG---------------------------CCGAGGGAGCCCCCCCC--
GCTCCCTCGTCGGGGCGCTCGTCCTCTCCTTCCGAACTTTCCCCTCGTTTTCCT---------------------
----

-------------------TCTC-GAGAGCGTGC-GACCTCAGATCAG

Monodelphis
TCAAT--CGCCCGGTTCCC--GTTTCC-
CCCGGTACCCCTTCCGCCTCCCCCATCCCCGCCCAAGCCGTTGTCGCTCGCGAGGAGGATCCGCGGTGGATCCT
GAGGGCGCCCGCGCGTTTGGAGCTGGGGGGGGGGTTCGCGGTCGGGGTTTTCCGGGGTCTCGGGGGCGTGGGGCG
-CGGCT-GGGGGTC----
ATCGCAGGGTCCCGCCACGCCAGAGG---

CCAAAGGGTGGCCGGGGCCCTCCGTCCCCTGAAGTGCAGACG--------
GT---

--GGC-
GAAAAGATGAAGCGAGAAGGGCCGCCCGCGTG------GCGGAG--------
CTCGCCCGCC---

---CTTGGGG--------ACCTCGCGCCCTC--TGCGGG--

```
CGGCGTCTGGACCCGCCGGGCGTGCGGTGCGGTGTGTCGCGTGGGGTTTTGGGTACGCTGTCCCGGACCACCGGG
CGCTTTCCC------------------------------------------------------------------
---------------------------------------------------------------------------
---------------------------------------------------------------------------
---------------------------------------------------------------------------
---------------------------------------------------------------------------
-----------GCTCCCCTCCTTCCCCCGCCCGCTCGTCGTCGGGCGGG--CCCCCT---TCCCGTCGG-
CCGG---------------------------ACGGAGCGCCTGTC----------GGGGCCC-----GGC-
CCCGC-GGCGGTTGCCT--GT-GGT-CGCTGAAC---ACGGTGCT--TCCGC-GCGG----------------
TTGTTGGGG---------CCCGGGT-GG------GACACCCCGACCGTTCCGC----TCCGTGCCGAGCGGG-
TCGG-----------------------------------------------------------------------
---------------------------------------------------------------------------
---------------------------------------------------------------------------
---------------------------------------------------------------------------
---------------------------------------------------------------------------
---------------------------------------------------------------------------
---------------------------------------------------------------------------
---------------------------------------------------------------------------
---------------------------------------------------------------------------
---------------------------------------------------------------------------
----AGCCGC-TGGCACGT-CGGT---------TTCCCGCCTGA---CTCGTTGC-----------
CGGGCGCGCAGAGCT----
CGGAGTTCTCCCTTCCTCGCGTGCCGCCTTCCCTCT-------------------------
CTCCCACCCTT----------------------------------------------------------------
---------------------------------------------------------------------------
---------------------------------------------------------------------------
---------------------------------------------------------------------------
--------------------TGGGGTGGGGGTTCCTTCCTCTGTGAT---GC-GACCTCAGATCAG
;
end;
```
